# Synthesis of new *p-tert*-butylcalix[4]arene-based polyammonium triazolyl amphiphiles and their binding with nucleoside phosphates

**DOI:** 10.3762/bjoc.14.173

**Published:** 2018-07-31

**Authors:** Vladimir A Burilov, Guzaliya A Fatikhova, Mariya N Dokuchaeva, Ramil I Nugmanov, Diana A Mironova, Pavel V Dorovatovskii, Victor N Khrustalev, Svetlana E Solovieva, Igor S Antipin

**Affiliations:** 1 Kazan Federal University, 18 Kremlevskaya st., Kazan 420008, Russian Federation; 2 National Research Center "Kurchatov Institute", 1 Ak. Kurchatov Square, Moscow 123182, Russian Federation; 3 Peoples' Friendship University of Russia (RUDN University), 6 Miklukho-Maklay Street, Moscow 117198, Russin Federation; 4 A. E. Arzubov Institute of Organic & Physical Chemistry, 8 Arzubov Street, Kazan 420088, Russian Federation

**Keywords:** ADP, amphiphile, ATP, calix[4]arene, CuAAC, eosin Y probe, molecular recognition, polydiacetylene, self-assembly, triazole

## Abstract

The synthesis of new calix[4]arenes adopting a *cone* stereoisomeric form bearing two or four azide fragments on the upper rim and water-soluble triazolyl amphiphilic receptors with two or four polyammonium headgroups via copper-catalyzed azide–alkyne cycloaddition reaction has been performed for the first time. It was found that the synthesized macrocycles form stable aggregates with hydrodynamic diameters between 150–200 nm and electrokinetic potentials about +40 to +60 mV in water solutions. Critical aggregation concentration (CAC) values were measured using a micelle method with pyrene and eosin Y as dye probes. The CAC values of tetraalkyl-substituted macrocycles **12a**,**b** (5 µM for both) are significantly lower than those for dialkyl-substituted macrocycles **10a**,**b** (790 and 160 µM, respectively). Premicellar aggregates of macrocycles **10a**,**b** and **12a**,**b** with the dye eosin Y were used for nucleotides sensing through a dye replacement procedure. It is unusual that disubstituted macrocycles **10a**,**b** bind more effectively a less charged adenosine 5'-diphosphate (ADP) than adenosine 5'-triphosphate (ATP). A simple colorimetric method based on polydiacetylene vesicles decorated with **10b** was elaborated for the naked-eye detection of ADP with a detection limit of 0.5 mM.

## Introduction

During the last two decades many researcher groups have paid much attention to the synthesis of host molecules with high affinity to biologically important anions [1–5]. Among these anions, nucleotide recognition and sensing represents an especially important research area due to the great biological significance of these anions. Adenine-containing nucleotides are very important as a universal energy source and as intracellular mediators in many biological processes [6]. In the cellular metabolism, adenosine 5'-triphosphate (ATP) is hydrolyzed to adenosine 5'-monophosphate (AMP) or adenosine 5'-diphosphate (ADP) by enzymes [7]. Thus, the receptors for nucleotides must possess selectivity towards these anions. From this point of view, nucleotide receptors based on polyammonium cations are of great demand because the electrostatic interactions of such polyammonium systems and negatively charged phosphates are strong. Hydrogen bonding [8] and π-stacking interactions between the adenine groups of the nucleotide and the receptor’s aromatic moieties [9] can additionally contribute to the complex stability and binding selectivity. Polyammoniums mimic the biologically important acyclic polyamines such as putrescine, spermidine, and spermine, which have strong affinities to AMP, ADP and ATP [10]. Usually polyammonium cation receptors bind the nucleotides according to their negative charge values: ATP > ADP > AMP. It is accompanied with increasing complex stability, which depends on charge–charge interactions between the receptor and the nucleotide. Only a few publications have been reported about receptors that more effectively interact with less charged nucleotides. For instance, Kuchelmeister et al. synthesized a receptor with two symmetric peptide arms decorated with guanidinium-based anion binding sites. This receptor showed a stronger binding of AMP in comparison with ADP and ATP [11]. Another polyammonium receptor synthesized by Mascaros et al. [12] showed selective recognition of ADP in the presence of ATP in water.

Undoubtedly, macrocyclic receptors have a number of advantages in the design of molecular receptors, providing preorganization of the binding sites offering multipoint interactions with a substrate for the effective complexation [13]. Calix[4]arenes and their thia analogues have many advantages over other macrocycles that are frequently used as synthetic receptors, such as cyclodextrins [14], cucurbiturils [15], and pillararenes [16]. Calixarenes are easily functionalized at both their upper and lower rims with various stereoisomers obtainable; the initial macrocycles can be synthesized in a simple manner, they are not toxic, etc. [17–22]. Amphiphilic calixarenes are particularly interesting because they can be regarded as surfactants having a host–guest recognition site [23]. So, they are able to form nanoaggregates in aqueous solutions, thus providing the concentration of the active binding sites into nanoaggregate for multivalent binding with the substrate [24]. We recently synthesized a series of cationic receptors based on the *p-tert*-butylthiacalix[4]arene platform with *1,3-alternate* conformation having polyammonium binding sites. They were shown to effectively interact with calf thymus DNA causing a 4-fold compaction of the latter [25]. Related macrocycles containing two cationic imidazolium fragments demonstrated a high affinity to ATP [26].

Herein we report the synthesis of new amphiphilic water-soluble calix[4]arene derivatives with *cone* conformation containing two or four polyammonium groups on the upper rim by using a click chemistry approach and the results of binding studies toward nucleotides in aqueous solutions.

## Results and Discussion

### Synthesis of polyammonium calix[4]arene derivatives

**Scheme 1:**
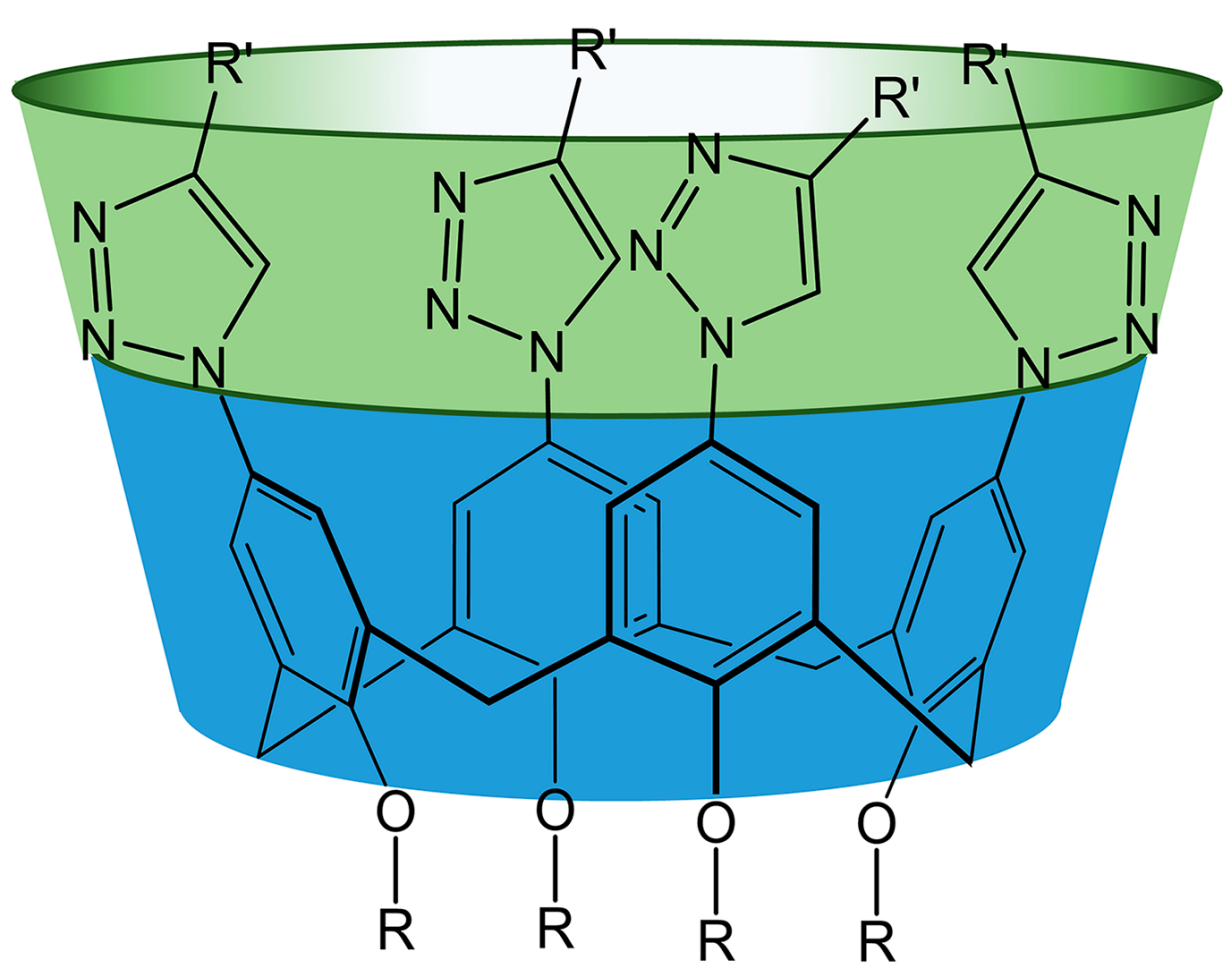
The general structure of triazolylcalix[4]arene derivatives.

The functionalization of calix[4]arenes with azide groups paves the way to introduce a wide variety of functional groups [27] on the upper rim of the macrocycle by, e.g., the copper-catalyzed azide–alkyne cycloaddition (CuAAC) reaction [28]. An alternative way is the functionalization of calix[4]arenes by terminal alkynyl groups. However, in this case further transformations by CuAAC reactions are limited mainly due to the fact that low molecular weight organic azides, especially containing less than 3 carbon atoms are highly explosive [29]. Usually azide groups are installed in the upper rim of the macrocycle by a chloromethylation reaction and subsequent nucleophilic substitution by azide anions [30–31] forming rather flexible azidomethyl fragments. In this investigation more rigid arylazide calixarene derivatives were chosen as precursors for the synthesis of the targeted macrocycles having an enlarged cavity for the effective binding of large biomolecules (Scheme 1).

**Scheme 2:**
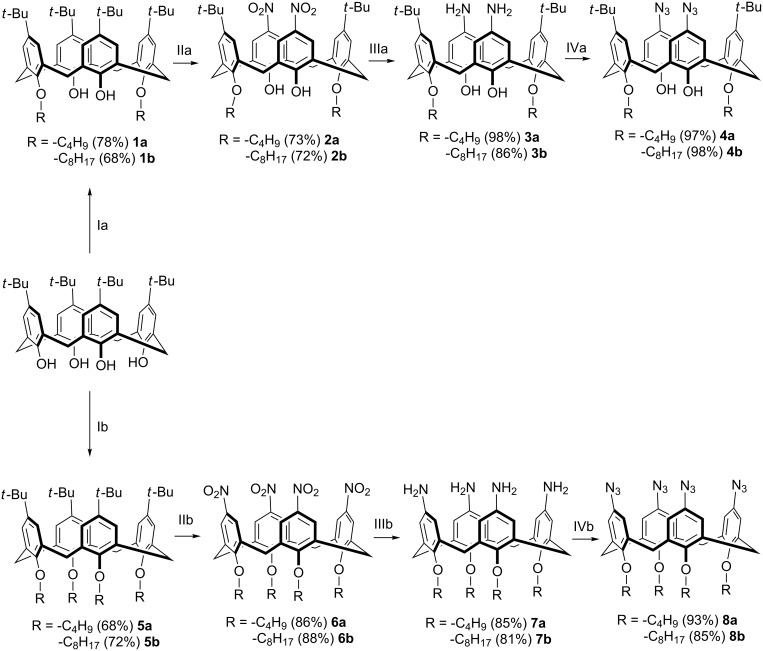
Synthesis of di- (**4a**,**b**) and tetraazido (**8a**,**b**) calix[4]arene derivatives. Conditions: Ia: AlkBr, K_2_CO_3_, acetone, 130 °C, MW heating 400 W; IIa: HNO_3_, CH_2_Cl_2_, 0 °C; IIIa: NH_2_NH_2_, Ni/silica/alumina, EtOH, reflux; IVa: NaNO_2_, AcOH/DMF, 0 °C, NaN_3_; Ib: AlkBr, NaH, DMF, rt; IIb: HNO_3_ (100%), AcOH, CH_2_Cl_2_, 0 °C; IIIb: NH_2_NH_2_, Ni/silica/alumina, EtOH, reflux; IVb: NaNO_2_, AcOH/DMF, 0 °C, NaN_3_.

The targeted calixarene diazide derivative **4** and the corresponding tetraazide **8** were synthesized as outlined in Scheme 2. In the first step the complete O-alkylation of the parent *p-tert-*butylcalix[4]arene was performed to give the products **5a**,**b**. To achieve the selective distal alkylation of the macrocycle’s lower rim (compounds **1a**,**b**) a microwave approach developed in our group was applied [32]. Then the di- and tetraalkylated products were nitrated according to literature procedures [33–34] affording the di or tetranitro derivatives **2a**,**b** and **6a**,**b** in good yields. The reduction of the nitro groups to the corresponding amines was successfully performed by hydrazine hydrate [35–36]. In this case, Ni on silica/alumina was used as the catalyst instead of pyrophoric Raney nickel. Finally, a diazotization procedure with subsequent azide substitution [37–38] gave calix[4]arene azide derivatives **4** and **8**. For the latter reaction a mixture of DMF/glacial acetic acid 3:1 was found to be the optimal solvent.

The structures of macrocycles **4** and **8** were established by ^1^H and ^13^C NMR spectroscopy as well as by IR spectroscopy and MALDI–TOF mass spectrometry. Their compositions were determined by elementary analysis. A standard set of signals typical for distal disubstituted calixarenes was found in the ^1^H NMR spectra of **4a** and **b** (Supporting Information File 1, Figures S5 and S6): a singlet of OH protons at 8.33–8.37, two singlets of aromatic protons at 6.95–6.96 and 6.69–6.70 ppm, doublets of bridged CH_2_ fragments at 4.31–4.32 and 3.31 ppm. In the case of tetraazides **8a**,**b** (Supporting Information File 1, Figures S7 and S8) the signals of the aromatic protons appear as a singlet at 6.29 ppm. The signals of the bridge methylene protons appear as two doublets at 4.39–4.40 and 3.09–3.10 ppm. The presence of the azide groups was confirmed by valence asymmetric bond vibrations at 2109 cm^−1^ in the IR spectra of **4** and **8**. The MALDI mass spectra of all obtained azides gave molecular ion peaks with expulsion of two (in the case of **4**) or four (in the case of **8**) N_2_ fragments due to the lability of the azide group upon laser desorption [39].

**Figure 1:**
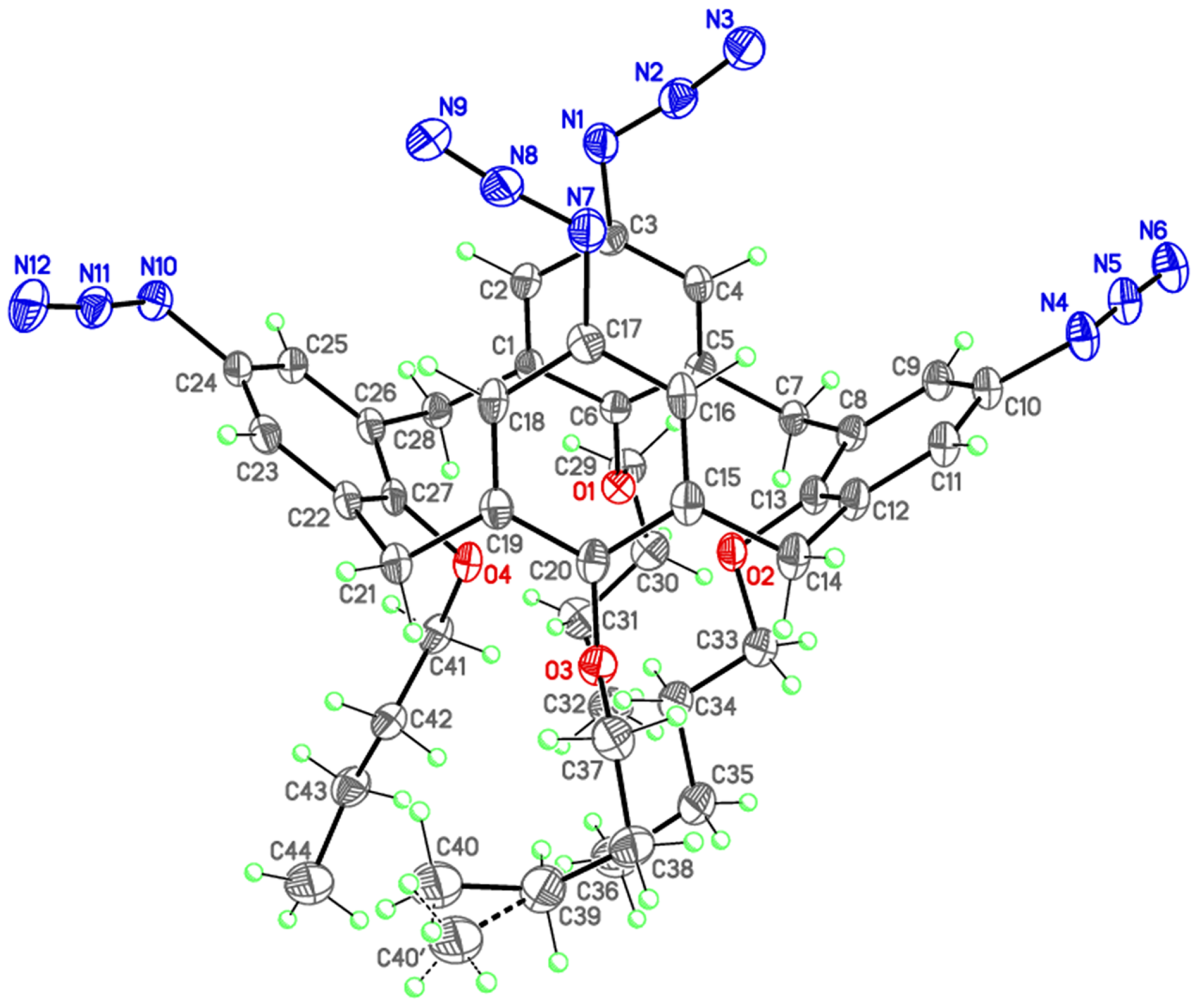
Molecular structure of **8a** (50% ellipsoids). The dashed line indicates the alternative position of the disordered *n*-butyl group with the minor occupancy.

The spatial structure of the product **8a** was established by X-ray crystallography and is presented in Figure 1 including the atomic numbering scheme. The full X-ray crystallographic data are provided in Supporting Information File 1. The molecule of **8a** adopts a pinched-cone conformation as evidenced by the differences of the interatomic distances between carbon atoms C3···C17 (4.018 Å) and C10···C24 (10.051 Å). A similar conformation is adopted by calix[4]arenes containing non-bulky substituents at the upper rim [34].

**Scheme 3:**
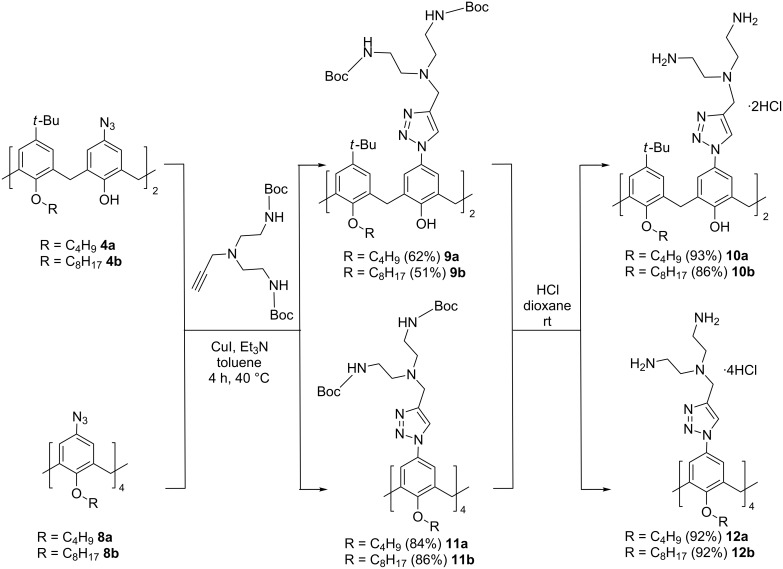
Synthesis of polyammonium macrocycles **10a**,**b** and **12a**,**b**.

Then, azido compounds **4a**,**b** and **8a**,**b** were subjected to copper-catalyzed reaction with 3-bis[2-(*tert*-butoxycarbonylamino)ethyl]propargylamine (Scheme 3). The syntheses were carried out for 4 hours at 40 °C in toluene and the target BOC-protected products **9a**,**b** and **11a**,**b** were isolated in good yields. The appearance of a new signal of triazole ring protons and a new set of signals of the methylene group protons between the triazole ring and the tertiary nitrogen atom as well as the signals of the ethylene fragments and BOC groups in the ^1^H NMR spectra fully corresponded to the proposed structures of **9** and **11** (Supporting Information File 1, Figures S9, S10, S13, and S14). The polyamines **10a**,**b** and **12a**,**b** were obtained in high yields after BOC deprotection with HCl in dioxane as water-soluble di- and tetrahydrochlorides, correspondingly.

**Figure 2:**
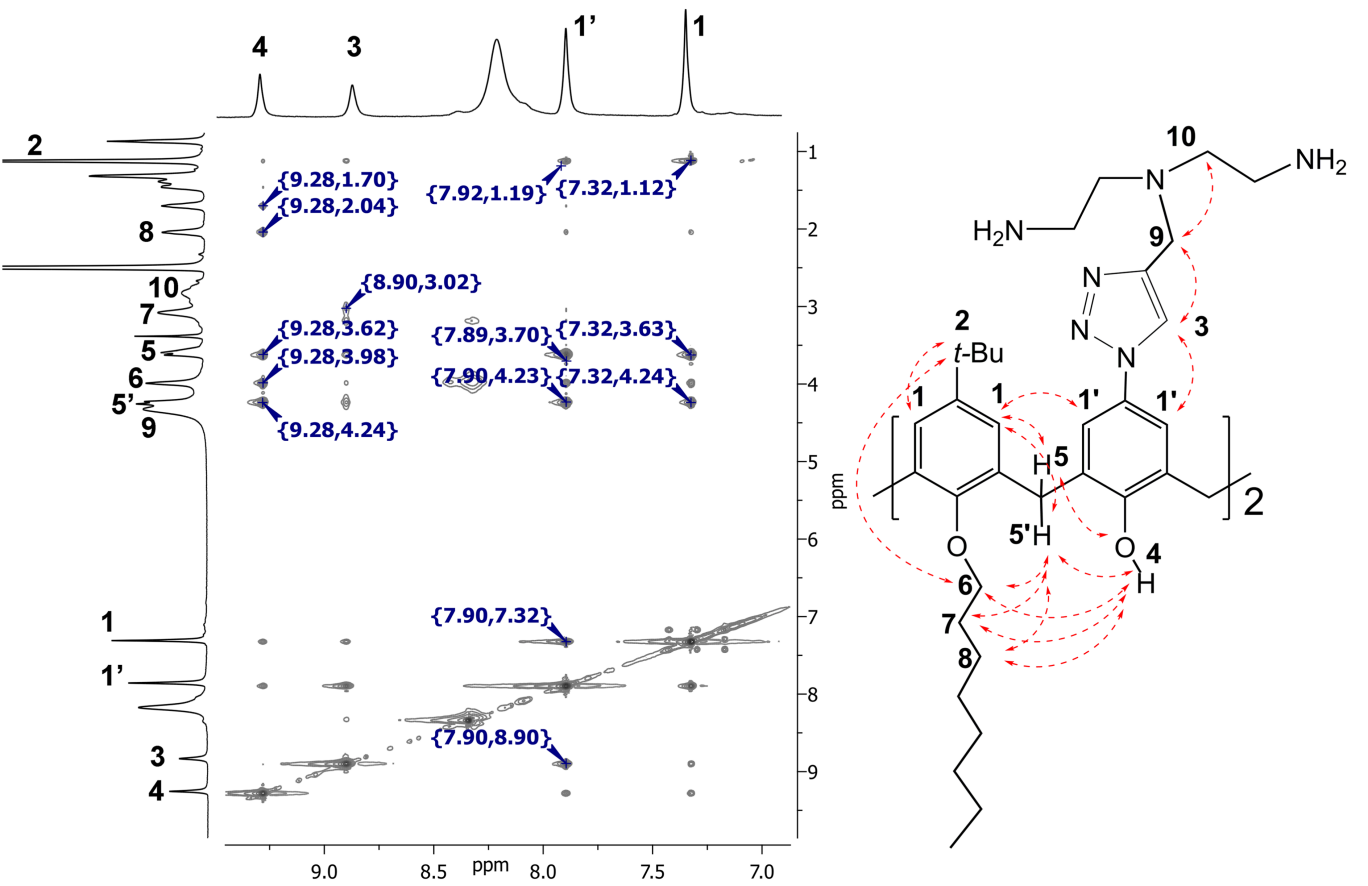
2D NOESY H^1^-H^1^ NMR spectra of **10b** in DMSO-*d*_6_.

The structures of the final products were established by 2D ^1^H-^1^H NOESY NMR. For example, in the case of macrocycle **10b** (Figure 2) the cross-peaks between signals of neighboring aromatic protons (δ = 7.32 and 7.92 ppm) indicate a cone stereoisomeric form of the macrocycle. Moreover, the observation of cross-peaks between hydroxy protons 4 and the methylene bridge protons 5 and 5’, on the one hand, and the methylene protons 6–8 in the octyl chain (δ = 3.98, 3.18 and 2.04 ppm), on the other hand, is also in line with this conclusion. Thus, the 2D NMR data are completely consistent with the proposed structure of **10b**. It should be noted that there are unexpected interactions of *tert-*butyl protons 2 with methylene protons 6 as well as between aromatic protons 1 with hydroxy protons 4 that can be attributed to a strong aggregation of the amphiphilic molecules in the solution.

### Aggregation properties of **10a**,**b** and **12a**,**b** in aqueous solutions and binding with nucleotides

All synthesized derivatives **10a**,**b** and **12a**,**b** are well soluble in water/MES buffered solutions. We used the determination of the critical aggregation concentration (CAC) to elucidate the compounds’ aggregation behavior. This was performed by the dye micellization method using pyrene and eosin Y (EY) as fluorescent and spectrophotometric probes, respectively (Table 1). The nonpolar pyrene can be incorporated into hydrophobic domains of the aggregates due to hydrophobic interactions. Pyrene insertion is measured by analyzing the ratio of the intensities of the first and third bands (373 and 384 nm, respectively) in its emission spectrum [40]. EY, an anionic xanthene dye, also can be used for CAC determination as its absorption spectrum shows a shift of the maximum absorption wavelength (λ_max_) in the presence of micelles or vesicles [41]. The inflection point in the plot of EY λ_max_ vs surfactant concentration should be treated as the CAC of the amphiphile [42].

**Table 1:** CAC values determined by dye (pyrene and EY) micellization method.^a^

macrocycle	CAC, µМ	
	
	EY	pyrene

**10a**	6.4	790
**10b**	4.8	160
**12a**	4.5	5.0
**12b**	2.8	5.0

^a^Сoncentration (EY) = 10 µM; concentration (pyrene) = 5 µM.

As can be seen from Table 1 the CAC values determined by the two methods are significantly different, especially in the case of macrocycles **10a**,**b**. These differences are assigned to the different natures of the used dyes. In the case of EY, which is a dianion at pH 7, the interaction with the dicationic calixarenes **10a**,**b** results in the formation of a non-charged complex, which can be referred to a non-ionic surfactant. Generally, non-ionic surfactants possess lower CACs than ionic ones owing to the lack of electrostatic repulsions between head groups [43]. In the case of pyrene there is no charge compensation and interactions occur exclusively with hydrophobic domains of the aggregates. Thus, using pyrene reveals the real CAC value, while with EY the CAC value corresponds to the dye–calixarene complex. In the case of **12a**,**b** the differences of CAC values determined by EY and pyrene are not significant. Obviously, in this case EY does not form a neutral dye–calixarene complex due to the increased number of ionized groups in the tetrasubstituted calixarene. However, it is worth paying attention to the extremely large difference between the CAC (pyrene) values of **10a** and **12a** as well as **10b** and **12b**. It can be caused by strong hydrophobic intermolecular interactions of **12a**,**b**, which leads to the formation of aggregates at substantially lower concentrations as compared with the less hydrophobic **10a**,**b**. A similar trend (decrease of about two orders) was observed for the CAC of gemini surfactants possessing two hydrophobic alkyl tails in comparison with ordinary ones having one alkyl tail [44].

Additionally, the aggregation properties were investigated by dynamic (DLS) and electrophoretic (ELS) light scattering methods and the obtained results are collected in Table 2. Both di- and tetrasubstituted macrocycles do form nanoparticles in aqueous solution with hydrodynamic diameters within the range of 150–200 nm. The electrokinetic potential of the aggregates corresponds to the positive charge of the calixarene headgroups and is about +40 to +60 mV which indicates the formation of stable colloids. The addition of negatively charged EY to **10a**,**b** reduces the electrokinetic potential practically up to the isoelectric point. This confirmed the abovementioned conclusion concerning the formation of a non-charged complex as a reason of low CAC values measured by EY as the probe. A similar behavior also was observed for the tetrasubstituted derivatives **12a**,**b**.

**Table 2:** DLS and ELS data of aggregates formed by macrocycles **10** and **12**.^a^

entry	system	*d* [nm]	PDI	ζ [mV]

1	**10a**	194 ± 3	0.385 ± 0.17	+50 ± 4
2	**10b**	153 ± 18	0.419 ± 0.06	+42 ± 13
3	**12a**	176 ± 11	0.244 ± 0.04	+43 ± 6
4	**12b**	140 ± 54	0.443 ± 0.01	+64 ± 8
5	**10a** + EY	157 ± 2	0.500 ± 0,06	+14 ± 5
6	**10b** + EY	208 ± 9	0.327 ± 0.01	+6 ± 2
7	**12a** + EY	99 ± 1	0.314 ± 0.04	+24 ± 4
8	**12b** + EY	202 ± 5	0.364 ± 0.06	+14 ± 7
9	**10b** + EY + ADP	164 ± 7	0,412 ± 0,07	−6 ± 2
10	**10b** + EY + ATP	256 ± 43	0,619 ± 0,09	−20 ± 9

^a^All measurements were done in 50 mM MES buffer at pH 6.5; concentration (calixarene) = 10 µM, concentration (EY) = 10 µM (**10a**,**b**) or 20 µМ (**12a**,**b**).

Recently, we reported receptor systems for sulfonate surfactants [45] and ATF [26], based on an EY competitive displacement from aggregates, decorated by positive charged thiacalixarene macrocycles. This approach was used for macrocycles **10a**,**b** and **12a**,**b** as well. Primarily, the fundamental regularities of dye complexation with **10a**,**b** and **12a**,**b** were studied in detail. The stoichiometry of the EY–calixarene complexes was determined by the isomolar series method at two concentrations: below and above the CAC values, measured with EY as the probe (Table 3).

**Table 3:** Stoichiometry of the EY–calixarene complexes.^a^

calixarene	dye/calixarene ratio^b^	dye/calixarene ratio^c^

**10a**	1:1	3:2
**10b**	1:1	3:2
**12a**	2:1	7:3
**12b**	2:1	4:1

^a^All measurements were done in 50 mM MES buffer at pH 6.5; ^b^at total concentration = 1 µM; ^c^at total concentration = 50 µM.

At concentrations below the CAC (1 µM), the monomeric molecules of the macrocycles **10a**,**b** form with EY a discrete 1:1 host–guest complex [X^2−^calix^2+^], where X^2−^ and calix^2+^ represent the dye and calixarene ions, respectively. By increasing the concentration to values above the CAC complexes with 3:2 stoichiometry are observed. This indicates that the interaction of forming aggregates with EY relies on both electrostatic interactions with positively charged headgroups and hydrophobic interactions with hydrophobic regions of calixarene aggregates that are typical for xanthene dyes [41]. The length of the alkyl chains in **10a**,**b** does not affect the complex stoichiometry.

Compounds **12a**,**b** at concentrations below the CAC form dye–calixarene complexes of 2:1 stoichiometry [(X^2−^)_2_calix^4+^]. As in the previous case the aggregation (concentration above the CAC) leads to a change in stoichiometry with 7:3 for the less lipophilic compound **12a** and 4:1 for **12b**. Thus, the presence of four alkyl substituents on the calixarene platform significantly influences the solubilizing capacity of the macrocycles with respect to EY.

To create a receptor system based on the dye displacement in an amphiphilic host molecule/aggregate, an optimal concentration of the amphiphilic molecule should be precisely established. First, the greatest changes in the dye spectrum should be observed at this concentration. Second, the dye should not be fully absorbed in the hydrophobic domain of an aggregate to be easily released into solution after any changes that occur with the molecule/aggregate. In this context premicellar aggregates are good candidates to design supramolecular systems for recognition and sensing purposes.

Thereby, premicellar concentrations of calixarenes **10a**,**b** (6 and 4 µM, respectively) and **12a**,**b** (4 and 2 µM, respectively) were selected to design a receptor system based on the principle of competitive EY displacement by the guests. At these concentrations, a bathochromic shift accompanied by hypochromic effect in the EY absorption spectrum (Supporting Information File 1, Figure S1) is observed in addition to a change of the environment polarity due to the dye embedding into the hydrophobic domain of the premicellar aggregates [46]. The absorbance spectra of the ternary system calixarene/EY/guest have been recorded in aqueous buffer solutions and a series of nucleotides (AMP, ADP and ATP) as guests for the competitive EY displacement were tested.

It was found that the addition of increasing concentrations of ADP or ATP resulted in significant changes of the calixarene/EY absorption spectra and finally practically corresponded to free EY (Supporting Information File 1, Figure S2). This clearly indicates a dye release from the calixarene aggregates into the solution. For better visualization, the optical response (OR) of the calixarene/EY system was calculated according to the Equation 1:

[1]
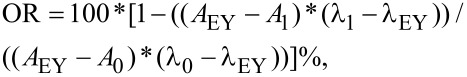


where *A*_EY_ is the free EY absorption intensity, *A*_1_ and *A*_0_ are the absorption intensities of the calixarene/EY system in the presence and absence of the guest, respectively. λ_EY_ is the wavelength of EY absorption maximum, λ_1_ and λ_0_ are the wavelengths of the absorption maxima of calixarene/EY spectra in the presence and absence of the guest, respectively.

**Figure 3:**
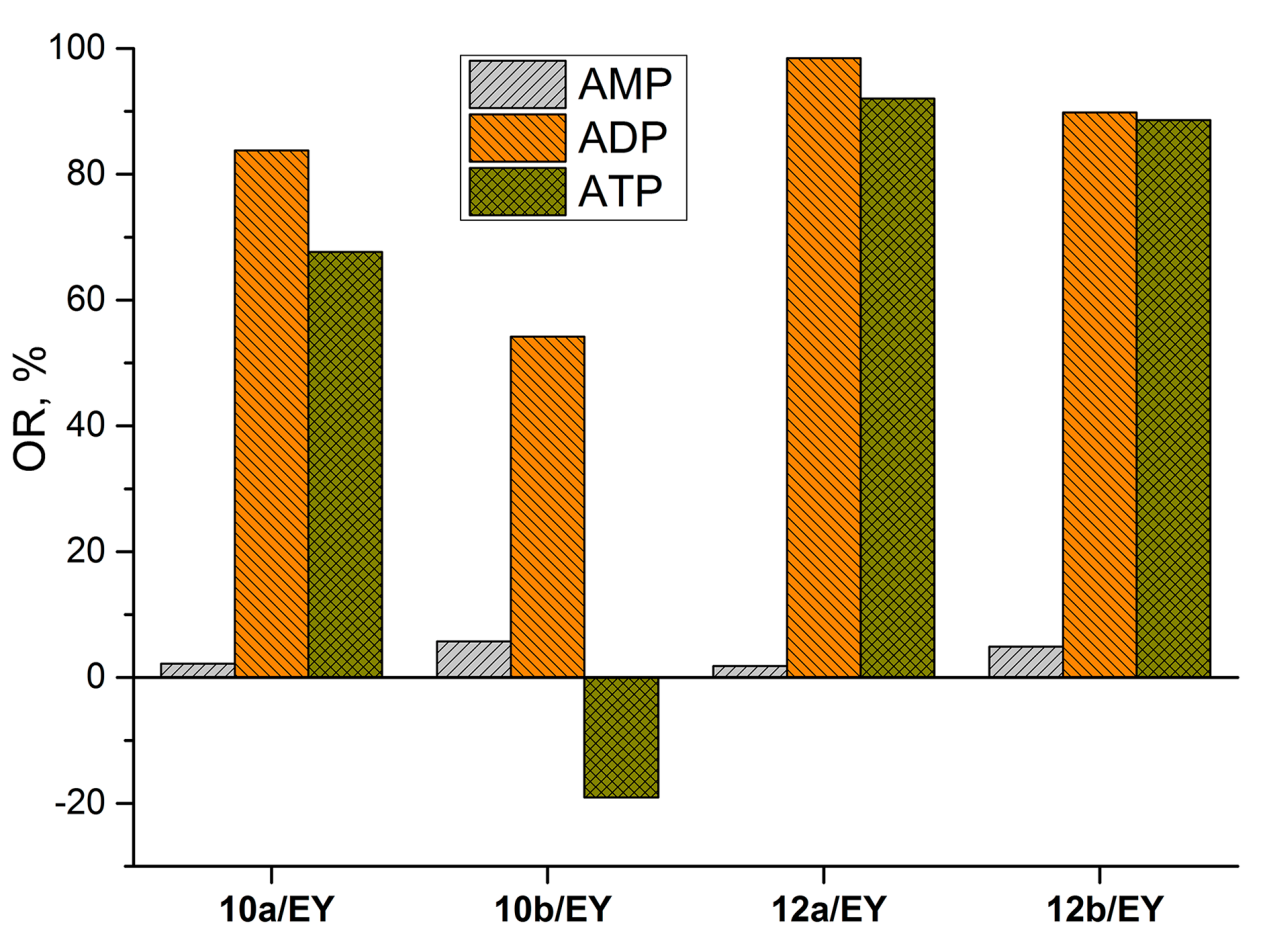
The optical response (OR) of the calixarene/EY systems toward adenosine phosphates. Concentration (EY) = 5 µM, concentration (**10a**) = 4 µM, concentration (**10b**) = 6 µM, concentration (**12a**) = 4 µM, concentration (**12b**) = 2 µM, concentration (adenosine phosphate) = 2 mM, concentration (MES) = 50 mM (pH 6.5).

In contrast to ADP and ATP, AMP had no effect on the optical response that can be explained by weak interactions of the monoanionic nucleotide with the di- and tetracationic calixarenes (Figure 3). Obviously, AMP does not effectively interact with the two distally located binding sites of the calixarenes and is not able to expel the dianionic dye from the aggregates. A quite different picture is observed for ADP and ATP, which are present in the dianionic and trianionic form at pH 6.5 [12].

**Figure 4:**
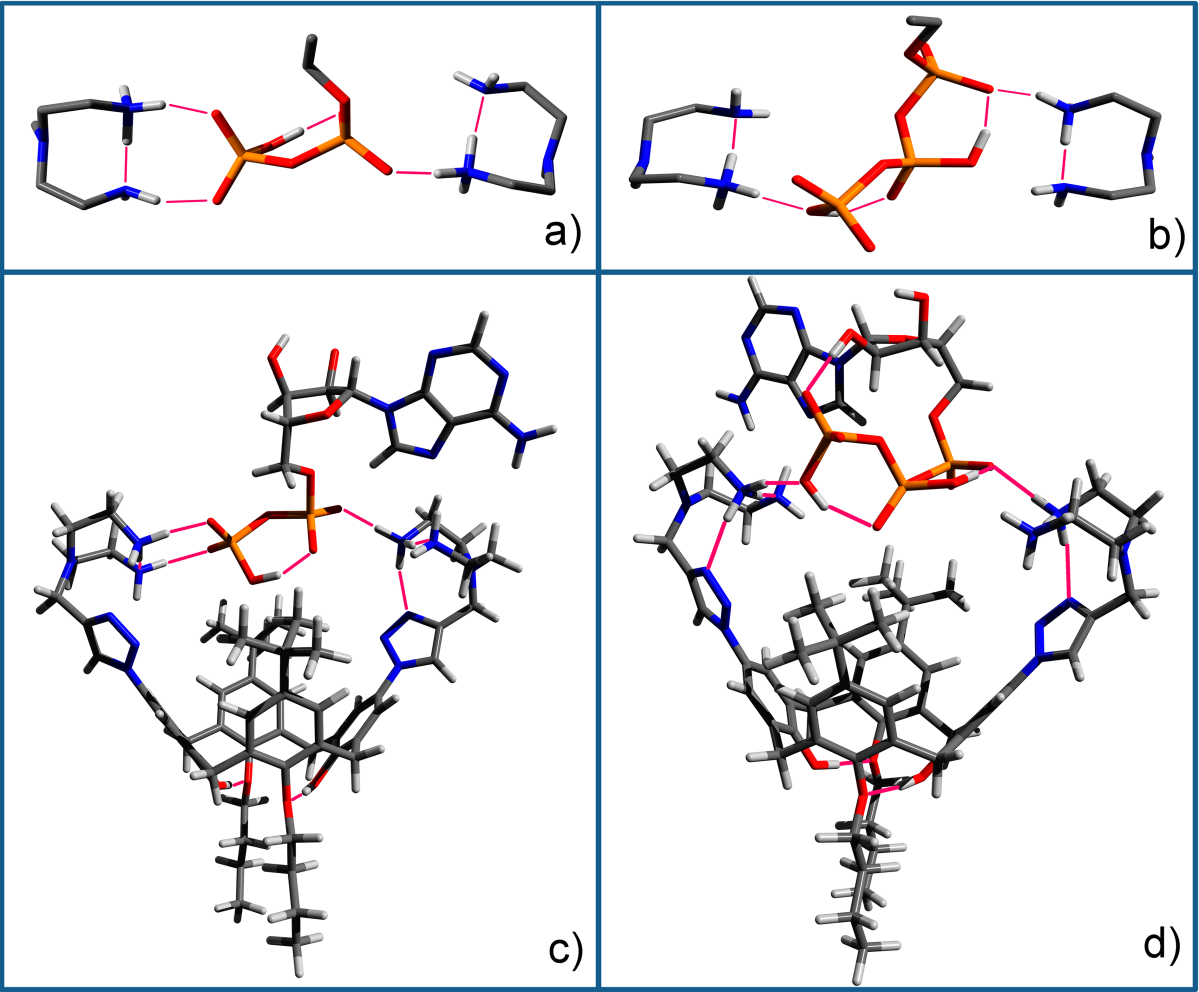
Supramolecular binding motif of diphosphate (a) and triphosphate (b) groups of nucleotides with the protonated diethylenetriamine substituents of the calixarene (ribose and adenine fragments are omitted for clarity) and optimized complex structure of **10a** with ADP (c) and ATP (d) according to DFT calculations.

To estimate the ATP/ADP binding selectivity with the positively charged macrocycles a quantum chemical study of the **10a** complexes with ATP and ADP has been carried out. For calculations, the Gaussian 09 program with the DFT/B3LYP method based on the 6-311g basis set with CPCM solvation model with water as the solvent was used [47]. The energy of complex formation was calculated as a difference between free energy of calix[4]arene + ADP/ATP complex and isolated calix[4]arene and ADP/ATP dianion. The complex structures corresponding to the minimum energy as well as supramolecular binding motif of ADP and ATP are presented in Figure 4. As can be seen ADP more effectively embeds into the molecular cleft formed by two ammonium moieties due to a good host–guest geometric size and/or shape complementarity (Figure 4a and c). The three phosphate groups of ATP have a larger size and cannot realize a similar supramolecular motif in the complex. For this reason the triphosphate fragment is rotated by ≈60° around the calixarene axis relative to the diphosphate position (Figure 4b and d). This leads to a weakening of the host–guest binding and to a decrease of ATP complex energy by 1.4 kcal/mol compared to ADP. So the ADP/ATP optical responses for macrocycles **10a**, **12a**,**b** (Figure 3) can be rationalized in terms of the binding properties of the investigated nucleotides. Moreover, the presence of intramolecular hydrogen bonding (Figure 4a and b) can explain the protonation of only one nitrogen atom despite the close protonation constants of both primary amino groups in the ethylenediamine fragment (p*K*_2_ = 9.08 and p*K*_3_ = 9.97 for the diethylenetriamine conjugated acid [48]). Thus, stabilization of the monocationic form prevents the formation of a dicationic species.

**Figure 5:**
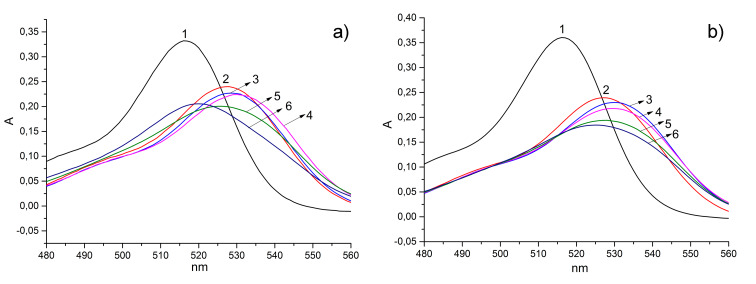
UV spectra of EY (1), **10b**–EY (2), and **10b**–EY in the presence of 0.005 (3), 0.05 (4), 0.5 (5) and 2 (6) mM of ADP (a) or ATP (b). Concentration (EY) = 5 µM, concentration (**10b**) = 6 µM, concentration (MES) = 50 mM (pH 6.5).

The premicellar aggregates of the lipophilic dicationic macrocycle **10b** demonstrate a significant difference of optical response on the presence of ADP and ATP in the solution. Generally, there are two pathways of dye release upon decomplexation: (i) into the buffer solution or (ii) into the aggregate lipophilic domain. However, predominance of each way depends on many factors, such as packaging density of aggregates and their general lipophilicity. To reveal this, absorbance spectra of the **10b**–EY system in the presence of ADP and ATP were recorded (Figure 5a and b).

The addition of small amounts of nucleotides leads to a bathochromic shift of the EY adsorption due to a deeper penetration of EY molecules into the hydrophobic domain of the aggregates and a corresponding decrease of the environment’s polarity (curve 4, Figure 5a). Thus, after decomplexation the EY molecules primarily concentrate inside the aggregate. A hypsochromic shift in the EY spectra pointing to the dye’s release from the aggregates into the solution (curve 6, Figure 5a) is observed at excess concentrations of the nucleotides. However, ADP and ATP cause different changes in the EY spectra. Excess ADP causes release of the dye from the aggregates and appearance of free EY absorption at 516 nm (curve 6, Figure 5a) whereas the dye remains inside the aggregates in the presence of excess ATP (Figure 5b). It is important to note that no EY relocation into the aggregate hydrophobic domain is observed for the less lipophilic calixarene **10a** (Supporting Information File 1, Figure S2a and b).

The interaction of the dicationic calixarene **10b** with ADP results in an uncharged complex, which can be referred to a non-ionic surfactant. At the same time, binding of **10b** with ATP affords a negatively charged complex (Table 2, entries 9 and 10). Obviously the formation of the uncharged complex of **10b** with ADP increases the density of the aggregates through a reduction of repulsion between headgroups [43] thus leading to a dye release from the hydrophobic domain of the aggregates into the buffer solution. Whereas the binding with ATP does not greatly affect the aggregate packing and the dye remains in the hydrophobic core of the aggregate. Therefore, a different packing of aggregates is the main reason for the observed ADP/ATP selectivity of calixarene **10b**.

### Polydiacetylene–**10b** vesicles for ADP/ATP sensing

**Scheme 4:**
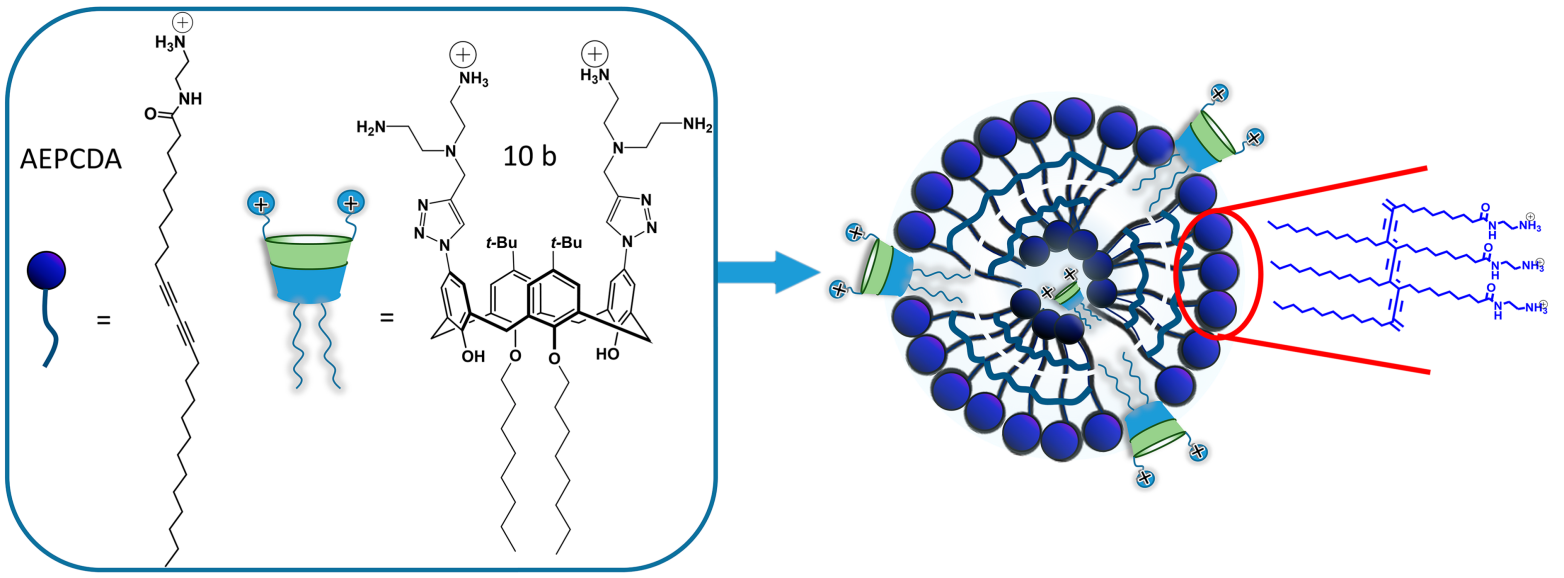
Structure of AEPDA and the corresponding AEPCDA–**10b** polydiacetylene vesicle.

Polydiacetylenes (PDAs), alternating ene-yne conjugated molecules, induce a blue-to-red color transition by the distortion of their backbone. They can be easily prepared through photopolymerization of supramolecularly assembled diacetylenes [49]. The functionalization of a PDA matrix with appropriate receptor fragments offers the possibility to sense various analytes by naked-eye detection. Noncovalent doping of diacetylene surfactant matrix by amphiphilic receptors with subsequent photopolymerization is a commonly used approach for the design of colorimetric analytical devices [50]. Taking into account the cationic nature of the synthesized calixarene macrocycles, the amido-diacetylene lipid bearing a terminal amino group *N*-(2-aminoethyl)pentacosa-10,12-diynamide (AEPCDA) was applied for the formation of the PDA matrix (Scheme 4). The synthesis of AEPCDA was carried out from 10,12-pentacosadiynoic acid according to the literature method [51].

**Figure 6:**
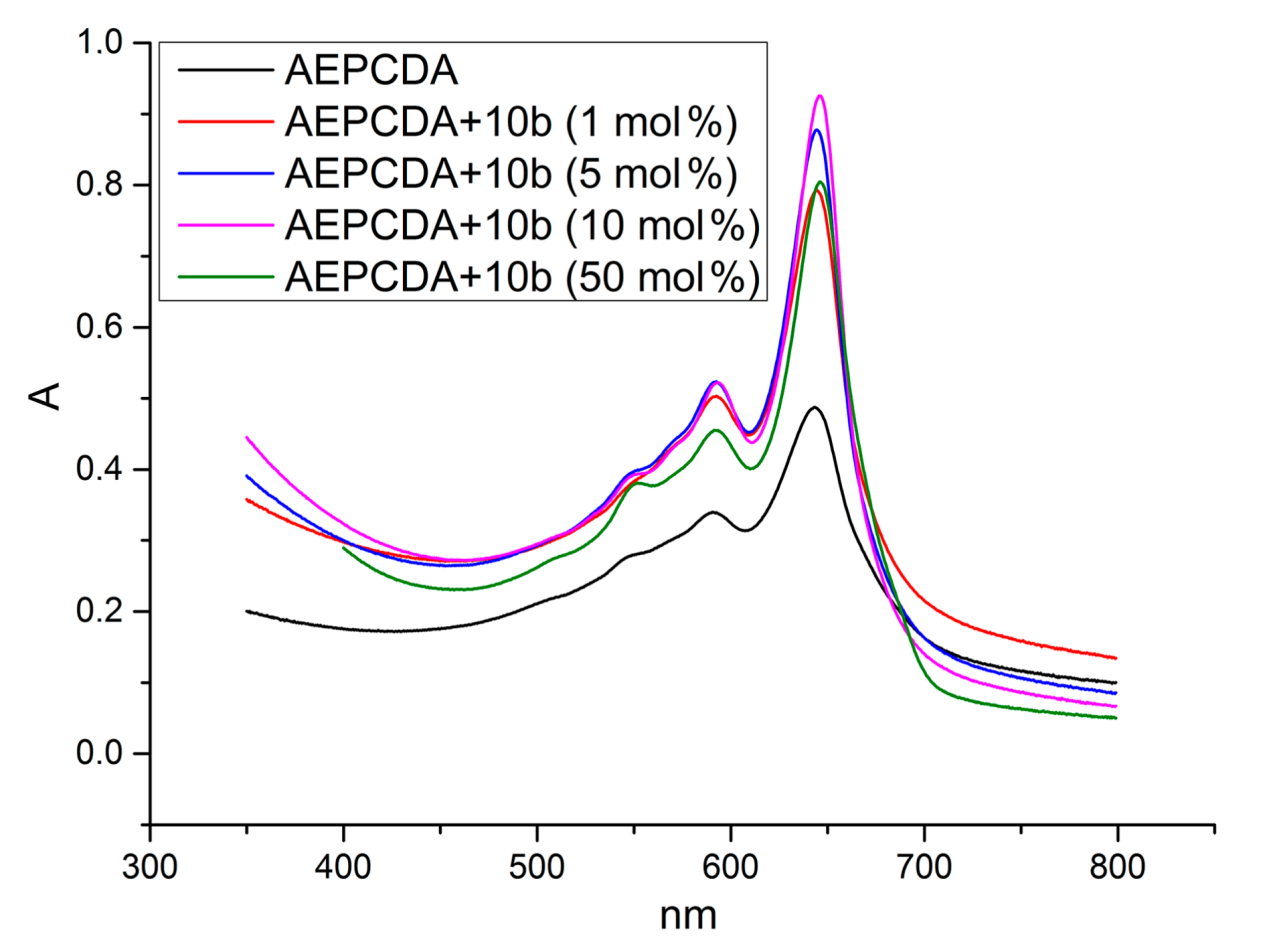
UV spectra of the AEPCDA polydiacetylene vesicles in the presence of different amounts of **10b**; concentration (AEPCDA) = 0.2 mM, concentration (**10b**) = 0.002–0.1 mM in 10 mM MES buffer, pH 6.5.

AEPCDA vesicles containing calixarene **10b** were produced by the well-known film hydration method. To identify an optimal calixarene–AEPCDA ratio a series of PDA vesicles containing 1, 5, 10, 50 mol % of **10b** was prepared. Polymerization of AEPCDA–calixarene vesicles was performed under UV irradiation using 254 nm light in quartz cuvettes of 1 cm path length. The absorbance peak at 674 nm, corresponding to the blue form of the polymer, reached its maximum after 15 minutes of irradiation. It was found that the calixarene **10b** itself acted as stabilizer for AEPCDA vesicles: a hyperchromic effect upon the addition of calixarene up to 50 mol % was observed (Figure 6).

This means that the addition of the non-polymerizable calixarene **10b** to AEPCDA results in an increase of the diacetylene photopolymerization degree. This effect is quite unusual and can be associated with the change of the vesicles packing upon the addition of **10b** and the formation of higher organized nanostructures. DLS data confirmed this suggestion. In the presence of **10b** the size of polydiacetylene vesicles sharply decreases (Table 4 and Supporting Information File 1, Figure S3). This leads to an optimal distance between the diacetylene fragments resulting in a more effective photopolymerization.

**Table 4:** DLS and ELS data for AEPCDA–**10b** polydiacetylene vesicles in the presence/absence of ADP/ATP.^a^

system	*d*, nm	PDI	ζ, mV

AEPCDA	402 ± 109	0.530 ± 0.08	+45 ± 6
AEPCDA + **10b**	94 ± 1	0.175 ± 0.01	+47 ± 4

^a^All measurements were carried out in 50 mM MES buffer at pH 6.5; concentration (AEPCDA) = 0.2 mM, concentration (**10b**) = 0.1 mM, concentration (nucleotide) = 0.5 mM in 10 mM MES buffer, pH 6.5.

The obtained AEPCDA vesicles containing 50 mol % of **10b** were used for the colorimetric recognition of ADP and ATP. The values of colorimetric response (CR) of the AEPCDA–**10b** vesicles were calculated according to Equation 2 [52], which characterize the conversion to the red phase in percent:

[2]
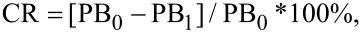


where PB_1_ and PB_0_ is the percent of the blue form in the presence and the absence of the analyte and is defined as follows (Equation 3):

[3]
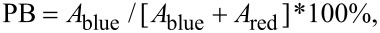


where *A*_blue_ is the absorbance at 640 nm and *A*_red_ is the absorbance at 540 nm.

**Figure 7:**
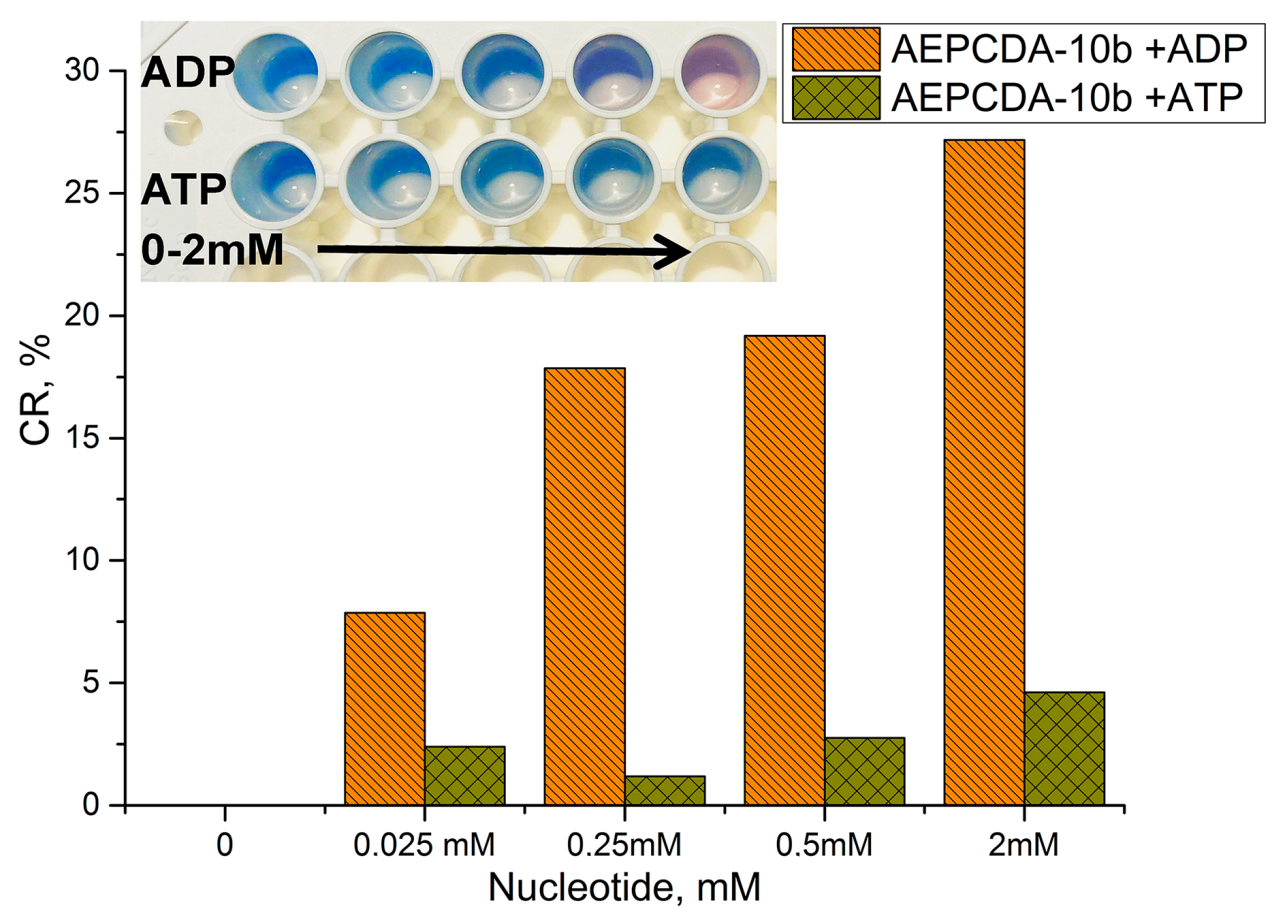
Photographs of a portion of a 96-well plate containing AEPCDA–**10b** polydiacetylene vesicles in the absence (first hole from left) or presence of ADP or ATP; concentration (AEPCDA) = 0.2 mM, concentration (**10b**) = 0.1 mM (a) concentration (nucleotide) = 0.025, 0.25, 0.5, 2 mM in 10 mM MES buffer, pH 6.5.

According to the obtained data (Figure 7) AEPCDA–**10b** vesicles exhibit a colorimetric response toward ADP starting at 0.25 mM of the latter, while there is no response to ATP in this concentration range. Moreover, the response toward ADP can be detected with the naked eye beginning at ADP concentrations as low as 0.5 mM.

Thus, the mechanism of the colorimetric response of **10b**-decorated AEPCDA polydiacetylene vesicles upon binding to ADP can be a result of a complexation-induced distortion of the calixarene cavity provoking thus perturbation of the PDA backbone.

## Conclusion

For the first time new calix[4]arenes adopting a *cone* stereoisomeric form bearing two or four azide groups directly located at the macrocycles’ aromatic rings have been synthesized and used for the preparation of water-soluble triazolyl amphiphilic receptors with two or four polyammonium headgroups by CuAAC reaction with 3-bis[2-(*tert*-butoxycarbonylamino)ethyl]propargylamine. These macrocycles form stable aggregates in aqueous solutions with average hydrodynamic diameters of 150–200 nm and electrokinetic potentials about +40 to +60 mV. CAC values measured by the dye micellization method with pyrene and eosin Y (EY) as dye probes were used to identify optimal concentration conditions for the design of supramolecular architectures for the recognition and sensing based on the principle of competitive EY displacement by adenine-containing nucleotides. The incorporation of **10b** into a polydiacetylene matrix allowed to create covalently bonded vesicles for the selective detection of ADP.

## Experimental

### Material and methods

All reagents were purchased from either Acros or Sigma-Aldrich and were used without further purification. Solvents were purified according to standard methods [53]. 3-Bis[2-(*tert*-butoxycarbonylamino)ethyl]propargylamine [54], **1a** (5,11,17,23-tetra-*tert-*butyl-25,27-dibutoxy-26,28-dihydroxycalix[4]arene) [32]; **2a** (5,17-di-*tert*-butyl-11,23-dinitro-25,27-dibutoxy-26,28-dihydroxycalix[4]arene) [33], **3a** (5,17-di-*tert*-butyl-11,23-diamino-25,27-dibutoxy-26,28-dihydroxycalix[4]arene) [33], **1b** (5,11,17,23-tetra-*tert*-butyl-25,27-dioctyloxy-26,28-dihydroxycalix[4]arene) [34], **2b** (5,17-di-*tert*-butyl-11,23-dinitro-25,27-dioctyloxy-26,28-dihydroxycalix[4]arene) [34], as well as **5a** (5,11,17,23-tetra-*tert-*butyl-25,26,27,28-tetrabutoxycalix[4]arene) [36], **6a** (5,11,17,23-tetranitro-25,26,27,28-tetrabutoxycalix[4]arene) [36], **7a** (5,11,17,23-tetraamino-25,26,27,28-tetrabutoxycalix[4]arene) [35] and **5b** (5,11,17,23-tetra*-tert*-butyl-25,26,27,28-tetraoctyloxycalix[4]arene) [35], **6b** (5,11,17,23-tetranitro-25,26,27,28-tetraoctyloxycalix[4]arene) [35], **7b** (5,11,17,23-tetraamino-25,26,27,28-tetraoctyloxycalix[4]arene) [36] and *N*-(2-aminoethyl)pentacosa-10,12-diynamide [50] were prepared following literature procedures. TLC was performed on Merck UV 254 plates with Vilber Lourmat VL-6.LC UV lamp (254 nm) control. Elemental analysis of the synthesized compounds was done on a PerkinElmer PE 2400 СHNS/О Elemental Analyzer. NMR spectra were recorded on a Bruker Avance 400 Nanobay with signals from residual protons of deuterated solvents (CDCl_3_ or DMSO-*d*_6_) as internal standard. MALDI mass spectra were measured on an UltraFlex III TOF/TOF with PNA matrix, laser Nd:YAG, λ = 355 nm. The IR spectra were recorded on a Bruker Vector-22 spectrometer. Samples were prepared as suspension in mineral oil or as thin films, obtained from chloroform solutions dried on the surface of the KBr disc. Melting points were measured using a Stuart SMP10 apparatus.

#### UV–vis absorbance spectra

The UV–vis-spectra were recorded on a Lambda 35 UV/VIS spectrophotometer (Perkin Elmer Instruments) in an optical cell with 1.0 cm light pass at 298 K.

#### Dynamic light scattering (DLS) measurements

Dynamic light scattering (DLS) experiments and zeta-potential measurements (ELS) were carried out on a Zetasizer Nano ZS instrument (Malvern Instruments, USA) with 4 mW 633 nm He–Ne laser light source and a light scattering angle of 173°. The data were treated with DTS software (Dispersion Technology Software 5.00). The solutions were filtered through Millex HV 0.45 μM filter before the measurements to remove dust. The experiments were carried out in the disposable plastic cells DTS 0012 (Sigma-Aldrich, USA) at 298 K with at least three experiments for each system. Statistical data treatment was done using t-Student coefficient and the particle size determination error was <2%. The prepared samples were ultrasonicated for 30 min at 25 °C before measurements.

#### Fluorescence spectroscopy

Fluorescence experiments were performed in 1.0 cm quartz cuvettes and recorded on a Fluorolog FL-221 spectrofluorimeter (HORIBA Jobin Yvon) in the range of 350 to 430 nm at excitation wavelength 335 nm with 2.5 nm slit for the pyrene. All studies were carried out in buffered aqueous solution (MES buffer, pH 6.5) at 298 K.

#### X-ray data

The crystal of **8a** (C_44_H_52_N_12_O_4_, *M* = 812.98) is monoclinic, space group *P*2_1_/*n*, at *T* = 100 K: *a* = 15.003(3) Å, *b* = 19.652(4) Å, *c* = 15.368(3) Å, β = 104.71(3)°, *V* = 4382.6(16) Å^3^, *Z* = 4, *d*_calcd_ = 1.232 g/cm^3^, *F*(000) = 1728, μ = 0.172 mm^−1^. X-ray diffraction data were collected at the ‘Belok’ beamline (λ = 0.96990 Å) of the National Research Center ‘Kurchatov Institute’ (Moscow, Russian Federation) using a Rayonix SX-165 CCD detector. A total of 720 images (33746 reflections, 8078 independent reflections, *R*_int_ = 0.0882) were collected with an oscillation range of 1.0° (φ scan mode, 2θ_max_ = 72.0°) using two different orientations for the crystal. The semiempirical correction for absorption was applied using the *Scala* program (*T*_min_ = 0.950; *T*_max_ = 0.980) [55]. The data were indexed, integrated and scaled using the utility *iMOSFLM* implemented in the CCP4 program [56–57]. The structure was solved by intrinsic phasing modification of direct methods [58] and refined by full-matrix least squares technique on *F*^2^ with anisotropic displacement parameters for non-hydrogen atoms. One of the four *n*-butyl groups is disordered over two sites with the occupancies of 0.6:0.4. The hydrogen atoms were placed in calculated positions and refined within riding model with fixed isotropic displacement parameters [*U*_iso_(H) = 1.5*U*_eq_(C) for the CH_3_-groups and 1.2*U*_eq_(C) for the other groups]. The final divergence factors were *R*_1_ = 0.0949 for 5301 independent reflections with *I* > 2*σ*(*I*) and *wR*_2_ = 0.2482 for all independent reflections, *S* = 0.941. The calculations were carried out using the SHELXTL program [59]. Crystallographic data for **1** have been deposited with the Cambridge Crystallographic Data Center, CCDC 1831063. Copies of this information may be obtained free of charge from the Director, CCDC, 12 Union Road, Cambridge CB2 1EZ, UK (fax: +44 1223 336033; e-mail: deposit@ccdc.cam.ac.uk or http://www.ccdc.cam.ac.uk).

#### Vesicle preparation and polymerization procedure

In a similar manner as described before [22], concentrated dichloromethane solutions of *N*-(2-aminoethyl)pentacosa-10,12-diynamide (AEPCDA) and the appropriate amounts of calixarene were mixed together and the organic solvent was removed by expulsion with N_2_ at room temperature to give a thin lipid film on the glass surface, which was dried under reduced pressure (0.01 Torr) for 2 h to remove all traces of organic solvent. Then a buffer solution (MES, 50 mM, pH 6.5) was added. The samples were then sonicated for 2 h at 60 °C. The resulting vesicle solution was filtered through a 1.2 µm filter and kept at 4 °C for 12 h. Polymerization was carried out by irradiating the solutions with 254 nm UV light (1 mW/cm^2^) for 15 min under vigorous stirring in 10 mm quartz cuvettes, placed in a thermostat holder at 25 °C.

#### Quantum chemical calculations

Quantum chemical calculations were done in several steps: selection of optimal conformers; organization of initial complex geometry; complex optimization procedure. The conformers search procedure has been done only for calix[4]arene’s triazolyl substituents due to complexity of the whole molecule. Conformers of calix[4]arene substituents and ATP/ADP were generated with cxcalc plug-in (ChemAxon, ChemAxon Jchem https://chemaxon.com/download/jchem-suite) using MMF94 force field, followed by the duplicates discard using in house tool and geometry optimization with PM7 [60] semi-empirical method implemented in the MOPAC 2016 program [61] with discard of repeated duplicates. Then unique conformers were combined with calix[4]arene core, and corresponding cations and complexes with ATP/ADP were optimized by DFT calculations. For DFT calculations Priroda 16 program [62] with build-in PBE functional on L2 basis level [63] was used. Then optimized structures were calculated using DFT calculations with CPCM model of solvation in water with Gaussian 09 program [47] with B3LYP functional on 6-311g basis level.

## Supporting Information

File 1 Synthetic procedures, characterization data and copies of spectra.
