# Supplementary material for: Synthesis of new p-tert-butylcalix[4]arene-based polyammonium triazolyl amphiphiles and their binding with nucleoside phosphates
Source: Beilstein J Org Chem. 2018 Jul 31;14:1980–93. doi: 10.3762/bjoc.14.173 (PMC6122204; doi:10.3762/bjoc.14.173)
Supplement: File 1 — Synthetic procedures, characterization data and copies of spectra. [file Beilstein_J_Org_Chem-14-1980-s001.pdf]

Supporting Information

for

Synthesis of new *p-tert*-butylcalix[4]arene-based polyammonium triazolyl amphiphiles and their binding with nucleoside phosphates

Vladimir A. Burilov[*1], Guzaliya A. Fatikhova[1], Mariya N. Dokuchaeva[1], Ramil I. Nugmanov[1], Diana A. Mironova[1], Pavel V. Dorovatovskii[2], Victor N. Khrustalev[2,3], Svetlana E. Solovieva[1,4] and Igor S. Antipin[1,4].

Address: [1]Kazan Federal University, 18 Kremlevskaya st. Kazan, 420008, Russian Federation, [2]National Research Center “Kurchatov Institute”, 1 Ak. Kurchatov Square, Moscow, 123182, Russian Federation, [3]Peoples’ Friendship University of Russia (RUDN University), 6 Miklukho-Maklay Street, Moscow 117198, Russian Federation and [4]A. E. Arbuzov Institute of Organic & Physical Chemistry, 8 Arbuzov Street, Kazan, 420088, Russian Federation

Email: Vladimir A. Burilov - ultrav@bk.ru

*Corresponding author

Synthetic procedures, characterization data and copies of spectra

General synthetic procedure

Synthesis of compounds **4a,b**

Compound **3a** or**3 b** (1 mmol) was dissolved in DMF/glacial acetic acid (3:10 v/v, 13 ml). Then, the mixture was cooled to 0 °C and 0.14 g of sodium nitrite (2.1 mmol), dissolved in cold water (4 ml), was added dropwise under stirring. The mixture was stirred for 1 h and 0.26 g of sodium azide (4 mmol) in 18 ml water was added. Gas evolution was observed during this stage. The solution was stirred for another 4 h, then, an aqueous solution of $NaHCO_3$ was added to adjust pH up to neutral. The product was extracted with CH_2Cl_2 (2 × 30 ml) and the combined organic phase was dried over $MgSO_4$. The crude product was washed with methanol (2 × 20 ml) to give products **4a**,**b** as light red powders.

4a (5,17-Di-*tert*-butyl-11,23-diazide-25,27-dibutoxy-26,28-dihydroxycalix[4]arene): (0.75 g, 97%); mp**:** decomp. **>**119°C; R_f (TLC, hexane-EtOAc 7:1) = 0.44; ^{1}H-NMR (400 MHz, $CDCl_3$) δ 8.37 (s, Ar-OH, 2H,), 6.96 (s, ArH, 4H), 6.70 (s, ArH, 4H), 4.31 (d, *J* =12.7 Hz, $ArCH_2Ar$, 4H), 3.99 (t, *J* = 7.0 Hz, -O-CH_2-, 4H), 3.32 (d, *J* = 12.8 Hz, $ArCH_2Ar$, 4H), 2.07 (m, -CH_2-, 4H), 1.70 (m, -CH_2-, 4H), 1.05-1.14 (m, -CH_3, -$C(CH_3)_3$, 24H); ^{13}C-NMR (101 MHz, $CDCl_3$) δ 150.45, 150.34, 147.92, 145.53, 132.81, 130.47, 130.38, 125.97, 125.72, 118.76, 116.21, 34.35, 32.26, 31.86, 31.47, 31.32, 19.46, 14.24; MALDI-TOF *m/z*: 678 $[M-2N_2+4H]^+$, 1354 $[2M-4N_2+6H]^+$; FTIR (neat, cm^{-1}) 1253 ($ν_{as}$ Ar-O-Alk), 1362 (δ–C-CH_3), 1478 (δ –CH_2-), 2109 ($ν_{as}$ N_3), 2871 ($ν_s$ –CH_3), 2934 ($ν_{as}$ –CH_2-), 2960 ($ν_{as}$ -CH_3), 3300 (ν OH); calcd for $C_{44}H_{54}N_6O_4$ %: C, 72.30, H, 7.45, N, 11.50 found %: C, 72.46, H, 7.59, N, 11.30.

4b (5, 17-di-*tert*-butyl-11,23-diazide-25,27-dioctyloxy-26,28-dihydroxycalix[4]arene): (0.82 g, 98%); mp: decomp. **>**73°C; R_f (TLC, hexane-EtOAc 7:1) = 0.40; ^{1}H-NMR (400 MHz, $CDCl_3$) δ 8.33 (s, -OH, 2H), 6.95 (s, ArH, 4H,), 6.69 (s, ArH, 4H), 4.32 (d, *J* =12.9 Hz, $ArCH_2Ar$, 4H,), 3.98 (t, *J* = 6.7 Hz, -OCH_2-, 4H), 3.31 (d, *J* = 12.8 Hz, $ArCH_2Ar$, 4H), 2.09 (m, -CH_2-,4H), 1.04 - 1.61 (m, -CH_2-, -$C(CH_3)_3$, 38H,), 0.89 (t, *J* = 6.4 Hz, -CH_3, 6H); ^{13}C-NMR (101 MHz, $CDCl_3$) δ 150.41, 147.91, 132.87, 130.47, 125.96, 118.74, 34.35, 32.08, 31.89, 31.39, 30.08, 29.67, 29.51, 26.05, 22.83, 14.28; MALDI-TOF *m/z*: 790 $[M-2N_2+4H]^+$, 813 $[M-2N_2+4H+Na]^+$, 817 $[M-N_2+2H]^+$. FTIR (neat, cm^{-1}) 1254 ($ν_{as}$ Ar-O-Alk), 1478 (δ –CH_2-), 2108 ($ν_{as}$ N_3), 2857 ($ν_s$ -CH_2-), 2928 ($ν_{as}$ -CH_2-), 2965 ($ν_{as}$ -CH_3), 3311 (ν OH); calcd for $C_{52}H_{70}N_6O_4$ %: C, 74.07, H, 8.37, N, 9.97 found %: C, 74.24, H, 8.46, N, 9.91.

Synthesis of compounds **8a,b**

Compound **7a** or **7b** (1 mmol) was dissolved in DMF/glacial acetic acid (3:10 v/v, 13 ml). Then, the mixture was cooled to 0 °C and 0.28 g of sodium nitrite (4.1 mmol), dissolved in cold water (15 ml) was added dropwise under stirring. The mixture was stirred for 1 h and 0.52 g of sodium azide (8 mmol) in 18 ml water was added. Gas evolution was observed during this stage. The solution was stirred for another 4 h, then, an aqueous solution of $NaHCO_3$ was added to adjust pH up to neutral. The product was extracted with CH_2Cl_2 (2 × 30 ml) and the organic phase was dried over $MgSO_4$. The solvent was evaporated in vacuo. The crude product was washed with methanol (2 × 20 ml) to give products **8a**,**b** as beige powders.

8a (5,11,17,23-tetraazide-25,26,27,28-tetrabutoxy-calix[4]arene): (0.76 g, 93%); mp: decomp. >160°C; R_f (TLC, hexane-EtOAc 7:1) = 0.50; ^{1}H-NMR (400 MHz, $CDCl_3$) δ 6.29 (s, ArH, 8H), 4.40 (d, *J* = 13.6 Hz, $ArCH_2Ar$, 4H,), 3.83 (t, *J* = 7.4 Hz, $-OCH_2-$, 8H), 3.10 (d, *J* = 13.7 Hz, $ArCH_2Ar$, 4H), 1.83 (m, $-CH_2-$, 8H), 1.42 (m, $-CH_2-$, 8H), 0.98 (t, *J* = 5.4 Hz, $-CH_3$, 12H,); ^{13}C-NMR (101 MHz, $CDCl_3$) δ 154.10, 136.42, 133.73, 118.67, 75.24, 32.27, 31.25, 19.42, 14.17; MALDI-TOF m/z: 762 $[M-2N_2+6H]^+$, 788 $[M-N_2+4H]^+$, 896 $[M-N_2+2H+PNA]^+$; FTIR (neat, cm^{-1}) 1239 (ν_{as} Ar-O-Alk), 1465 (δ $-CH_2-$), 2110 (ν_{as} N_3), 2871 (ν_{as} $-CH_3$), 2927 (ν_{as} $-CH_2-$), 2958 (ν_{as} $-CH_3$); calcd for $C_{44}H_{52}N_{12}O_4$ %: C, 65.01, H, 6.45, N, 20.68 found %: C, 65.13, H, 6.56, N, 20.50.

8b (5,11,17,23-tetraazide-25,26,27,28-tetraoctyloxy-calix[4]arene): (0.88g, 85%); mp.: 112°C; R_f (TLC, hexane-EtOAc 7:1) = 0.46; ^{1}H-NMR (400 MHz, $CDCl_3$) δ 6.29 (s, ArH, 8H), 4.39 (d, *J* = 13.6 Hz, $ArCH_2Ar$, 4H), 3.81 (t, *J* = 7.4 Hz, $-OCH_2-$, 8H), 3.09 (d, *J* = 13.7 Hz, $ArCH_2Ar$, 4H), 1.83 (m, $-CH_2-$,8H), 1.31 (m, $-CH_2-$, 48H), 0.89 (t, *J* = 6.7 Hz,$-CH_3$, 12H); ^{13}C-NMR (101 MHz, $CDCl_3$) δ 154.13, 136.43, 133.72, 118.66, 75.57, 32.09, 31.28, 30.34, 30.00, 29.73, 26.42, 22.84, 14.24. MALDI-TOF *m/z*: 1033 $[M-N_2+6H+2K+Na]^+$, 1059 $[M-3N_2+2H+Na]^+$; FTIR (neat, cm^{-1}) 1237 (ν_{as} Ar-O-Alk), 1465 (δ $-CH_2-$), 2115 (ν_s N_3), 2853 (ν_s $-CH_2-$), 2924 (ν_{as} $-CH_2-$), 2956 (ν_{as} $-CH_3$); calcd for $C_{60}H_{84}N_{12}O_4$ %: C, 69.47, H, 8.16, N, 16.20 found %: C, 69.56, H, 8.23, N, 16.13.

Synthesis of compounds **9a,b**

1 mmol of **4a** or **4b**, 1.02 g (3 mmol) 3-bis[2-(*tert*-butoxycarbonyl-amino)ethyl]propargylamine, 0.28 g (1.5 mmol) CuI and 12.3 g of triethylamine (120 mmol, ρ = 0,726 g/cm^3) were dissolved in 30 ml toluene and N_2 was bubbled through

the solution. The reaction mixture was stirred at 40 °C for 4 h. The solvent was evaporated in vacuo. The obtained residue was then dissolved in CH_2Cl_2 (80 ml) and washed with a NH_4OH (3 × 30) and water (2 × 40). The organic layer was dried over $MgSO_4$. The solvent was evaporated in vacuo. The crude product was precipitated in CH_2Cl_2/hexane to give products **9a**,**b** as beige powders.

9a (5,17-di-*tert*-butyl-11,23-bis(4-((bis(2-((*tert*- butoxycarbonyl)amino)ethyl) amino)methyl)-*1H*-1,2,3-triazol-1-yl))-25,27-dibutoxy-26,28-dihydroxy-calix[4]arene): (0.88 g, 62%); mp: 113°C; R_f (TLC, $CHCl_3$:MeOH 3:1) = 0.83; ^{1}H-NMR (400 MHz, $CDCl_3$) δ 8.67 (s, ArOH, 2H), 7.79 (s, -CH(Trz)-, 2H), 7.45 (s, ArH, 4H), 6.99 (s, ArH, 4H), 5.04 (brs, -NH-, 4H), 4.36 (d, *J*=13 Hz, $ArCH_2Ar$, 4H), 4.03 (t, *J*=6.3 Hz, $-OCH_2-$, 4H), 3.89 (brs, -Trz-CH_2-N, 4H), 3.45 (d, *J*=13.1 Hz, $ArCH_2Ar$, 4H,), 3.27 (brs, $-CH_2N$, 4H), 2.63 (brs, $-CH_2N$, 4H), 2.08 (m, $-CH_2-$, 4H), 1.75 (m, $-CH_2-$, 4H), 1.44 (s, -$C(CH_3)_3$(Boc-), 36H), 1.07 (s, $-C(CH_3)_3$, 18H), 0.88 (t, *J*=6.4 Гц, $-CH_3$, 6H); ^{13}C-NMR (101 MHz, $CDCl_3$) δ 150.18, 148.23, 132.17, 129.78, 126.21, 120.67, 77.48, 77.16, 76.84, 32.35, 31.85, 31.43, 28.57, 19.53, 14.25; MALDI-TOF *m/z*: 1414 $[M+H]^+$, 1437 $[M+H+Na]^+$. FTIR (neat, cm^{-1}) 1249 (ν_{as} Ar-O-Alk), 1365 (δ $-C(CH_3)_3$), 1391 (δ -$C(CH_3)_3$), 1493 (δ_{as} $–CH_2-$), 1700 (ν –C=O), 2849 (ν_s $-CH_2-$), 2872 (ν_s $-CH_3$), 2931 (ν_{as} $-CH_2-$), 2963 (ν_{as} $-CH_3$), 3008 (ν -NH-), 3326 (ν OH);. calcd for $C_{78}H_{116}N_{12}O_{12}$ %: C, 66.26, H, 8.27, N, 11.89 found %: C, 66.36, H, 8.43, N, 11.63.

9b (5,17-di-*tert*-butyl-11,23-bis(4-((bis(2-((*tert*- butoxycarbonyl)amino)eth-yl)amino)methyl)-*1H*-1,2,3-triazol-1-yl))-25,27-dioctyloxy-26,28-dihydroxy-calix[4]arene): (0.78 g, 51%); mp: 95°C; R_f (TLC, $CHCl_3$:MeOH 3:1) = 0.80; ^{1}H-NMR (400 MHz, $CDCl_3$) δ 8.63 (s, ArOH, 2H), 7.79 (s, -CH(Trz)-, 2H), 7.44 (s, ArH, 4H), 6.98 (s, ArH, 4H), 5.04 (brs, -NH-, 4H), 4.37 (d, *J*=13 Hz, $ArCH_2Ar$, 4H), 4.02 (t, *J*=6.8 Hz, $-OCH_2-$, 4H), 3.88 (brs, Trz-CH_2-N, 4H), 3.45 (4H, d, *J*=13 Hz, $ArCH_2Ar$), 3.25 (brs, $-CH_2N$, 4H), 2.63 (brs, $-CH_2N$, 4H), 2.10 (m, $-CH_2-$, 4H), 1.65 (m, $-CH_2-$, 4H), 1.44 (s, $-C(CH_3)_3$ (Boc-), 36H), 1.33 (m, -CH2-, 16H), 1.07 (s, $-C(CH_3)_3$, 18H), 0.90 (t, *J*=6.3 Hz, $-CH_3$, 6H,); ^{13}C-NMR (101 MHz, $CDCl_3$) δ 156.33, 150.30, 148.18, 132.24, 129.79, 126.18, 120.66, 77.48, 77.16, 76.84, 32.07, 31.86, 31.42, 30.14, 29.65, 29.50, 28.58, 26.09, 22.83, 14.27; MALDI-TOF *m/z*: 1526 $[M+H]^+$, 1548 $[M+Na]^+$; FTIR (neat, cm^{-1}) 1248 (ν_{as} Ar-O-Alk), 1366 (δ $-C(CH_3)_3$), 1391 (δ -$C(CH_3)_3$), 1493 (δ_{as} $–CH_2-$), 1702 (ν –C=O), 2850 (ν_s $-CH_2-$), 2927 (ν_{as} $-CH_2-$), 2960 (ν_{as} $-CH_3$), 3011 (ν -NH-), 3310 (ν OH); calcd for $C_{86}H_{132}N_{12}O_{12}$ %: C, 67.69, H, 8.72, N, 11.01 found %: C, 67.78, H, 8.84, N, 10.96.

Synthesis of compounds **10a,b**

1.0 mmol of **9a** or **9b** was dissolved in 10 ml 1,4-dioxane, then 5 ml (40.0 mmol) of conc. HCl was added dropwise. The reaction mixture was stirred at room temperature for 30 h. The solvent was evaporated in vacuo to give products **10a,b** as beige powders.

10a (5,17-di-*tert*-butyl-11,23-bis(4-((bis(2-(amino)ethyl) amino)methyl)-*1H*-1,2,3-triazol-1-yl))-25,27-dibutoxy-26,28-dihydroxy-calix[4]arene dihydrochloride): (1.0 g, 93%); mp: decomp. >162°C; ^{1}H-NMR (400 MHz, DMSO-d^{6}) δ 9.26 (s, ArOH, 2H), 8.80 (s, CH(Trz), 2H), 8.09 (brs, -NH_2, 8H,), 7.84 (s, ArH, 4H), 7.31(s, ArH, 4H), 4.24 (d, *J*=12.0 Hz, ArCH_2Ar, 4H), 4.17 (brs, -CH2-N, 4H), 4.00 (brs, -OCH2-, 4H), 3.61 (d, *J*=12.0 Hz, ArCH_2Ar, 4H), 3.04 (brs, -CH2-N, 4H), 2.75 (brs, -CH2-N, 4H), 2.02 (brs, -CH_2-, 4H), 1.77 (brs, -CH_2-, 4H), 1.10-1.20 (m, -CH_3, -C$(CH_3)_3$, 24H); ^{13}C-NMR (101 MHz, DMSO-d^{6}) δ 153.01, 149.82, 147.75, 132.33, 129.43, 128.74, 126.32, 120.54, 62.81, 76.47, 49.84, 46.40, 31.81, 31.14, 30.83, 18.87, 14.00; MALDI-TOF *m/z*: 1013 [M-2HCl+H]$^{+}$; FTIR (neat, cm^{-1}) 1242 (ν_{as} Ar-O-Alk), 1362 (δ -C$(CH_3)_3$), 1479 (δ_{as} –CH_2-), 1598 (δ -NH_2), 2870 (ν_s -CH_2-), 2929(ν_s -CH_3), 2934 (ν_{as} -CH_2-), 2956 (ν_{as} -CH_3), 3236 (ν OH); calcd for $C_{58}H_{86}Cl_2N_{12}O_4$ %: C, 64.13, H, 7.98, N, 15.47 found %: C, 64.08, H, 8.08, N, 15.36.

10b (5,17-di-*tert*-butyl-11,23-bis(4-((bis(2-(amino)ethyl) amino)methyl)-*1H*-1,2,3-triazol-1-yl))-25,27-dioctyloxy-26,28-dihydroxy-calix[4]arene dihydrochloride): (1.03 g, 86%); mp: decomp. >158°C; ^{1}H-NMR (400 MHz, DMSO-d^{6}) δ 9.25 (s, ArOH, 2H), 8.83(s, CH(Trz), 2H), 8.17 (brs, -NH_2, 8H,), 7.86 (s, ArH, 4H), 7.31 (s, ArH, 4H), 4.33 (brs, -CH2-N, 4H), 4.24 (d, *J*=12.0 Hz, ArCH_2Ar, 4H), 3.99 (brs, -OCH2-, 4H), 3.61 (d, *J*=12.0 Hz, ArCH_2Ar, 4H), 3.08 (brs, -CH2-N, 4H), 2.82 (brs, -CH2-N, 4H), 2.04 (m, -CH_2-, 4H), 1.70 (m, -CH_2-,4H), 1.46-1.32 (m, -CH_2-, 16H), 1.12 (s, -$(CH_3)_3$, 18H), 0.87 (t, *J*=6.3 Hz, 6H); ^{13}C-NMR (101 MHz, DMSO-d^{6}) δ 153.11, 149.81, 147.77, 132.32, 129.45, 128.67, 126.34, 120.60, 76.62, 72.36, 69.81, 69.58, 62.82, 60.22, 49.63, 46.39, 34.28, 31.44, 31.16, 30.83, 29.62, 29.00, 28.87, 25.44, 22.18,. 14.01; MALDI-TOF *m/z*: 1125 [M-2HCl+H]$^{+}$; FTIR (neat, cm^{-1}) 1290 (ν_{as} Ar-O-Alk), 1362 (δ -C$(CH_3)_3$), 1385 (δ -C$(CH_3)_3$), 1483 (δ_{as} –CH_2-), 1599 (δ -NH_2), 2855 (ν_s -CH_2-), 2924 (ν_{as} –CH_2-), 2953 (ν_{as} –CH_3), 3216 (ν OH); calcd for $C_{66}H_{102}Cl_2N_{12}O_4$ %: C, 66.14, H, 8.58, N, 14.02 found %: C, 66.19, H, 8.65, N, 13.98.

Synthesis of compounds **11a,b**

1 mmol of **8a** or **8b**, 2.04 g (6 mmol) 3-bis[2-(*tert*-butoxycarbonylamino)eth-yl]propargylamine, 0.58 g (3 mmol) CuI and 24.6 g of triethylamine (240 mmol, ρ = 0,726 g/cm^3) were dissolved in 60 ml toluene N_2 was bubbled through the solution. The reaction mixture was stirred at 40 °C for 4 h. The solvent was evaporated in vacuo. The obtained residue was then dissolved in CH_2Cl_2 (80 ml) and washed with a NH_4OH (3 × 30 ml) and water (2 × 40 ml). The organic layer was dried over $MgSO_4$ and the solvent was evaporated in vacuo. The crude product was precipitated in CH_2Cl_2/hexane to give products **11a**,**b** as beige powders.

11a (5,11,17,23-tetra(4-((bis(2-((*tert*-butoxycarbonyl)amino)ethyl)amino)methyl)-*1H*-1,2,3-triazol-1-yl))-25,26,27,28-tetrabutoxycalix[4]arene): (1.82 g, 84%); mp: 88°C; R_f (TLC, $CHCl_3$:MeOH 3:1) = 0.80; ^{1}H-NMR (400 MHz, $CHCl_3$) δ 7.67 (s, CH(Trz), 4H), 7.11 (s, ArH, 8H), 5.27 (brs, -NH-, 8H), 4.58 (d, *J*=13.4 Hz, $ArCH_2Ar$, 4H), 3.99 (brt, -OCH_2-, 8H), 3.81 (brs, Trz-CH_2-NR_2, 8H), 3.33 (d, *J*=13.6 Hz, $ArCH_2Ar$, 4H), -3.22 (brs, -CH_2N, 16H), 2.62 (brs,-CH_2-NH-, 16H), 1.94 (m, -CH_2-, 8H), 1.32-1.52 (m, -CH2-, $C(CH_3)_3$, 80H,), 1.03 (t, *J*=7.4 Hz, -CH_3, 12H,); ^{13}C-NMR (101 MHz, $CDCl_3$) δ 156.75, 156.38, 136.00, 132.03, 121.10, 120.68, 79.09, 75.67, 73.44, 53.25, 52.94, 48.16, 38.43, 32.26, 31.33, 28.59, 19.43, 14.16; MALDI-TOF *m/z*: 2179 $[M+H]^+$; FTIR (neat, cm^{-1}) 1250 (v_{as} Ar-O-Alk), 1365 (δ -$C(CH_3)_3$), 1391 (δ -$C(CH_3)_3$), 1489 ($δ_{as}$ –CH_2-), 1696 (v –C=O), 2873 (v_s -CH_3), 2932 (v_{as} -CH_2-), 2975 (v_{as} -CH_3), 3135 (v -NH-); calcd for $C_{112}H_{176}N_{24}O_{20}$ %: C, 61.74, H, 8.14, N, 15.43 found %: C, 61.83, H, 8.28, N, 15.35.

11b (5,11,17,23-tetra(4-((bis(2-((*tert*-butoxycarbonyl)amino)ethyl)amino)methyl)-*1H*-1,2,3-triazol-1-yl))-25,26,27,28-tetraoctyloxycalix[4]arene): (2.10g, 86%); mp: 86°C; R_f (TLC, $CHCl_3$:MeOH 3:1) = 0.9; ^{1}H-NMR (400 MHz, $CHCl_3$) δ 7.68 (s, -CH (Trz), 4H), 7.11 (s, ArH, 8H), 5.27 (brs,-NH-,8H), 4.57 (d, *J*=13.4 Hz, $ArCH_2Ar$, 4H), 3.97 (m, -OCH_2-, 8H), 3.81 (brs, Trz-CH_2-NR_2, 8H), 3.32 (d, *J*=13.6 Hz, $ArCH_2Ar$, 4H), 3.21 (brs, -CH_2 -N, 16H), 2.55 (m, -CH_2-N, 16H), 1.93 (m, -CH_2-, 8H), 1.24-1.48 (m, -CH_2-, -$C(CH_3)_3$, 112H), 0.89 (t, *J*=6.7 Hz, -CH_3, 12H); ^{13}C-NMR (101 MHz, $CDCl_3$) δ 156.78, 156.39, 136.01, 132.01, 120.70, 79.11, 76.02, 53.25, 38.40, 32.09, 31.36, 30.34, 30.00, 29.71, 28.59, 26.42, 22.83,. 14.23; MALDI-TOF *m/z*: 2404 $[M+2H]^+$; FTIR (neat, cm^{-1}) 1251 (v_{as} Ar-O-Alk), 1365 (δ -$C(CH_3)_3$), 1390 (δ -$C(CH_3)_3$), 1489 ($δ_{as}$ –CH_2-), 1700 (v –C=O), 2855 (v_s –CH_2-), 2927 (v_{as} -CH_2-), 2960 (v_{as} -CH_3), 3136

(ν -NH-); calcd for $C_{128}H_{208}N_{24}O_{20}$ %: C, 63.97, H, 8.72, N, 13.99 found %: C, 64.03, H, 8.81, N, 13.91.

Synthesis of compounds **12a,b**

1.0 mmol of **11a** or **11b** was dissolved in 10 ml 1,4-dioxane, then 10 ml (80.0 mmol) of conc. HCl was added dropwise. The reaction mixture was stirred at room temperature for 30 h. The solvent was evaporated in vacuo to give products **12a**,**b** as beige powders.

12a (5,11,17,23-tetra(4-((bis(2-(amino)ethyl) amino)methyl)-*1H*-1,2,3-triazol-1-yl))-25,26,27,28-tetrabutoxycalix[4]arene tetrahydrochloride): (1.40 g, 92%); mp: 238°C; ^{1}H-NMR (400 MHz, DMSO-d^6) δ 8.81 (s, -CH (Trz), 4H), 8.31 (brs, -NH_2, 16H), 7.40 (s, ArH, 8H), 4.49 (d, *J*=13 Hz, ArCH2Ar, 4H), 4.21 (brs, -CH_2-N, 8H), 3.98 (m, -OCH_2-, 8H), 3.50 (d, *J*=12.2 Hz, ArCH2Ar, 4H), 3.15 (brs, -CH_2-N, 16H), 2.95 (brs, -CH_2-N, 16H), 1.93 (m,-CH_2-, 8H), 1.49 (m, -CH_2-,8H), 1.01 (t, *J*=7.4 Hz, -CH_3, 12H); ^{13}C-NMR (101 MHz, DMSO-d^6) δ 156.30, 154.92, 135.73, 133.44, 131.15, 128.21, 120.15, 116.96, 75.09, 62.82, 50.06, 49.44, 46.42, 31.77, 18.86, 13.92; MALDI-TOF *m/z*: 1378 [M-4HCl+2H]$^+$, 1400 [M-4HCl+H+Na]$^+$. FTIR (neat, cm^{-1}) 1237 (ν_{as} Ar-O-Alk), 1376 (δ_s -CH_3), 1487 (δ_{as} –CH_2-), 1595 (δ -NH_2), 2871 (ν_s -CH_3), 2928 (ν_{as} -CH_2-), 2957 (ν_{as} –CH_3), 3397 (ν_{as} –NH_2); calcd for $C_{72}H_{116}Cl_4N_{24}O_4$ %: C, 56.76, H, 7.67, N, 22.06 found %: C, 56.87, H, 7.76, N, 22.01.

12b (5,11,17,23-tetra(4-((bis(2-(amino)ethyl) amino)methyl)-*1H*-1,2,3-triazol-1-yl))-25,26,27,28-tetraoctyloxycalix[4]arene tetrahydrochloride): (1.60g, 92%); mp: 247°C; ^{1}H-NMR (400 MHz, DMSO-d^6) δ 8.77 (s, CH(Trz), 4H), 8.23 (brs, -NH_2-, 16H), 7.38 (s, ArH, 8H), 4.48 (d, *J*=13 Hz, ArCH2Ar, 4H), 4.21 (brs, -CH_2-N, 8H), 3.83 (m, -OCH_2-, 8H), 3.48 (d, *J*=12.2 Hz, ArCH2Ar, 4H), 3.11 (brs, -CH_2-N, 16H), 2.84 (brs, -CH_2-N, 16H), 1.95 (m, -CH_2-, 8H), 1.22-1.48 (m, -CH_2-, 40H), 0.87 (brs, -CH_3,12H); ^{13}C-NMR (101 MHz, DMSO-d^6) δ 156.54, 135.69, 120.04, 75.40, 62.80, 49.70, 31.54, 29.93, 29.57, 29.20, 25.99, 22.23, 13.93; MALDI-TOF *m/z* 1601 [M-4HCl]$^+$; FTIR (neat, cm^{-1}) 1221 (ν_{as} Ar-O-Alk), 1486 (δ_{as} –CH_2-), 1597 (δ -NH_2), 2852 (ν_s -CH_2-), 2922 (ν_{as} -CH_2-), 2955(ν_{as} -CH_3), 3377 (ν_{as} –NH_2); calcd for $C_{88}H_{148}Cl_4N_{24}O_4$ %: C, 60.43, H, 8.53, N, 19.23 found %: C, 60.37, H, 8.49, N, 19.17.

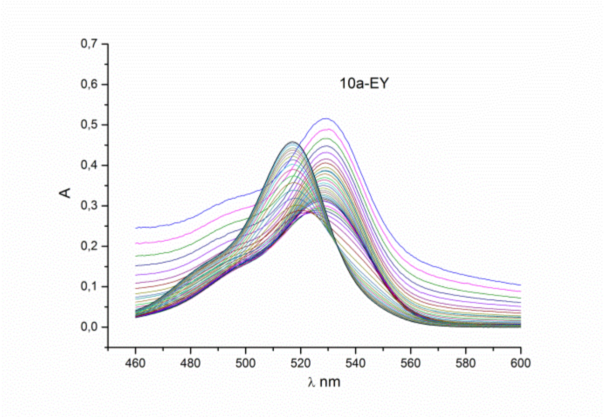

(a)

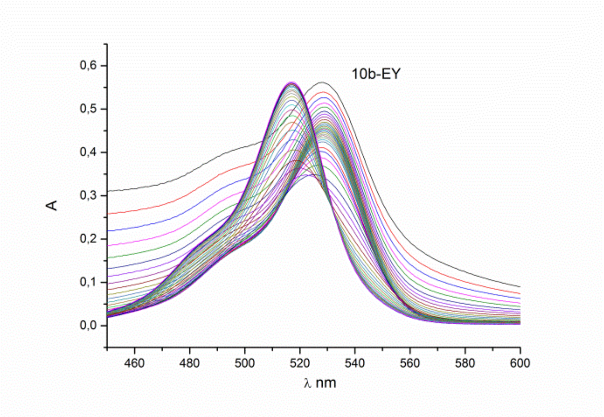

(b)

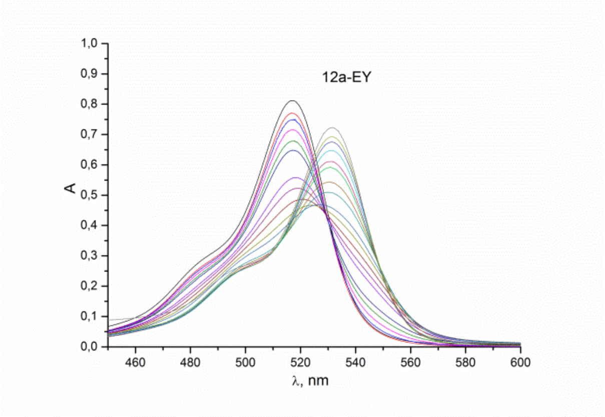

(c)

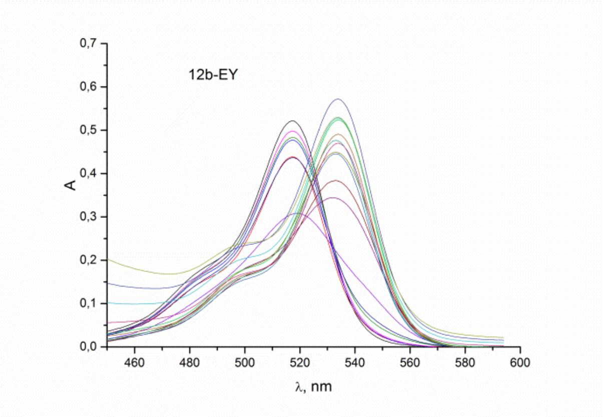

(d)

Figure S1: UV–vis spectra of the EY *vs* calixarene **10a,b** and **12a,b** concentration. C (EY) = 0.005 mM, C (calixarene) = 0,0001 – 1 mM , C (MES) = 50 mM (pH 6.5).

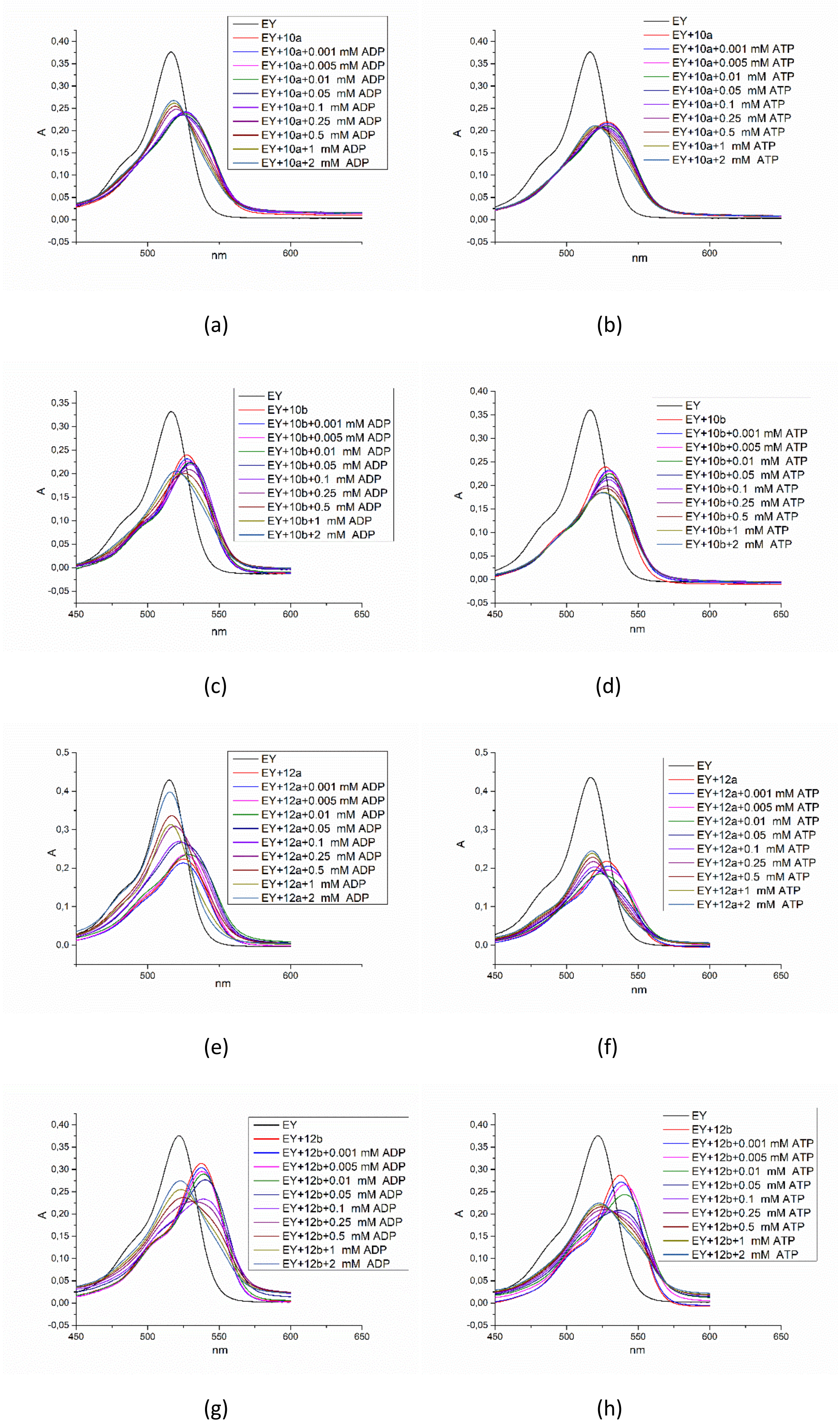

Figure S2: UV spectra of the calixarene-EY systems *vs* adenosine phosphates. C (EY) = 0.005 mM, C (**10a**) = 0.004, C (**10b**) = 0.006 mM, C (**12a**) = 0.004 mM, C (**12b**) = 0.002 mM, C (adenosine phosphate) = 2 mM, C (MES) = 50 mM (pH 6.5).

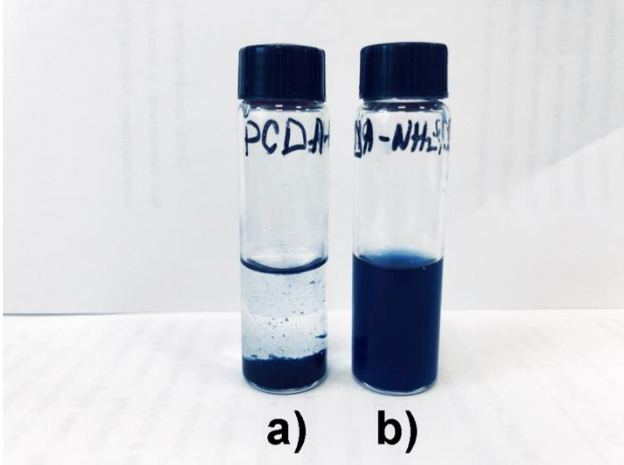

Figure S3: Photography of AEPCDA (a) and AEPCDA**–10b** (b) polydiacetylene vesicles after standing for 2 hours; C(AEPCDA) = 0.2 mM, C (**10b**)= 0.1 mM in 10 mM MES buffer, pH 6.5.

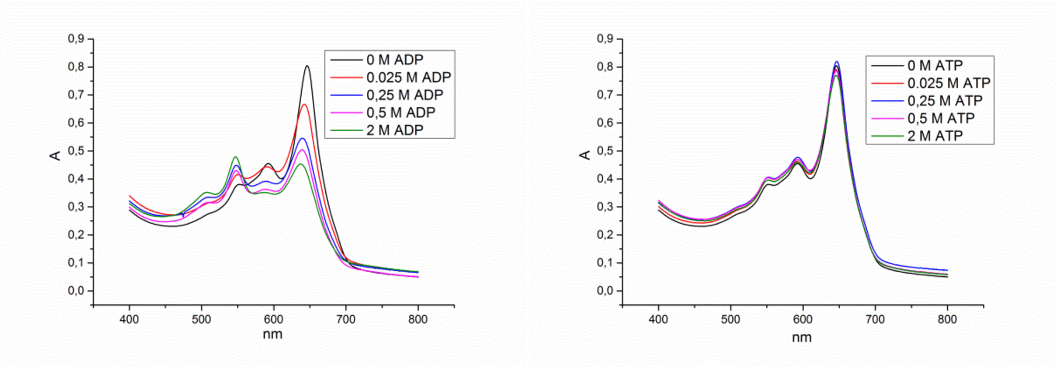

(a) (b)

Figure S4: UV spectra of the AEPCDA–**10b** polydiacetylene vesicles in the absence or presence of ADP(a) or ATP (b); C(AEPCDA) = 0.2 mM, C (10 b)= 0.1 mM (a) C (nucleotide) = 0.025, 0.25, 0.5, 2 mM in 10 mM MES buffer, pH 6.5.

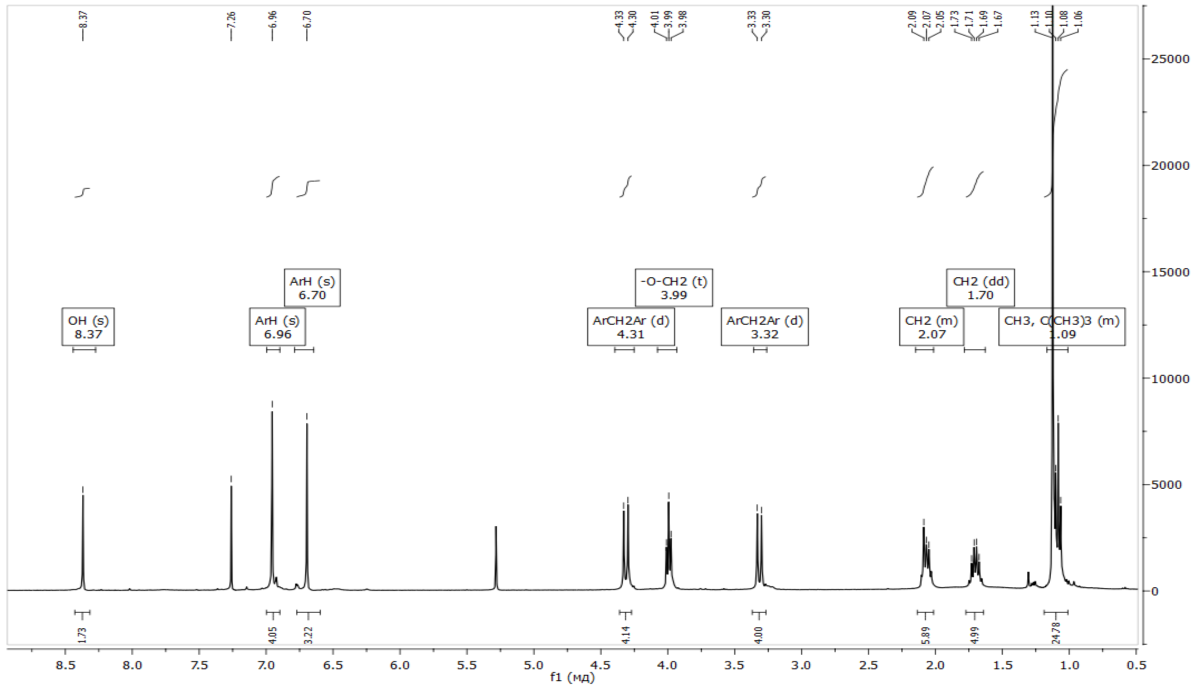

OH (s)
8.37
ArH (s)
6.96
ArH (s)
6.70
ArCH2Ar (d)
4.31
-O-CH2 (t)
3.99
ArCH2Ar (d)
3.32
CH2 (m)
2.07
CH2 (dd)
1.70
CH3, C(CH3)3 (m)
1.09
f1 (мд)

(a)

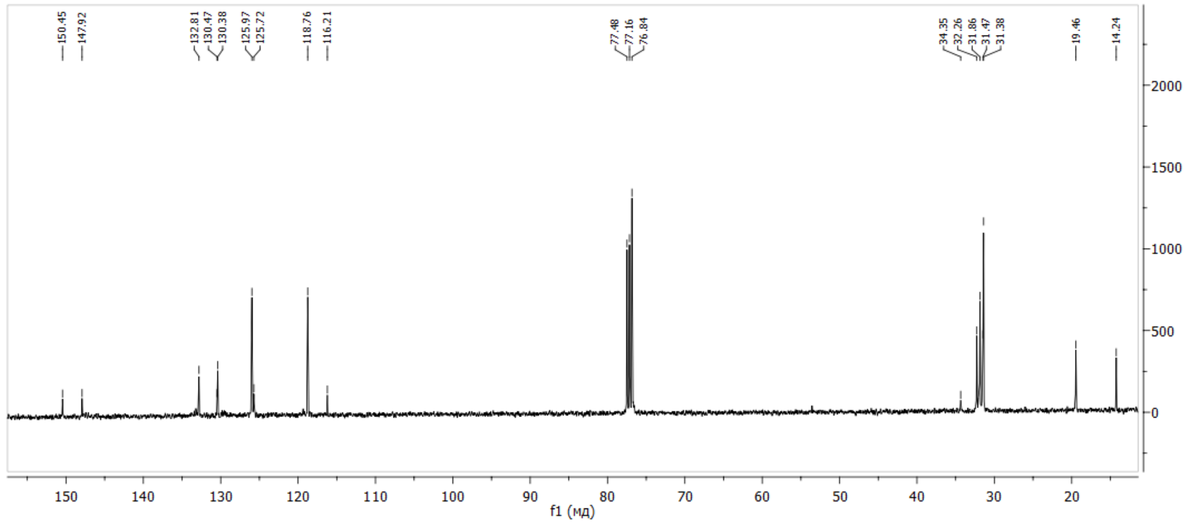

f1 (мд)

(b)

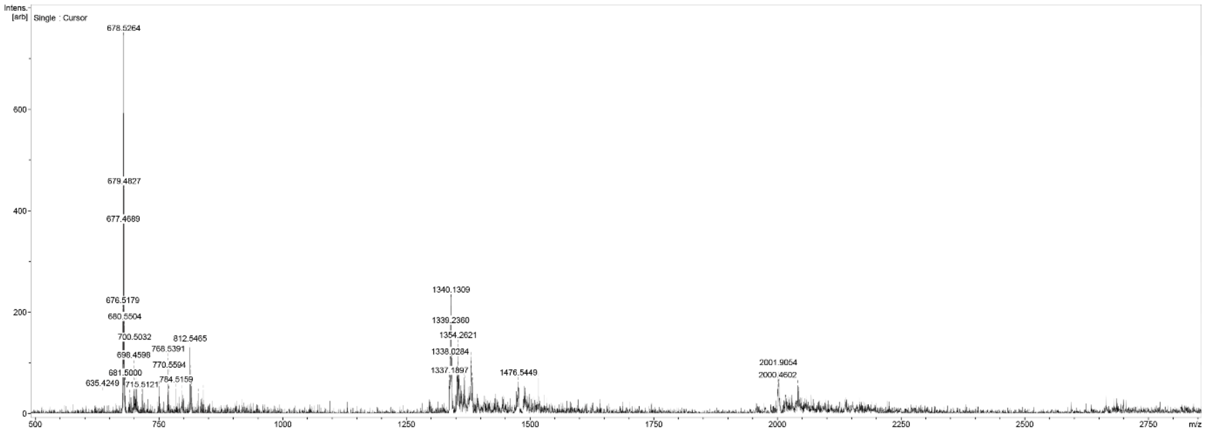

Bruker Daltonics flexControl

Display Screenshot - Generated On 2016-10-13 14h39m56s

(c)

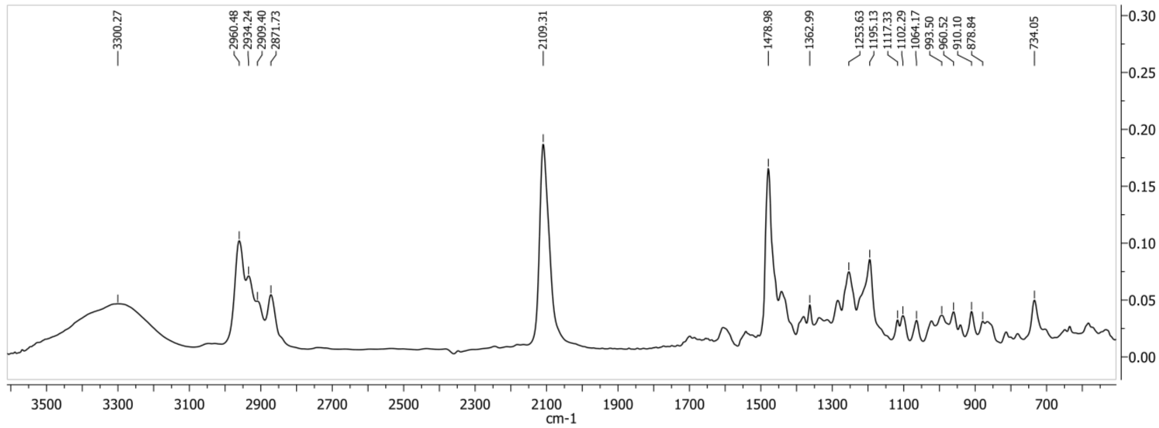

(d)

Figure S5: ^{1}H NMR (a), ^{13}C (b), MALDI TOF (c) and FTIR (d) spectra of **4a** (5, 17-di-*tert*-butyl-11,23-diazide-25,27-dibutoxy-26,28-dihydroxycalix[4]arene).

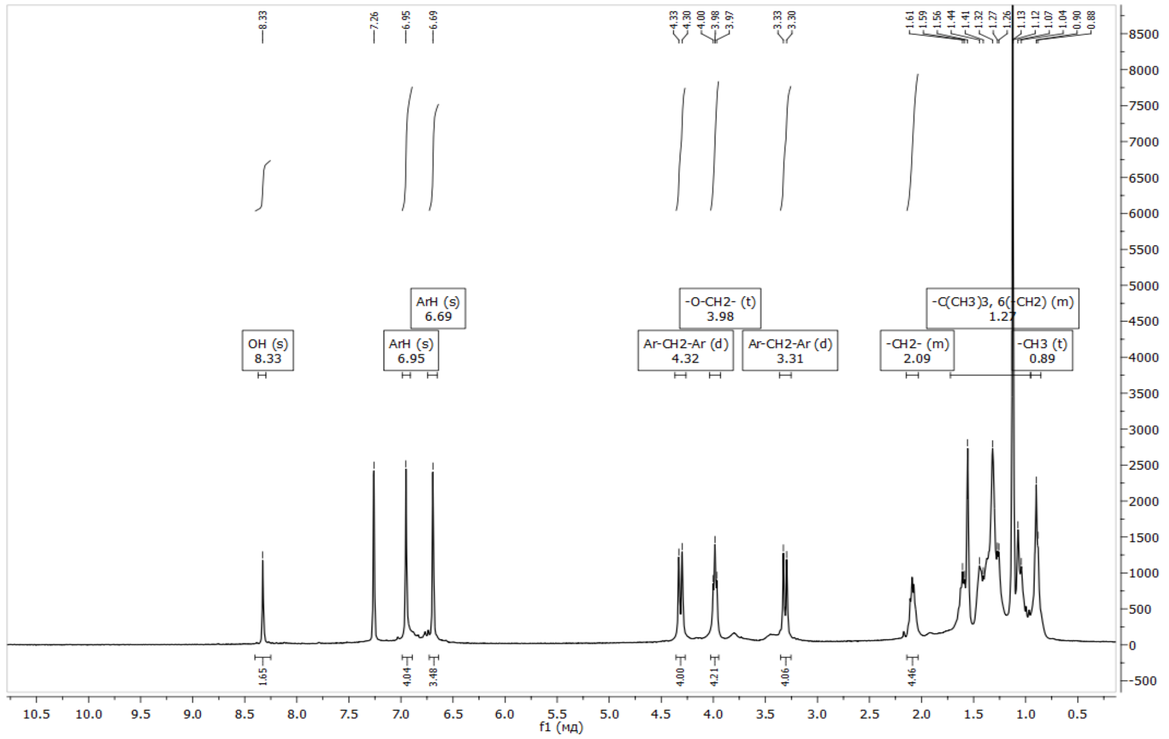

OH (s)
8.33
ArH (s)
6.95
ArH (s)
6.69
-O-CH2- (t)
3.98
Ar-CH2-Ar (d)
4.32
Ar-CH2-Ar (d)
3.31
-CH2- (m)
2.09
-C(CH3)3, 6(-CH2) (m)
1.27
-CH3 (t)
0.89
f1 (мд)

(a)

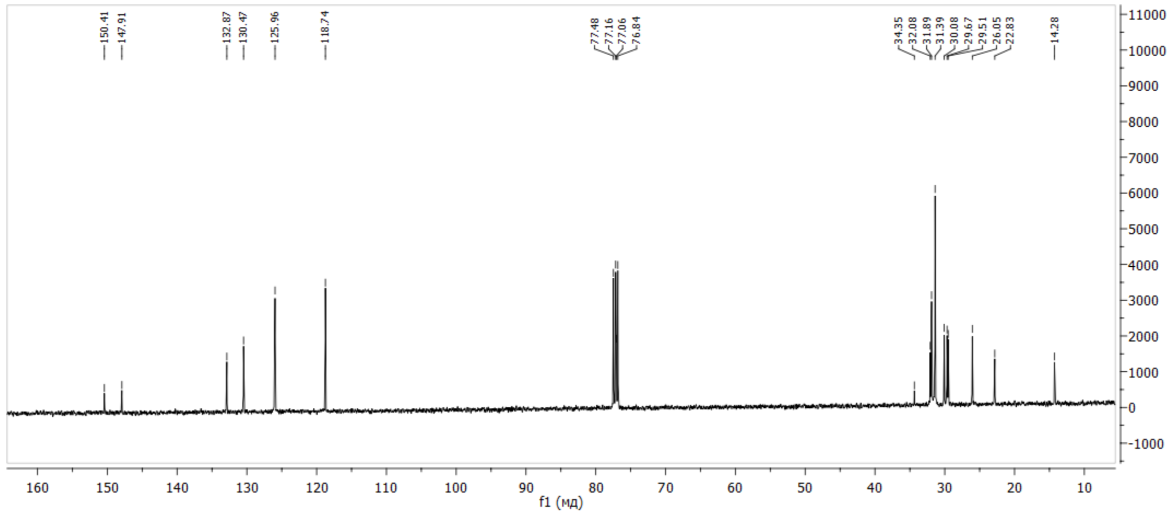

150.41
147.91
132.87
130.47
125.96
118.74
77.48
77.16
77.06
76.84
34.35
32.08
31.89
31.39
30.08
29.67
29.51
26.05
22.83
14.28
f1 (мд)

(b)

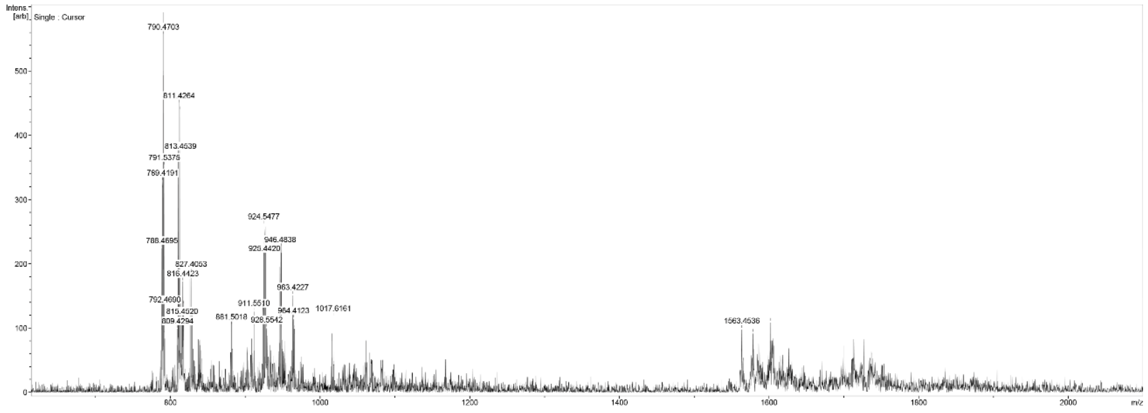

Bruker Daltonics flexControl

Display Screenshot - Generated On 2016-10-13 14h40m46s

(c)

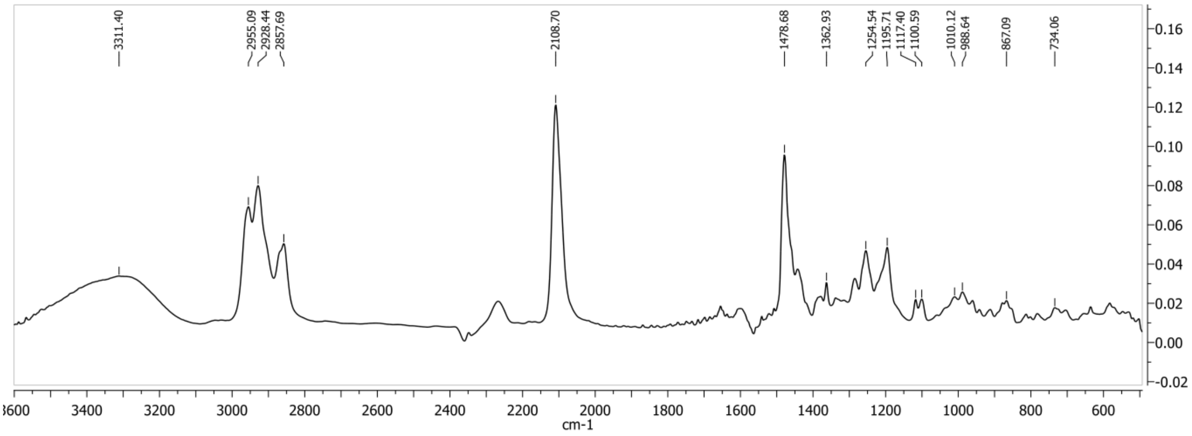

(d)

Figure S6: ^{1}H NMR (a), ^{13}C (b), MALDI TOF (c) and FTIR (d) spectra of **4b** (5,17-di-*tert*-butyl-11,23-diazide-25,27-dioctyloxy-26,28-dihydroxycalix[4]arene).

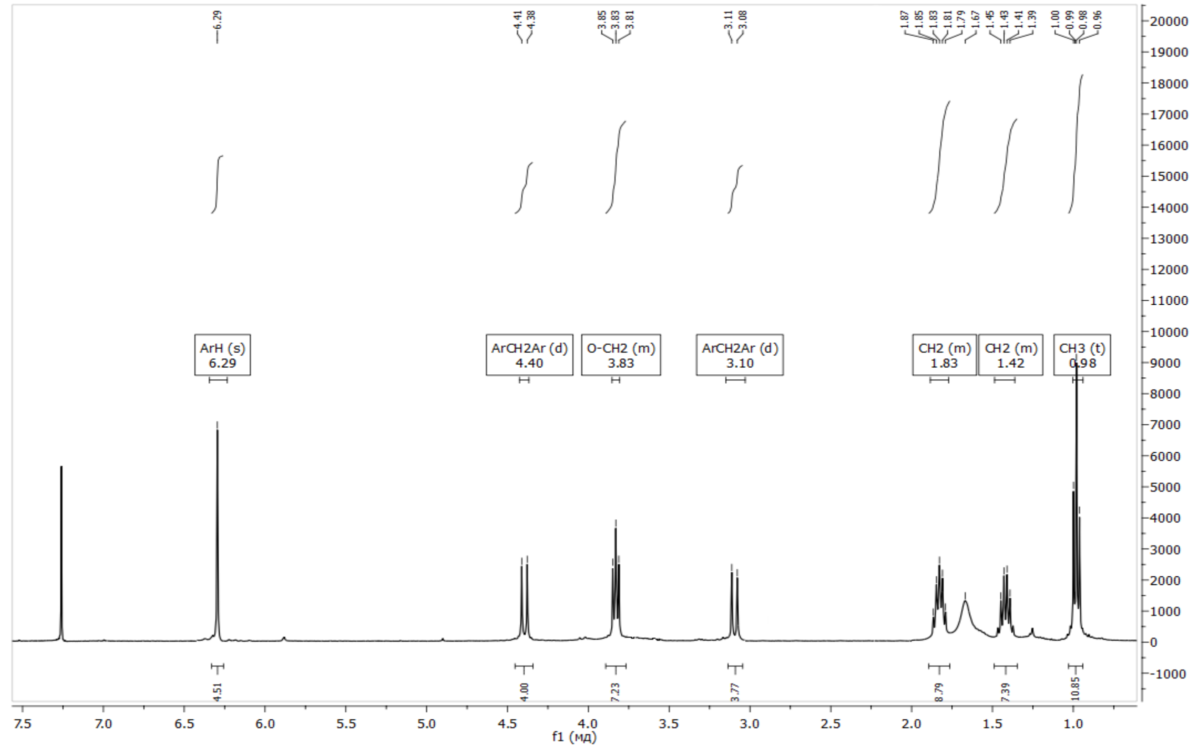

ArH (s)
6.29
ArCH2Ar (d)
4.40
O-CH2 (m)
3.83
ArCH2Ar (d)
3.10
CH2 (m)
1.83
CH2 (m)
1.42
CH3 (t)
0.98
4.51
4.00
7.23
3.77
8.79
7.39
10.85
f1 (мд)

(a)

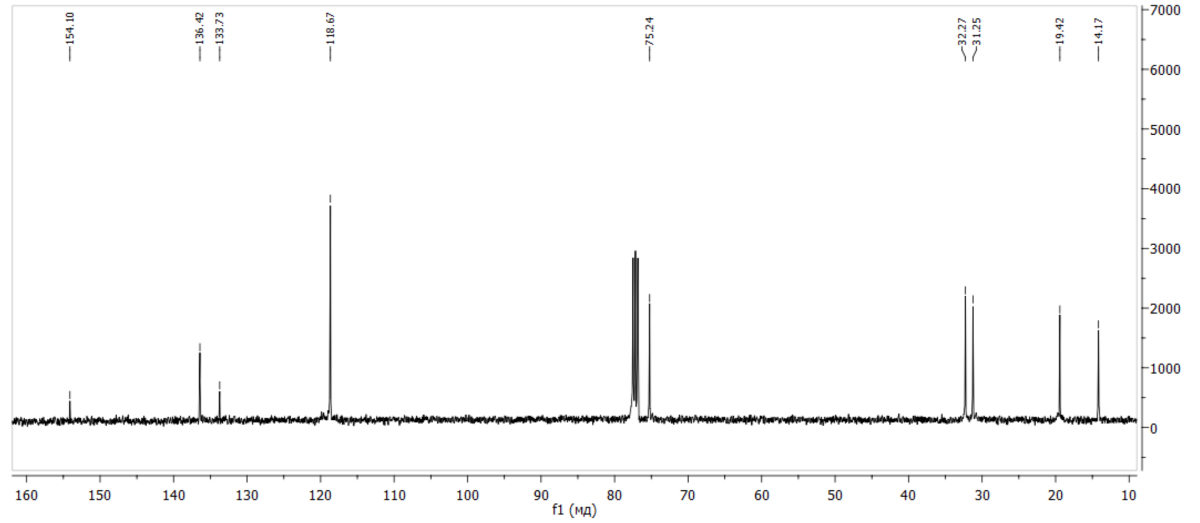

154.10
136.42
133.73
118.67
75.24
32.27
31.25
19.42
14.17
f1 (мд)

(b)

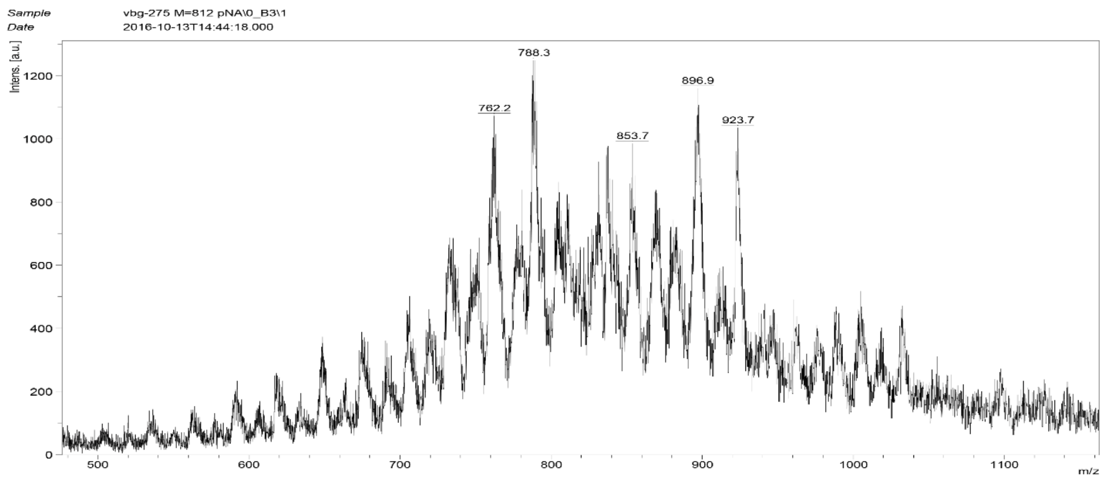

(c)

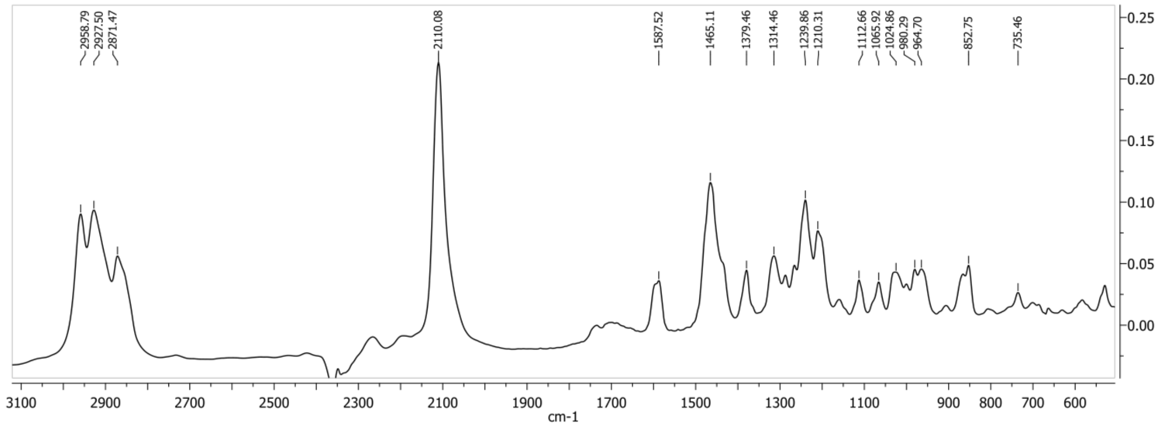

(d)

Figure S7: ^{1}H NMR (a), ^{13}C (b), MALDI TOF (c) and FTIR (d) spectra of **8a** (5,11,17,23-tetraazide-25,26,27,28-tetrabutoxy-calix[4]arene).

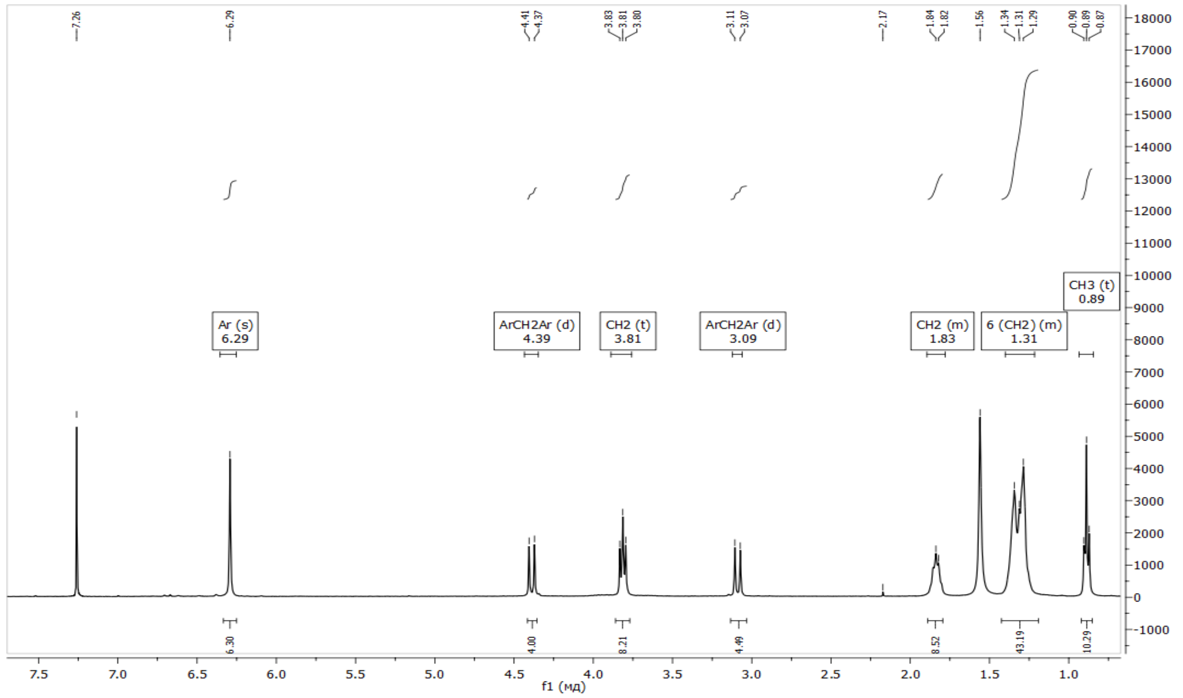

Ar (s)
6.29
ArCH2Ar (d)
4.39
CH2 (t)
3.81
ArCH2Ar (d)
3.09
CH2 (m)
1.83
6 (CH2) (m)
1.31
CH3 (t)
0.89
f1 (мд)

(a)

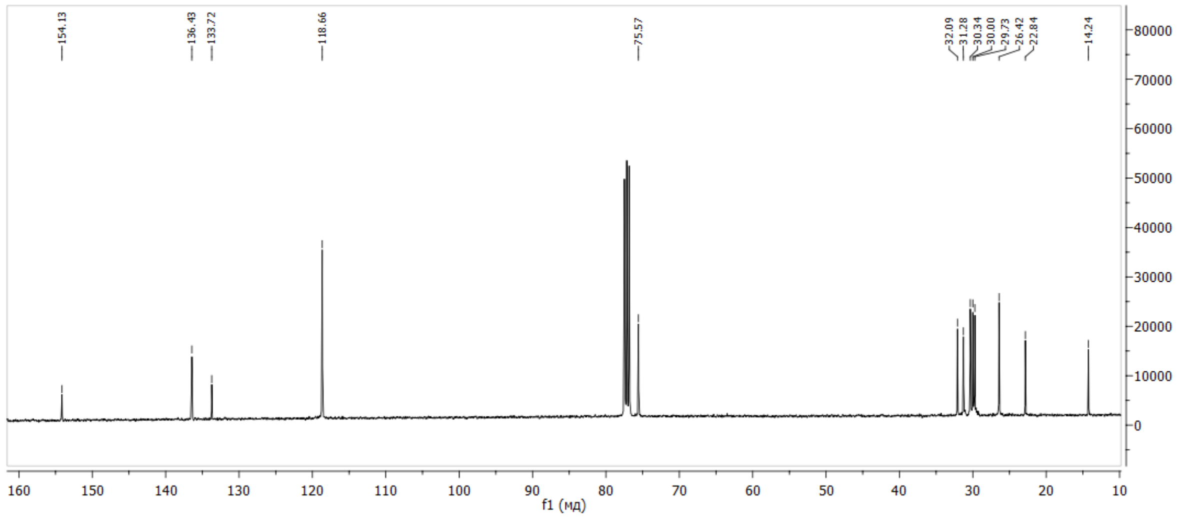

f1 (мд)

(b)

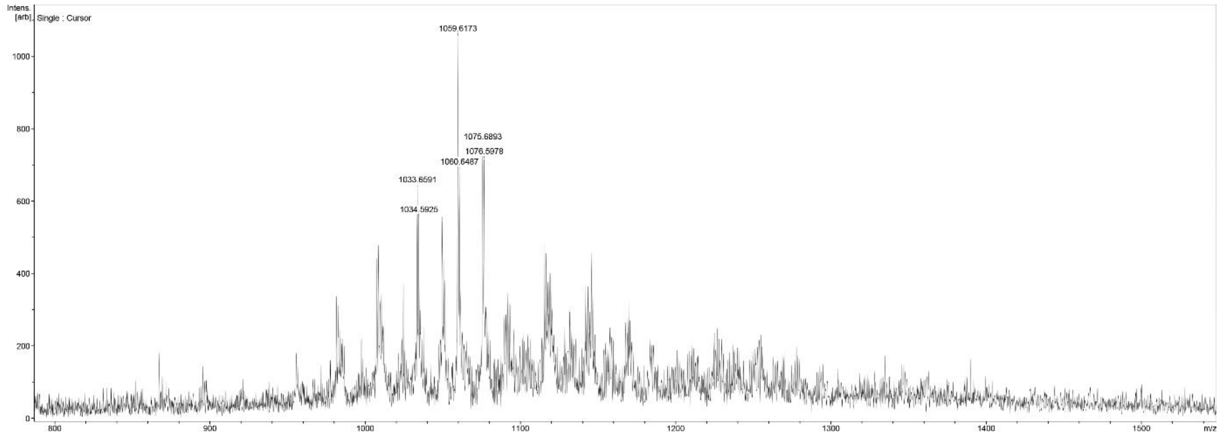

Bruker Daltonics flexControl

Display Screenshot - Generated On 2016-10-13 14h41m50s

(c)

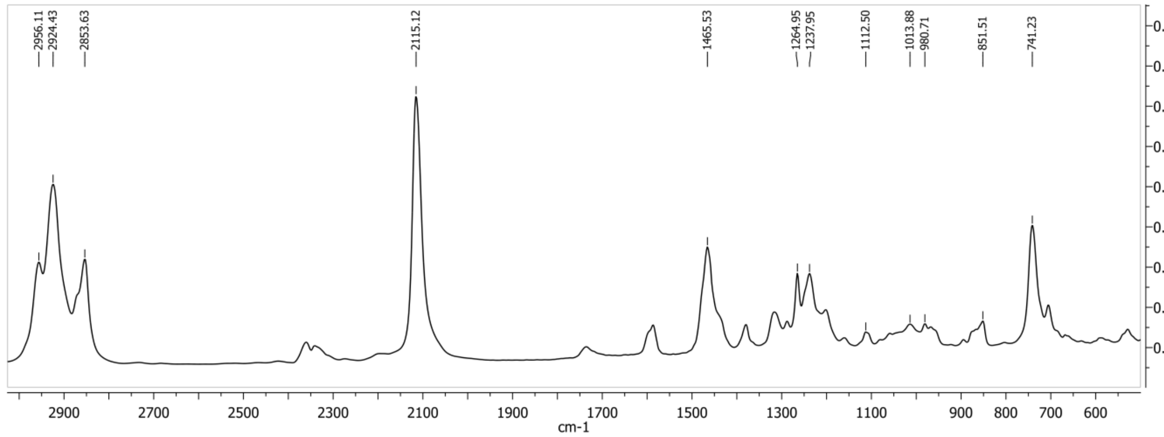

(d)

Figure S8: ^{1}H NMR (a), ^{13}C (b), MALDI TOF (c) and FTIR (d) spectra of **8b** (5,11,17,23-tetraazide-25,26,27,28-tetrabutoxy-calix[4]arene).

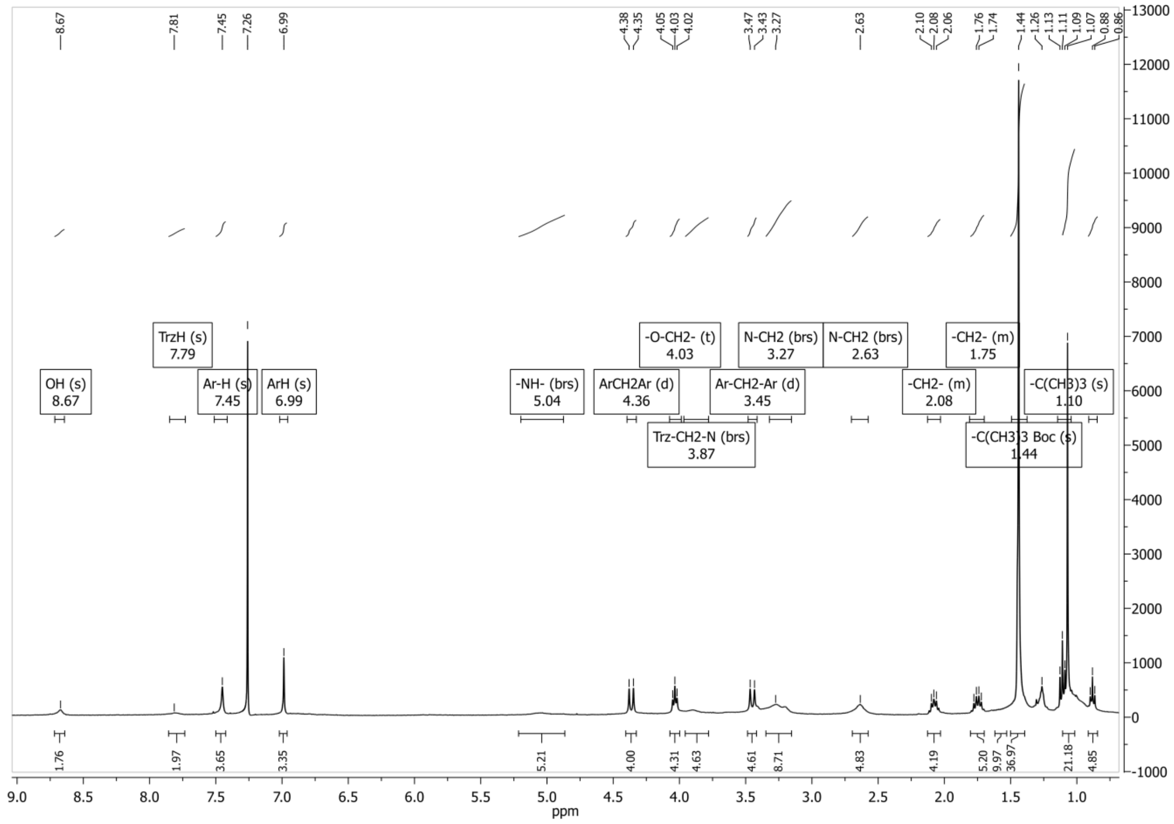

OH (s)
8.67
TrzH (s)
7.79
Ar-H (s)
7.45
ArH (s)
6.99
-NH- (brs)
5.04
ArCH2Ar (d)
4.36
-O-CH2- (t)
4.03
Trz-CH2-N (brs)
3.87
N-CH2 (brs)
3.27
Ar-CH2-Ar (d)
3.45
N-CH2 (brs)
2.63
-CH2- (m)
2.08
-CH2- (m)
1.75
-C(CH3)3 (s)
1.10
-C(CH3)3 Boc (s)
1.44
ppm

(a)

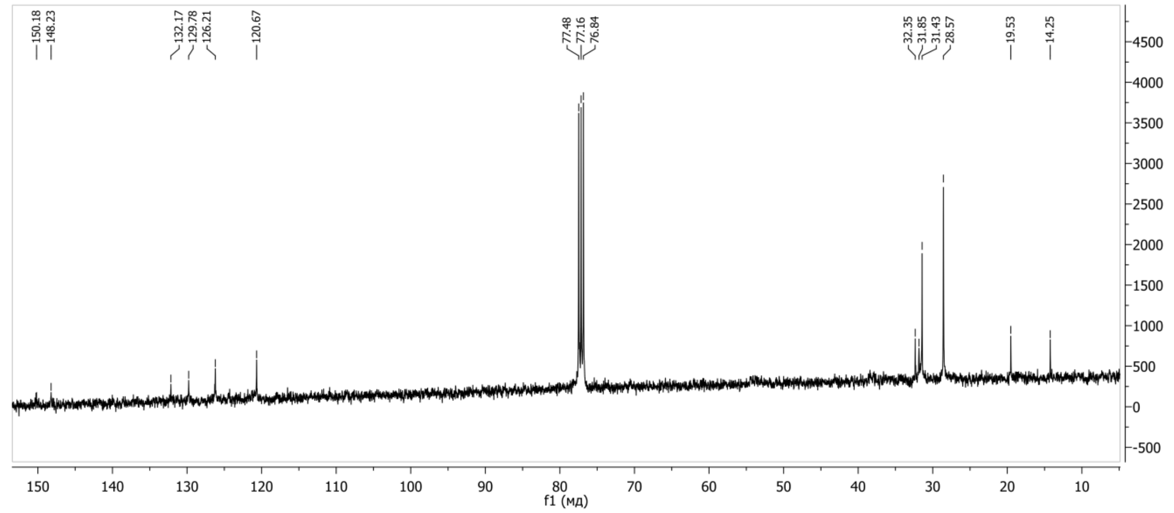

f1 (мд)

(b)

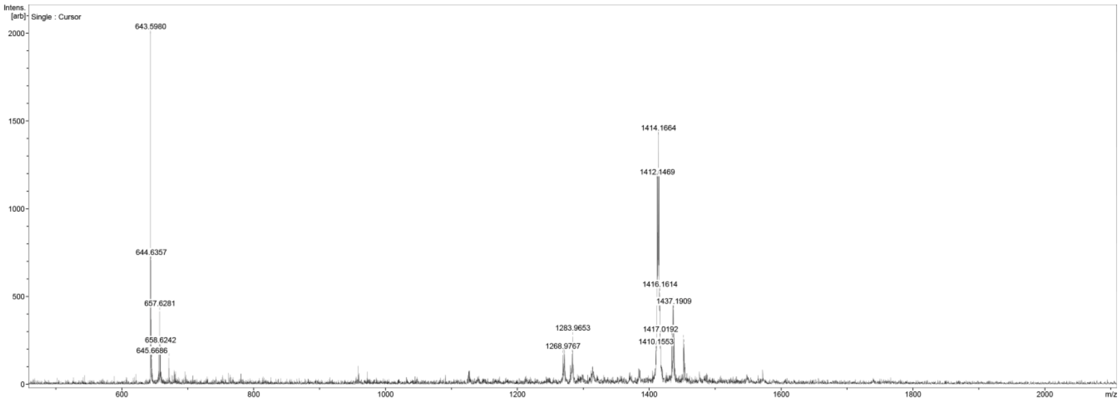

Bruker Daltonics flexControl

Display Screenshot - Generated On 2016-11-28 13h49m20s

(c)

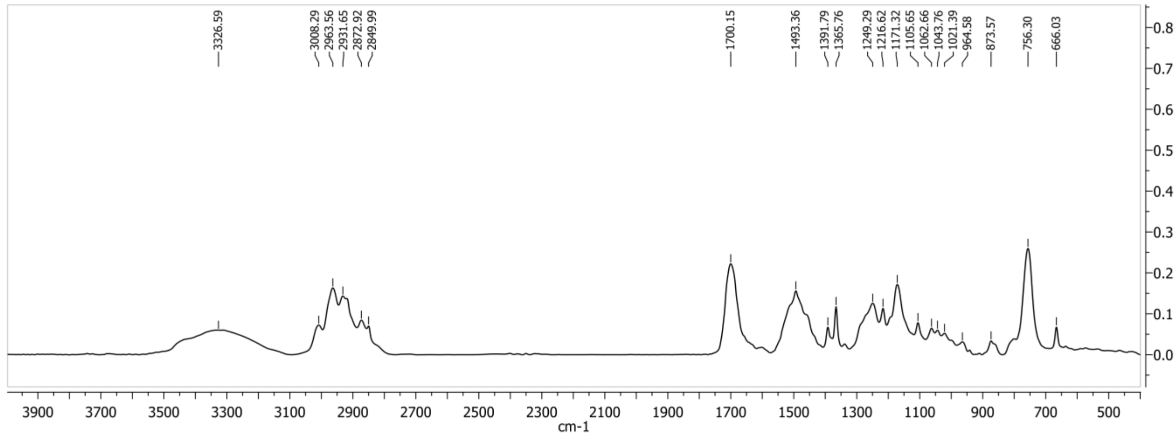

(d)

Figure S9: ^{1}H NMR (a), ^{13}C (b), MALDI TOF (c) and FTIR (d) spectra of **9a** (5,17-di-*tert*-butyl-11,23-bis(4-((bis(2-((*tert*-butoxycarbonyl)amino)ethyl)amino)methyl)-*1H*-1,2,3-triazol-1-yl))-25,27-dibutoxy-26,28-dihydroxy-calix[4]arene).

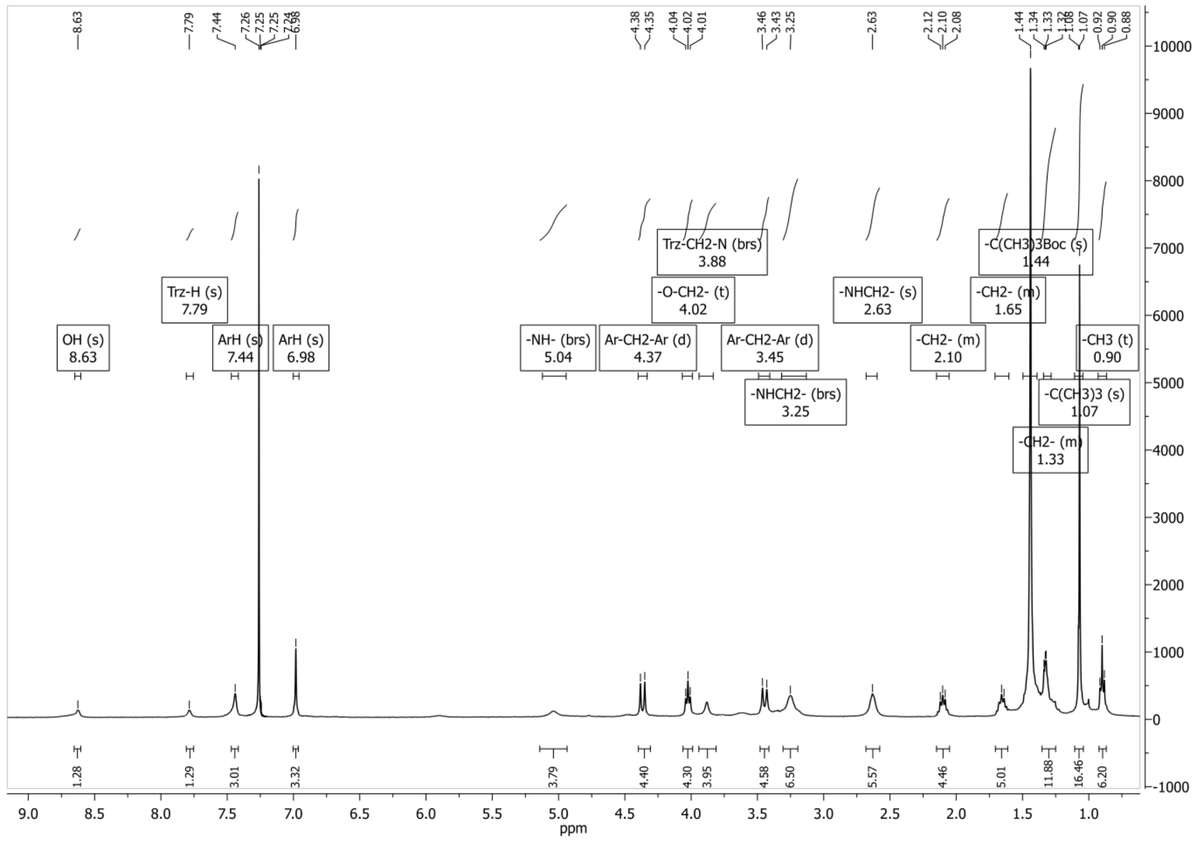
OH (s)
8.63
Trz-H (s)
7.79
ArH (s)
7.44
ArH (s)
6.98
-NH- (brs)
5.04
Ar-CH2-Ar (d)
4.37
Trz-CH2-N (brs)
3.88
-O-CH2- (t)
4.02
Ar-CH2-Ar (d)
3.45
-NHCH2- (brs)
3.25
-NHCH2- (s)
2.63
-CH2- (m)
2.10
-CH2- (m)
1.65
-C(CH3)3Boc (s)
1.44
-CH2- (m)
1.33
-C(CH3)3 (s)
1.07
-CH3 (t)
0.90
ppm

(a)

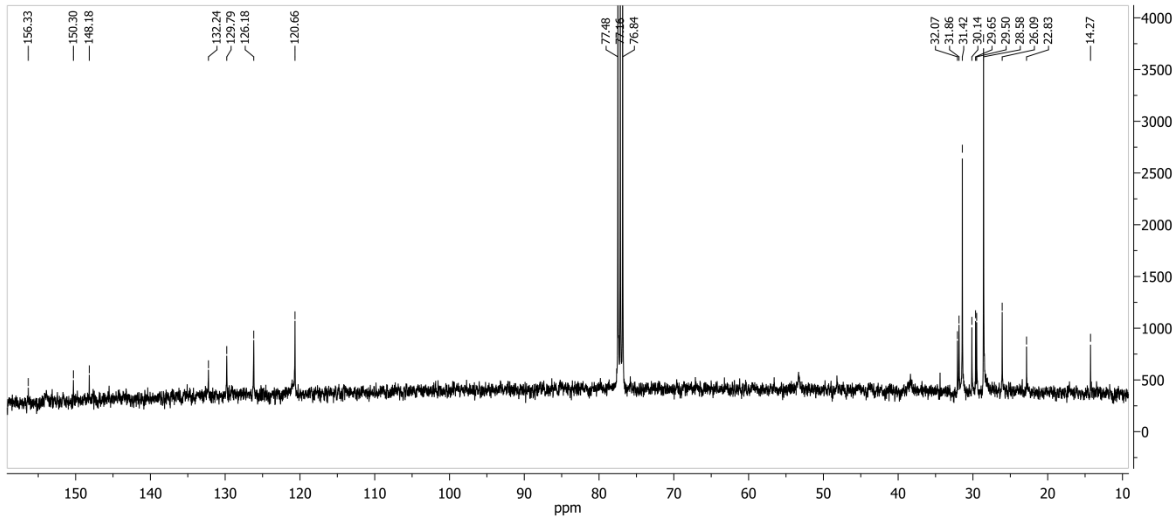
156.33
150.30
148.18
132.24
129.79
126.18
120.66
77.48
77.16
76.84
32.07
31.86
31.42
30.14
29.65
29.50
28.58
26.09
22.83
14.27
ppm

(b)

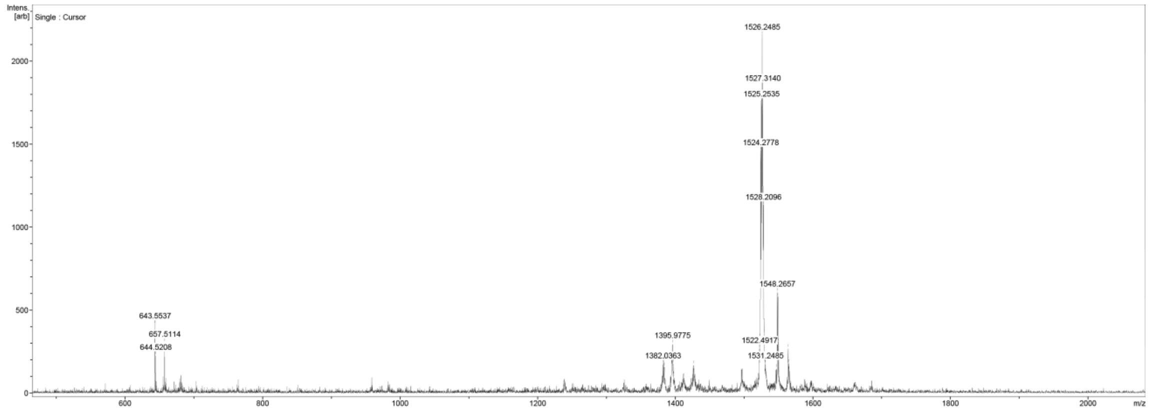

Bruker Daltonics flexControl

Display Screenshot - Generated On 2016-11-28 13h51m42s

(c)

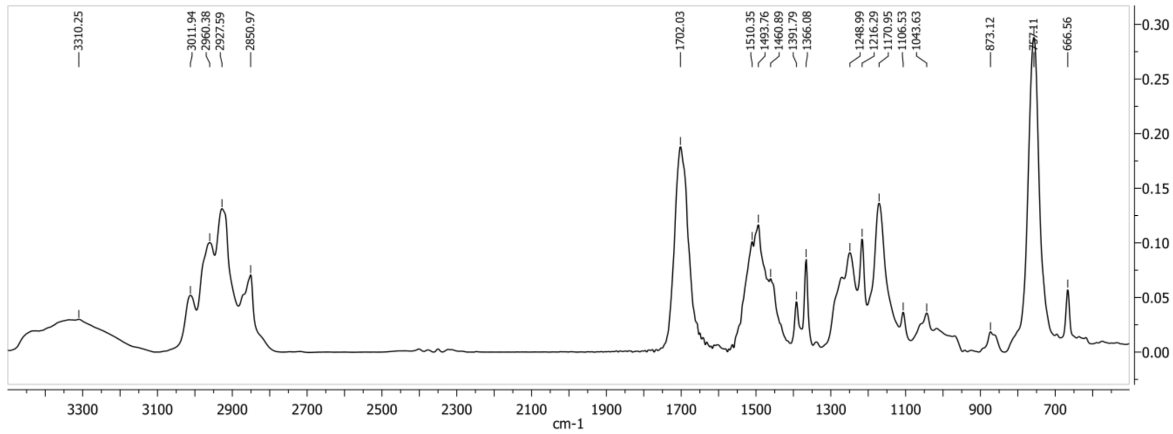

(d)

Figure S10: ^{1}H NMR (a), ^{13}C (b), MALDI TOF (c) and FTIR (d) spectra of **9b** (5,17-di-*tert*-butyl-11,23-bis(4-((bis(2-((*tert*-butoxycarbonyl)amino)ethyl)amino)methyl)-*1H*-1,2,3-triazol-1-yl))-25,27-dioctyloxy-26,28-dihydroxy-calix[4]arene).

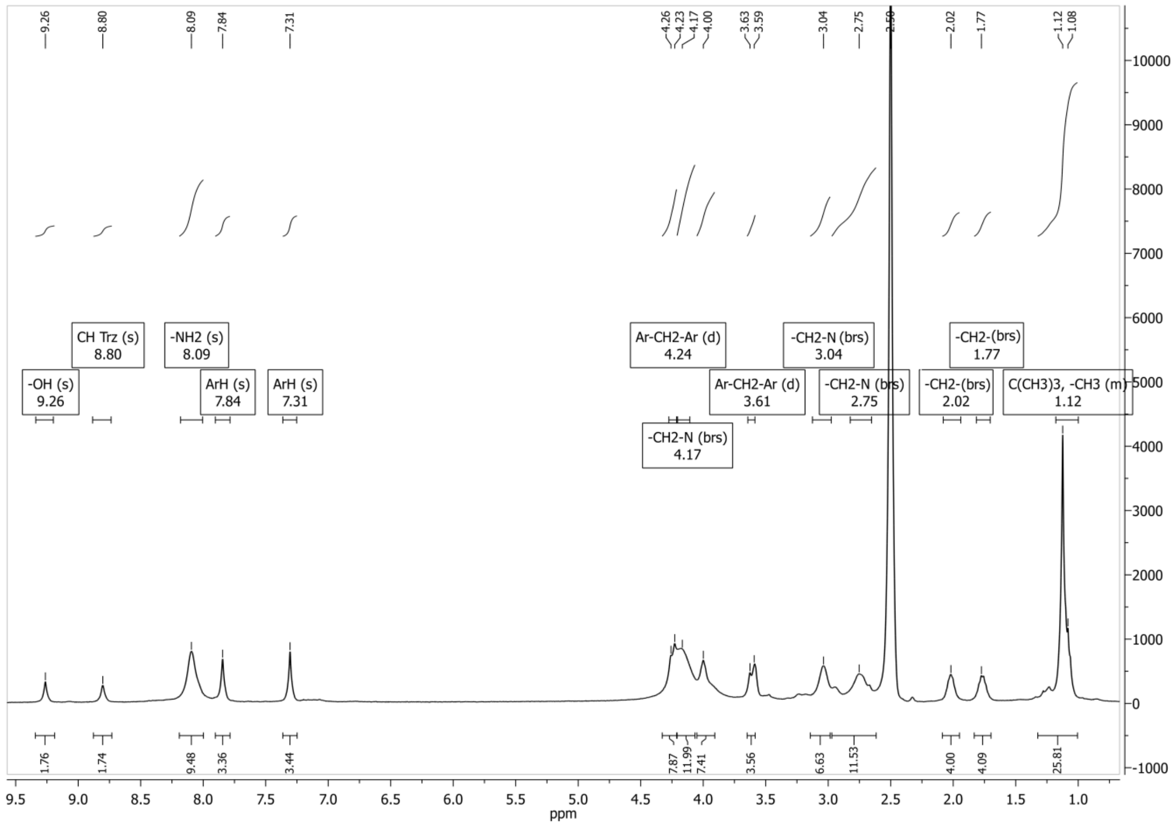
9.26
8.80
8.09
7.84
7.31
4.26
4.23
4.17
4.00
3.63
3.59
3.04
2.75
2.50
2.02
1.77
1.12
1.08
-OH (s)
9.26
CH Trz (s)
8.80
-NH2 (s)
8.09
ArH (s)
7.84
ArH (s)
7.31
Ar-CH2-Ar (d)
4.24
-CH2-N (brs)
4.17
Ar-CH2-Ar (d)
3.61
-CH2-N (brs)
3.04
-CH2-N (brs)
2.75
-CH2-(brs)
2.02
-CH2-(brs)
1.77
C(CH3)3, -CH3 (m)
1.12
1.76
1.74
9.48
3.36
3.44
7.87
11.99
7.41
3.56
6.63
11.53
4.00
4.09
25.81
ppm

(a)

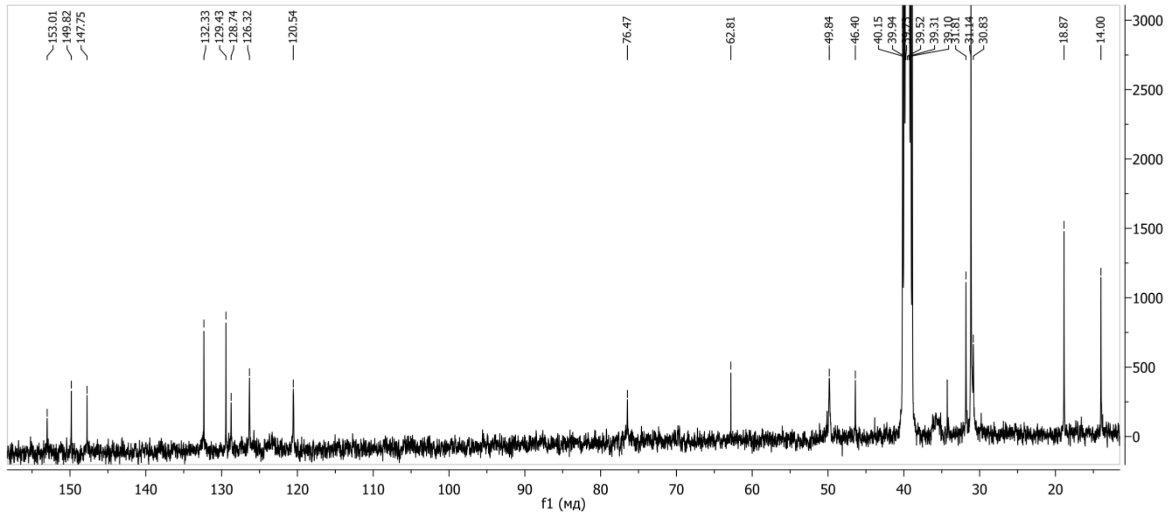
153.01
149.82
147.75
132.33
129.43
128.74
126.32
120.54
76.47
62.81
49.84
46.40
40.15
39.94
39.52
39.31
39.10
31.81
31.14
30.83
18.87
14.00
f1 (мд)

(b)

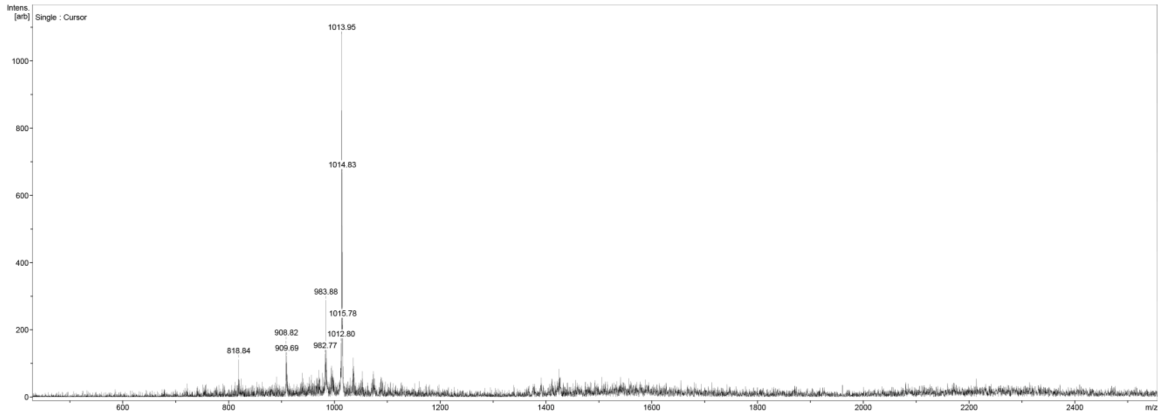

Bruker Daltonics flexControl

Display Screenshot - Generated On 2017-02-02 16h11m47s

(c)

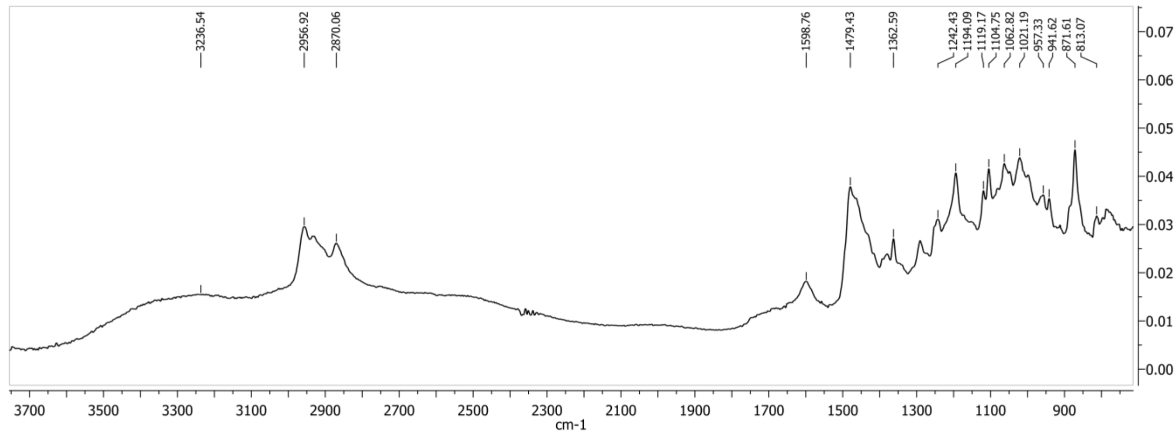

(d)

Figure S11: ^{1}H NMR (a), ^{13}C (b), MALDI TOF (c) and FTIR (d) spectra of **10a** (5,17-di-*tert*-butyl-11,23-bis(4-((bis(2-(amino)ethyl) amino)methyl)-*1H*-1,2,3-triazol-1-yl))-25,27-dibutoxy-26,28-dihydroxy-calix[4]arene dihydrochloride).

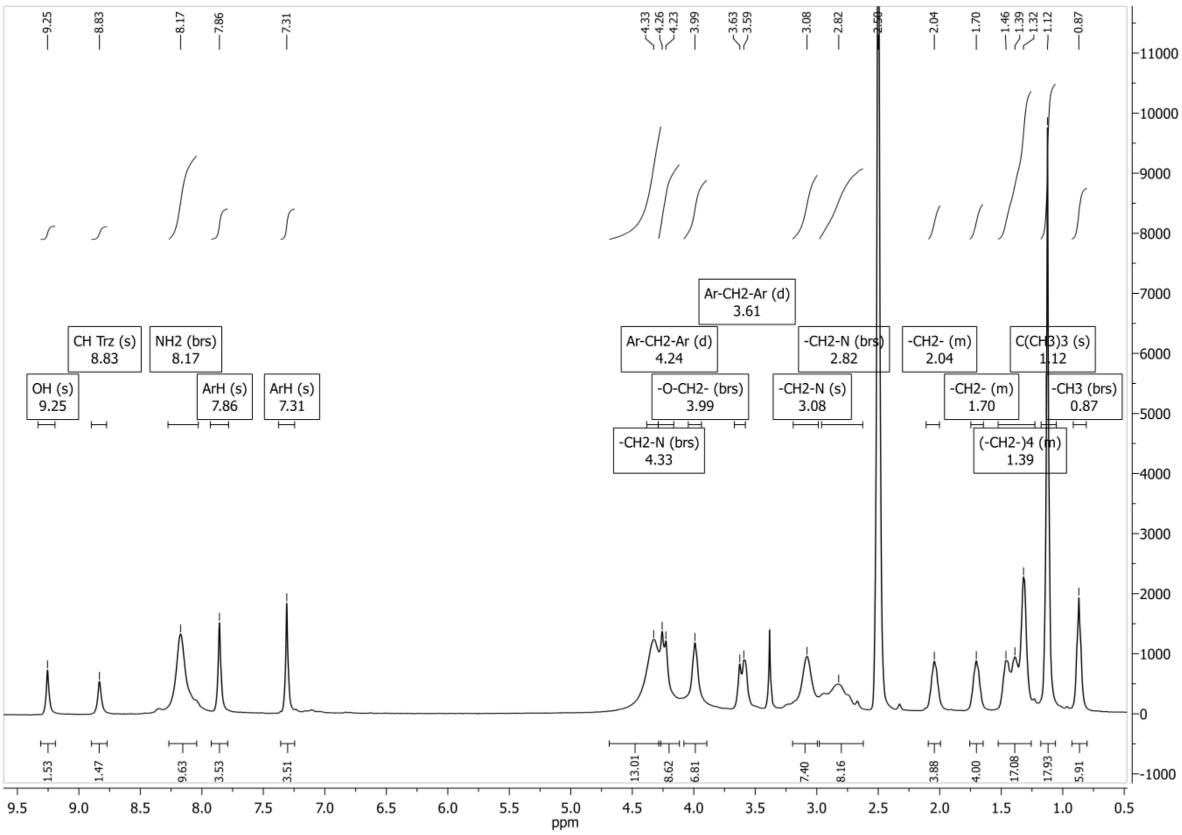
OH (s) 9.25
CH Trz (s) 8.83
NH2 (brs) 8.17
ArH (s) 7.86
ArH (s) 7.31
Ar-CH2-Ar (d) 4.24
Ar-CH2-Ar (d) 3.61
-O-CH2- (brs) 3.99
-CH2-N (brs) 4.33
-CH2-N (s) 3.08
-CH2-N (brs) 2.82
-CH2- (m) 2.04
-CH2- (m) 1.70
(-CH2-)4 (m) 1.39
C(CH3)3 (s) 1.12
-CH3 (brs) 0.87
ppm

(a)

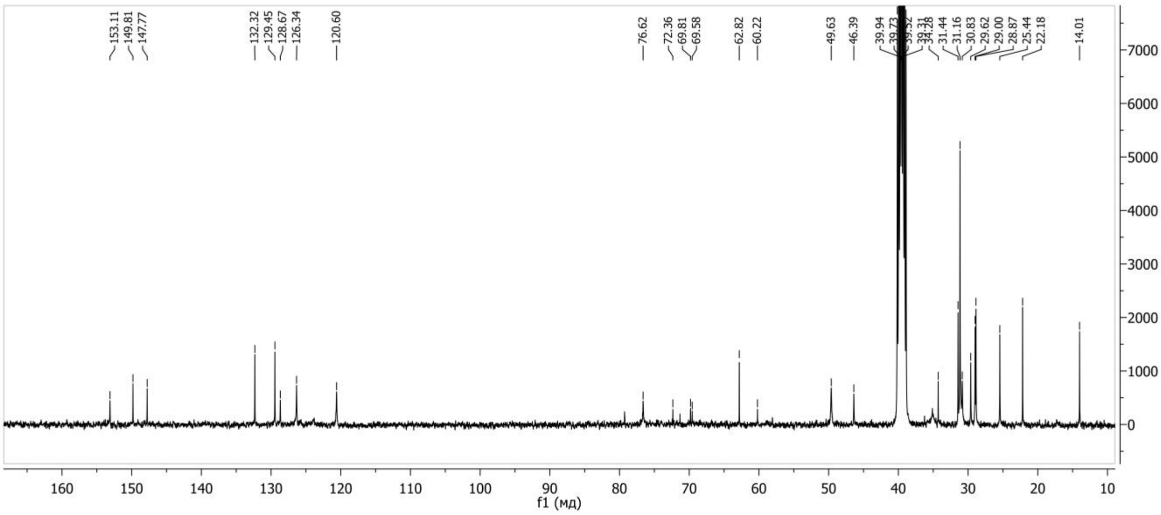
f1 (мд)

(b)

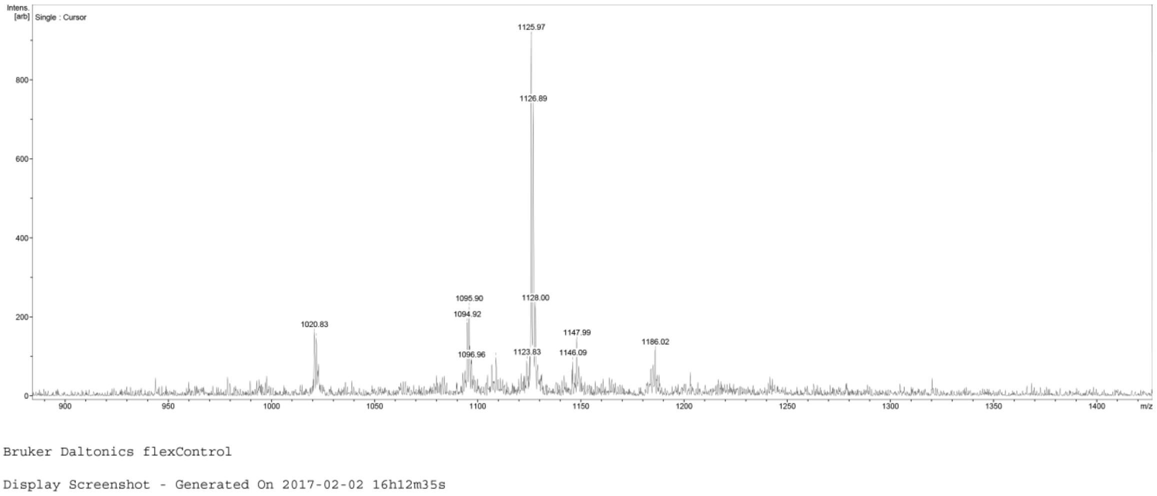

(c)

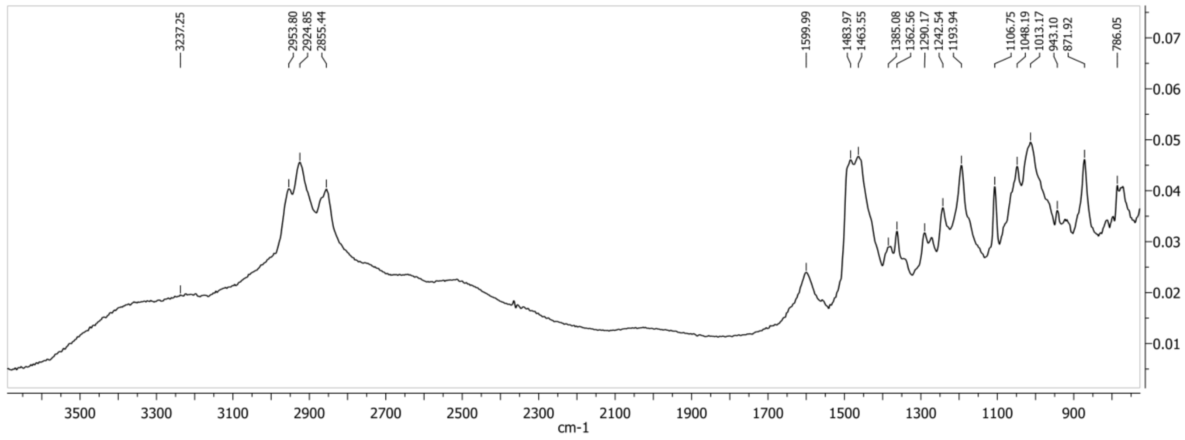

(d)

Figure S12: ^{1}H NMR (a), ^{13}C (b), MALDI TOF (c) and FTIR (d) spectra of **10b** (5,17-di-*tert*-butyl-11,23-bis(4-((bis(2-(amino)ethyl)amino)methyl)-*1H*-1,2,3-triazol-1-yl))-25,27-dioctyloxy-26,28-dihydroxy-calix[4]arene dihydrochloride).

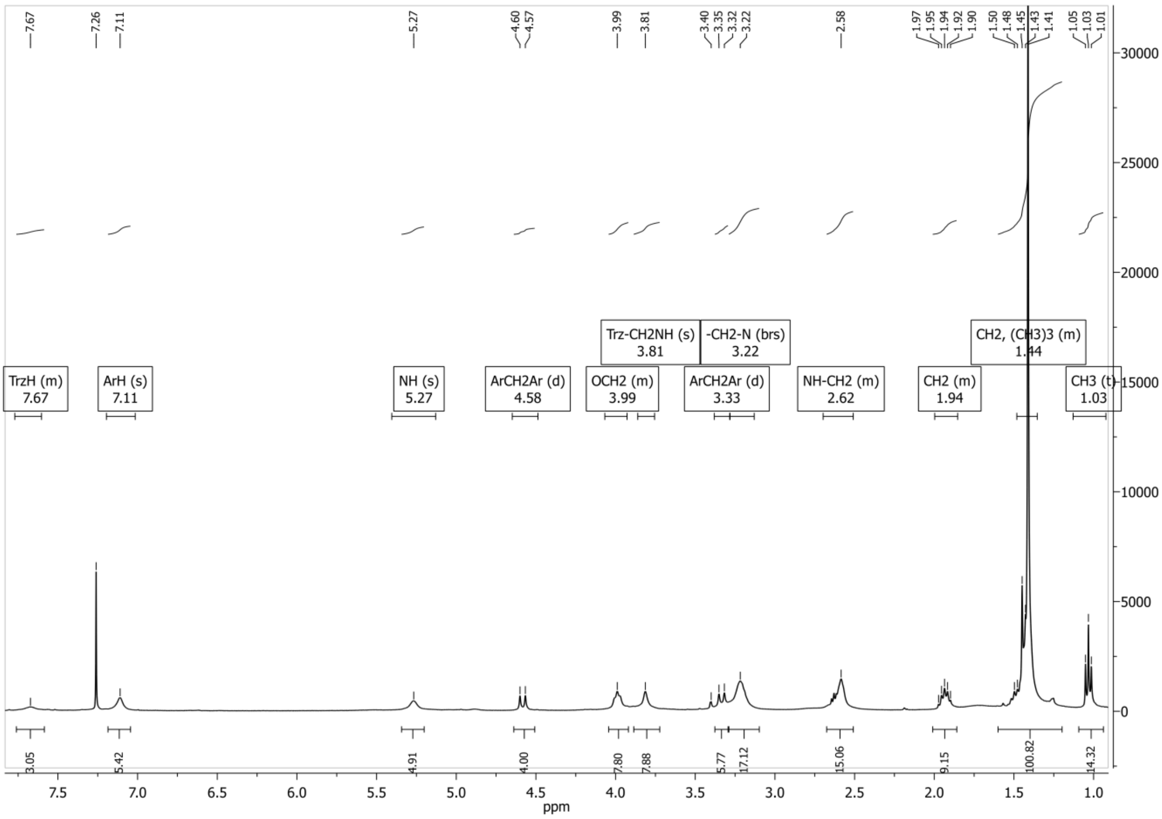
7.67
7.26
7.11
5.27
4.60
4.57
3.99
3.81
3.40
3.35
3.32
3.22
2.58
1.97
1.95
1.94
1.92
1.90
1.50
1.48
1.45
1.43
1.41
1.05
1.03
1.01
Trz-CH2NH (s)
3.81
-CH2-N (brs)
3.22
CH2, (CH3)3 (m)
1.44
TrzH (m)
7.67
ArH (s)
7.11
NH (s)
5.27
ArCH2Ar (d)
4.58
OCH2 (m)
3.99
ArCH2Ar (d)
3.33
NH-CH2 (m)
2.62
CH2 (m)
1.94
CH3 (t)
1.03
3.05
5.42
4.91
4.00
7.80
7.88
5.77
17.12
15.06
9.15
100.82
14.32
ppm

(a)

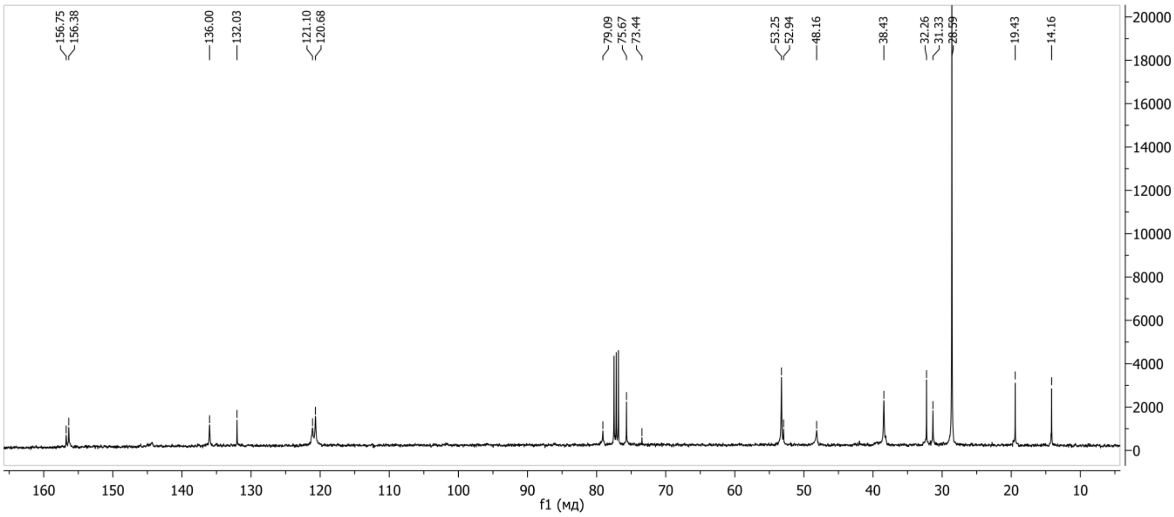
156.75
156.38
136.00
132.03
121.10
120.68
79.09
75.67
73.44
53.25
52.94
48.16
38.43
32.26
31.33
28.59
19.43
14.16
f1 (мд)

(b)

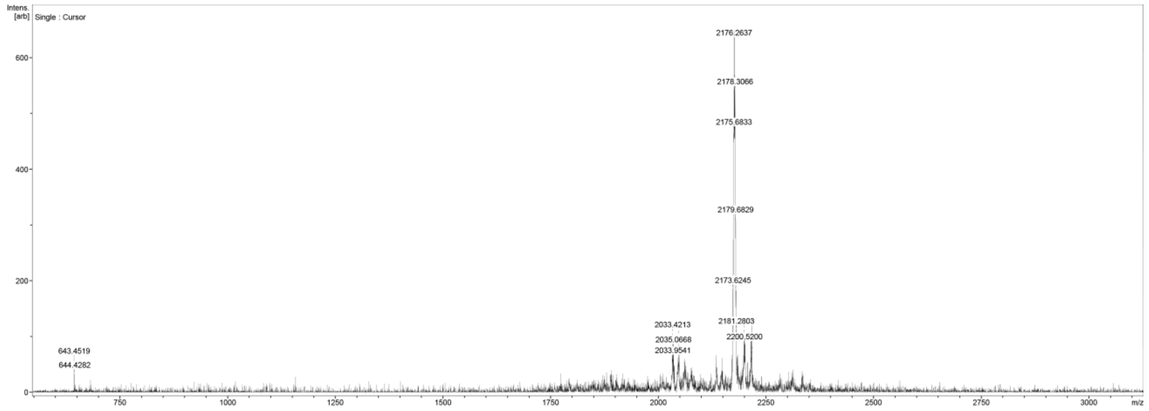

Bruker Daltonics flexControl

Display Screenshot - Generated On 2016-11-17 11h13m24s

(c)

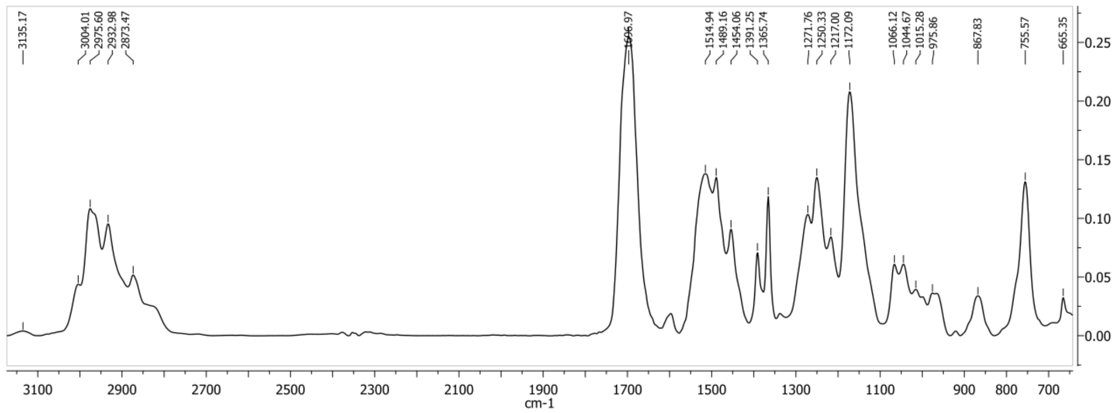

(d)

Figure S13: ^{1}H NMR (a), ^{13}C (b), MALDI TOF (c) and FTIR (d) spectra of **11a** (5,11,17,23-tetra(4-((bis(2-((*tert*-butoxycarbonyl)amino)ethyl)amino)methyl)-1*H*-1,2,3-triazol-1-yl))-25,26,27,28-tetrabutoxycalix[4]arene.

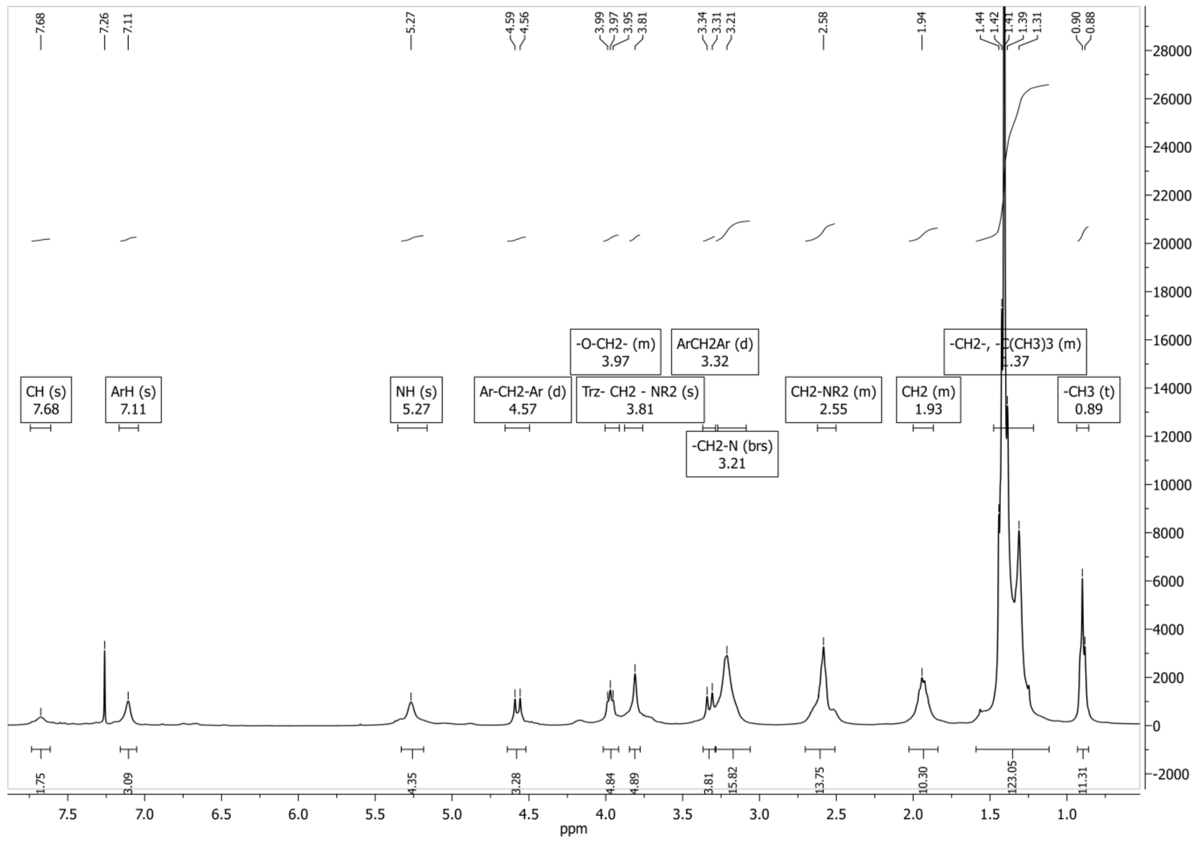
7.68
7.26
7.11
5.27
4.59
4.56
3.99
3.97
3.95
3.81
3.34
3.31
3.21
2.58
1.94
1.44
1.42
1.41
1.39
1.31
0.90
0.88
CH (s)
7.68
ArH (s)
7.11
NH (s)
5.27
Ar-CH2-Ar (d)
4.57
-O-CH2- (m)
3.97
Trz- CH2 - NR2 (s)
3.81
ArCH2Ar (d)
3.32
-CH2-N (brs)
3.21
CH2-NR2 (m)
2.55
CH2 (m)
1.93
-CH2-, -C(CH3)3 (m)
1.37
-CH3 (t)
0.89
1.75
3.09
4.35
3.28
4.84
4.89
3.81
15.82
13.75
10.30
123.05
11.31
ppm

(a)

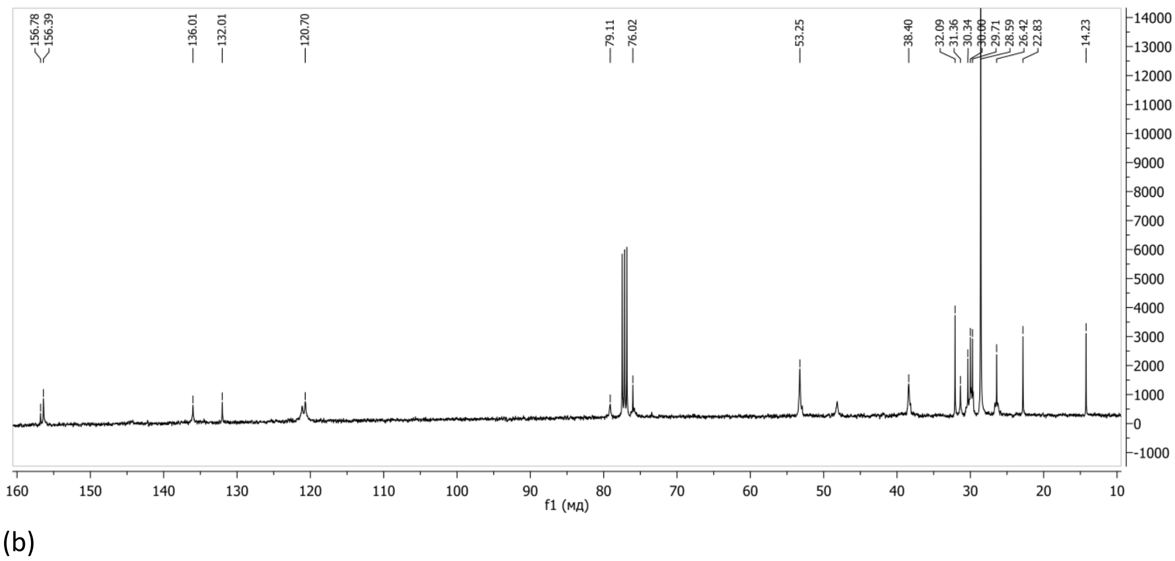
156.78
156.39
136.01
132.01
120.70
79.11
76.02
53.25
38.40
32.09
31.36
30.34
30.00
29.71
28.59
26.42
22.83
14.23
f1 (мд)

(b)

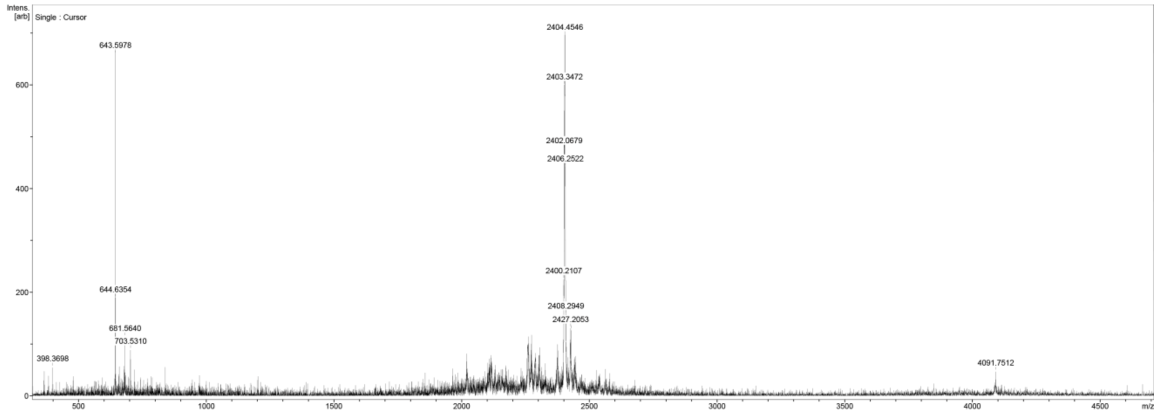

Bruker Daltonics flexControl

Display Screenshot - Generated On 2016-12-08 11h15m02s

(c)

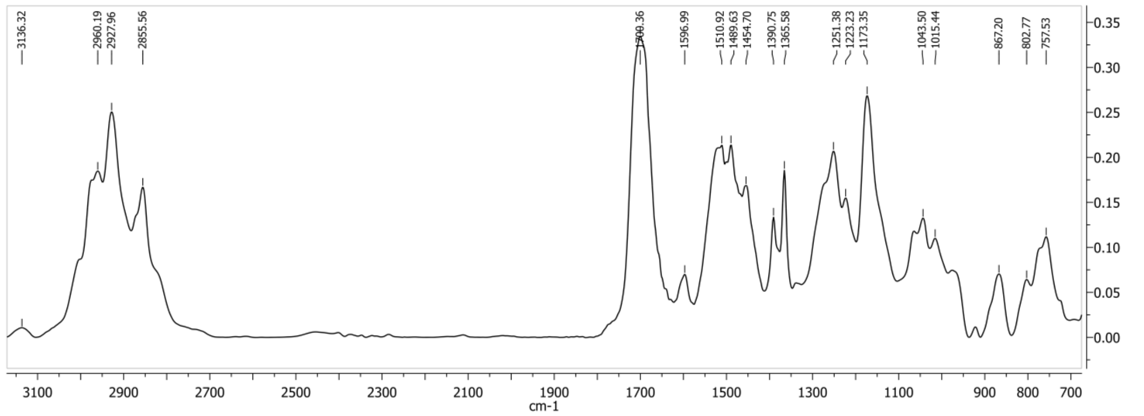

(d)

Figure S14: ^{1}H NMR (a), ^{13}C (b), MALDI TOF (c) and FTIR (d) spectra of **11b** (5,11,17,23-tetra(4-((bis(2-((*tert*-butoxycarbonyl)amino)ethyl)amino)methyl)-1*H*-1,2,3-triazol-1-yl))-25,26,27,28-tetraoctyloxycalix[4]arene.

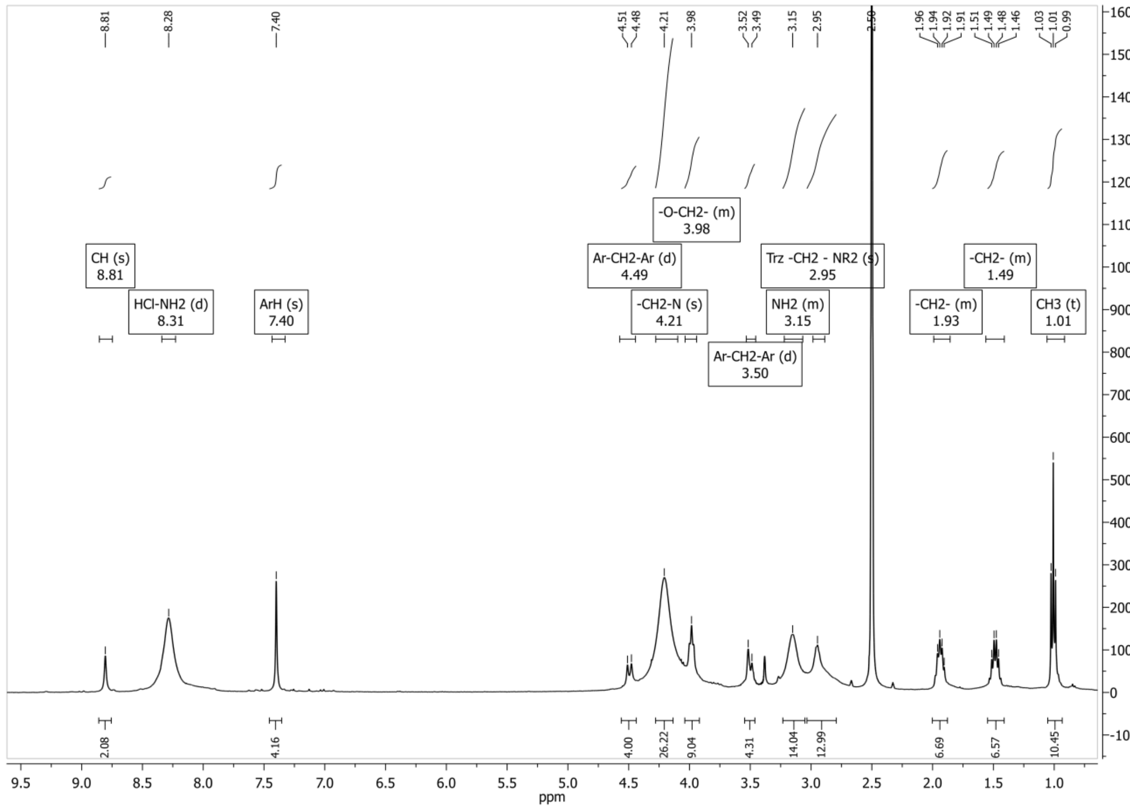

CH (s)
8.81
HCl-NH2 (d)
8.31
ArH (s)
7.40
Ar-CH2-Ar (d)
4.49
-O-CH2- (m)
3.98
-CH2-N (s)
4.21
Ar-CH2-Ar (d)
3.50
Trz -CH2 - NR2 (s)
2.95
NH2 (m)
3.15
-CH2- (m)
1.93
-CH2- (m)
1.49
CH3 (t)
1.01
ppm

(a)

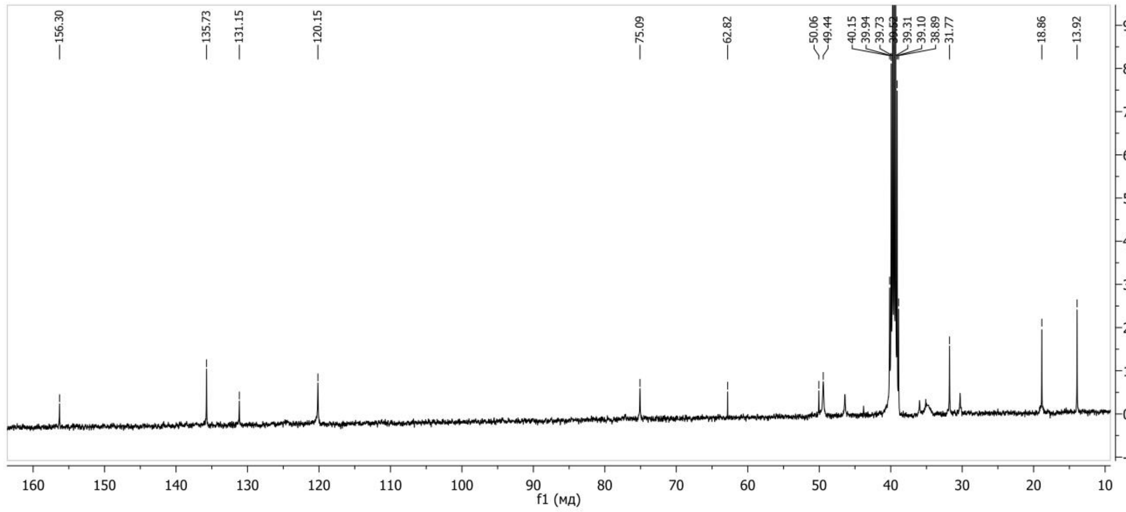

f1 (мд)

(b)

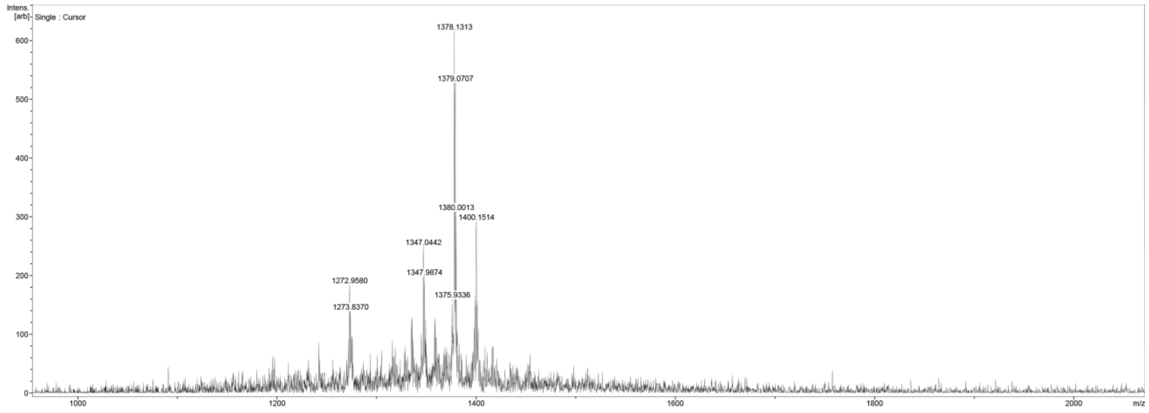

Bruker Daltonics flexControl

Display Screenshot - Generated On 2016-11-29 17h37m52s

(c)

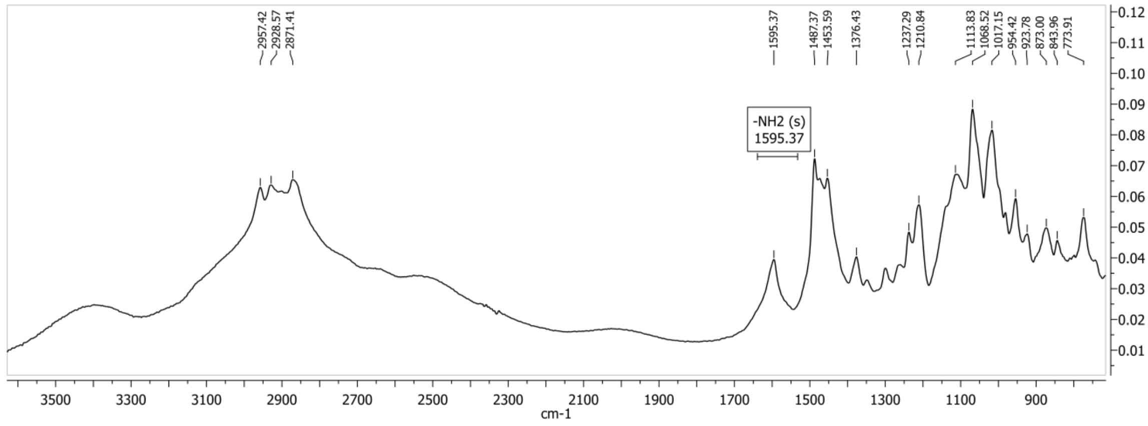

(d)

Figure S15: ^{1}H NMR (a), ^{13}C (b), MALDI TOF (c) and FTIR (d) spectra of **12a** (5,11,17,23-tetra(4-((bis(2-(amino)ethyl)amino)methyl)-1*H*-1,2,3-triazol-1-yl))-25,26,27,28-tetrabutoxycalix[4]arene tetrahydrochloride):

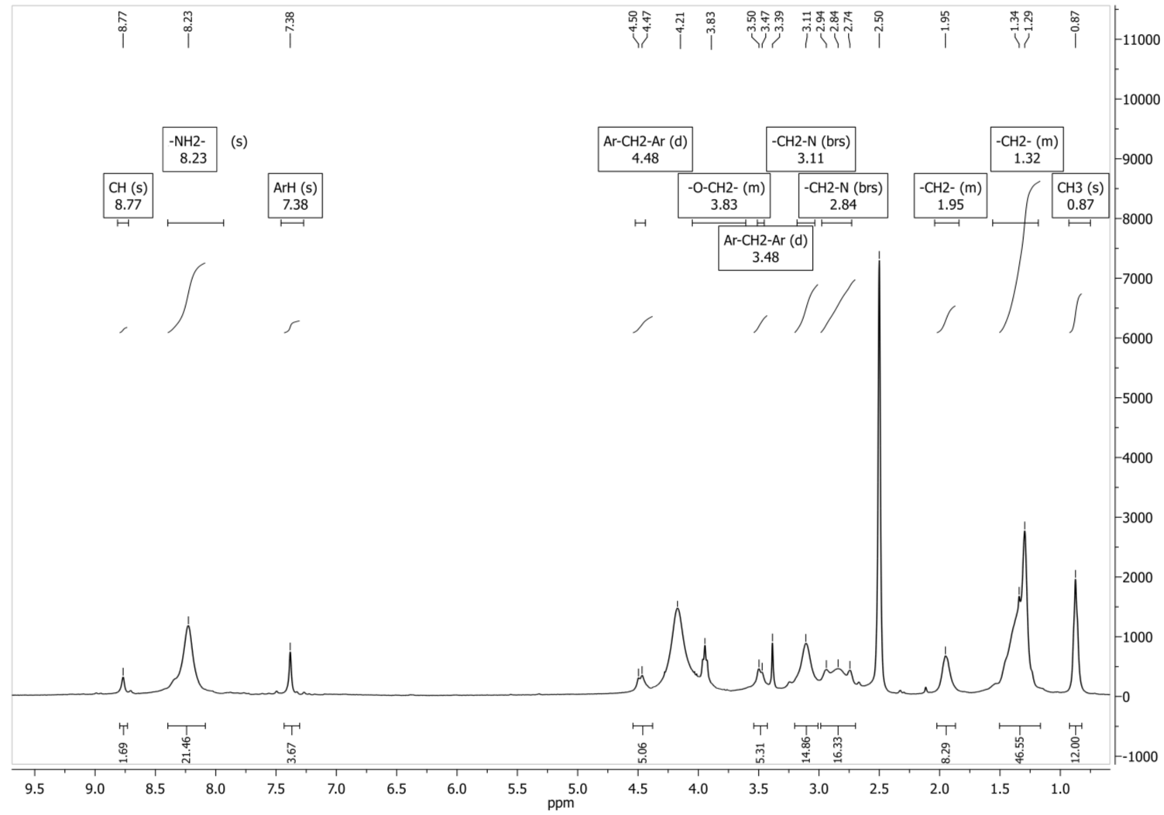

8.77
8.23
7.38
4.50
4.47
4.21
3.83
3.50
3.47
3.39
3.11
2.94
2.84
2.74
2.50
1.95
1.34
1.29
0.87
-NH2- (s)
8.23
Ar-CH2-Ar (d)
4.48
-CH2-N (brs)
3.11
-CH2- (m)
1.32
CH (s)
8.77
ArH (s)
7.38
-O-CH2- (m)
3.83
-CH2-N (brs)
2.84
-CH2- (m)
1.95
CH3 (s)
0.87
Ar-CH2-Ar (d)
3.48
1.69
21.46
3.67
5.06
5.31
14.86
16.33
8.29
46.55
12.00
ppm

(a)

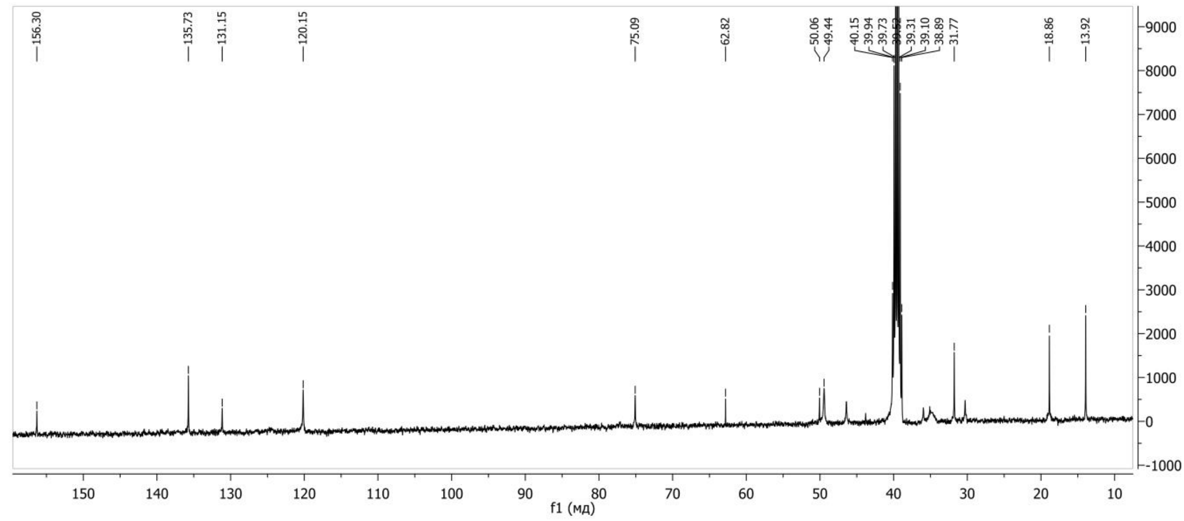

156.30
135.73
131.15
120.15
75.09
62.82
50.06
49.44
40.15
39.94
39.73
39.31
39.10
38.89
31.77
18.86
13.92
f1 (мд)

(b)

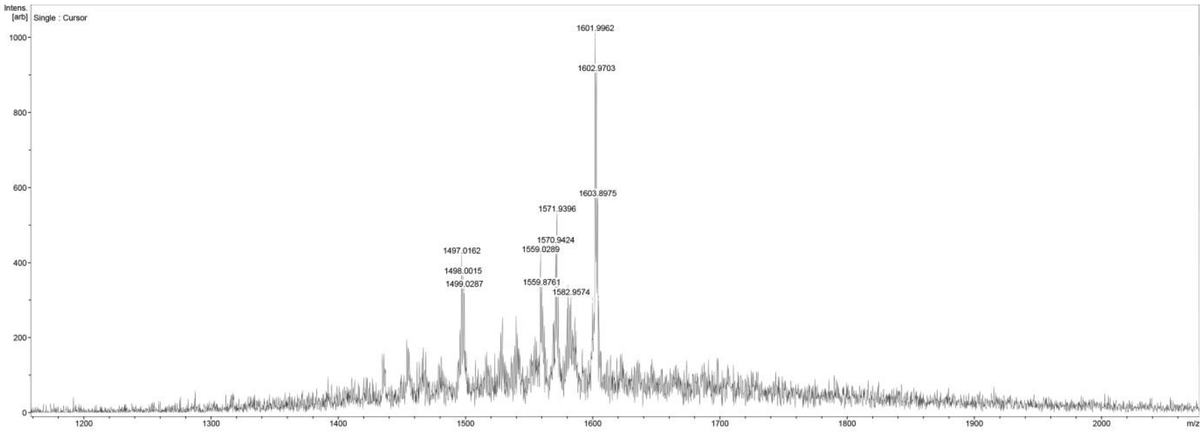

Bruker Daltonics flexControl

Display Screenshot - Generated On 2016-11-17 10h59m32s

(c)

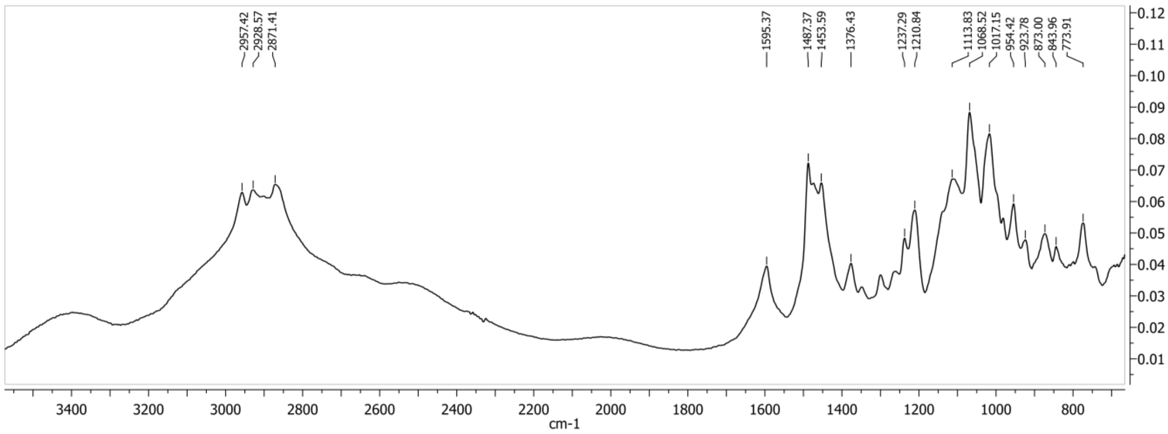

(d)

Figure S16: ^{1}H NMR (a), ^{13}C (b), MALDI TOF (c) and FTIR (d) spectra of **12b** (5,11,17,23-tetra(4-((bis(2-(amino)ethyl)amino)methyl)-1*H*-1,2,3-triazol-1-yl))-25,26,27,28-tetrabutoxycalix[4]arene tetrahydrochloride).

Table S1: Crystal data and structure refinement for **8a** .

Identification code	***8a***	
Empirical formula	C50 H60 O4	
Formula weight	724.98	
Temperature	100(2) K	
Wavelength	0.96990 Å	
Crystal system	Monoclinic	
Space group	$P2_1/n$	
Unit cell dimensions	a = 12.347(3) Å	α = 90°.
	b = 31.269(6) Å	β = 112.48(3)°.
	c = 12.452(3) Å	γ = 90°.
Volume	4442(2) $Å^3$	
Z	4	
Density (calculated)	1.084 Mg/m^3	
Absorption coefficient	0.136 mm^{-1}	
F(000)	1568	
Crystal size	0.30 x 0.30 x 0.05 mm^3	
Theta range for data collection	2.574 to 38.404°.	
Index ranges	-13<=h<=13, -39<=k<=39, -15<=l<=15	
Reflections collected	52446	
Independent reflections	8997 [R(int) = 0.0727]	
Completeness to theta = 35.587°	96.9 %	
Absorption correction	Semi-empirical from equivalents	
Max. and min. transmission	0.990 and 0.950	
Refinement method	Full-matrix least-squares on F^2	
Data / restraints / parameters	8997 / 0 / 506	
Goodness-of-fit on F^2	0.993	
Final R indices [for 6273 rflns with $I>2\sigma(I)$]	R1 = 0.0800, wR2 = 0.1784	
R indices (all data)	R1 = 0.1009, wR2 = 0.1900	
Extinction coefficient	0.0092(9)	
Largest diff. peak and hole	0.361 and -0.349 e.$Å^{-3}$	

Table S2: Atomic coordinates (x 10^4) and equivalent isotropic displacement parameters (Å^2 x 10^3) for **8a**. U(eq) is defined as one third of the trace of the orthogonalized U^{ij} tensor.

Atom	x	y	z	U(eq)
O(1)	9202(1)	2836(1)	3830(1)	23(1)
O(2)	7921(1)	2908(1)	5294(1)	23(1)
O(3)	5562(1)	2514(1)	4253(1)	23(1)
O(4)	6483(1)	2681(1)	2534(1)	26(1)
C(1)	8323(1)	3406(1)	2434(1)	20(1)
C(2)	8067(1)	3843(1)	2254(1)	22(1)
C(3)	8479(2)	4146(1)	3147(1)	24(1)
C(4)	9183(1)	3996(1)	4256(1)	24(1)
C(5)	9446(1)	3564(1)	4487(1)	22(1)
C(6)	9022(1)	3273(1)	3559(1)	20(1)
C(7)	10069(1)	3418(1)	5742(1)	23(1)
C(8)	9272(1)	3470(1)	6406(1)	21(1)
C(9)	9486(1)	3787(1)	7255(1)	22(1)
C(10)	8758(2)	3862(1)	7865(1)	21(1)
C(11)	7765(2)	3598(1)	7578(1)	24(1)
C(12)	7489(1)	3282(1)	6723(1)	19(1)
C(13)	8248(1)	3220(1)	6132(1)	21(1)
C(14)	6381(1)	3018(1)	6391(1)	22(1)
C(15)	5446(1)	3171(1)	5242(1)	22(1)
C(16)	4991(2)	3586(1)	5164(1)	24(1)
C(17)	4198(1)	3758(1)	4128(1)	26(1)
C(18)	3867(1)	3496(1)	3142(1)	24(1)
C(19)	4294(1)	3082(1)	3170(1)	21(1)
C(20)	5070(1)	2922(1)	4235(1)	21(1)
C(21)	3967(2)	2830(1)	2035(1)	24(1)
C(22)	4609(2)	3003(1)	1300(1)	22(1)
C(23)	4029(1)	3261(1)	334(1)	22(1)
C(24)	4578(2)	3455(1)	-329(1)	22(1)
C(25)	5792(2)	3380(1)	30(1)	23(1)
C(26)	6424(1)	3128(1)	994(1)	21(1)
C(27)	5823(2)	2936(1)	1615(1)	22(1)
C(28)	7743(1)	3091(1)	1435(1)	21(1)
C(29)	8174(2)	4625(1)	2964(1)	29(1)
C(30)	7741(2)	4785(1)	3893(1)	41(1)
C(31)	7185(2)	4715(1)	1770(1)	42(1)

C(32)	9274(2)	4875(1)	3054(2)	46(1)
C(33)	10104(1)	2627(1)	3554(1)	25(1)
C(34)	11283(2)	2812(1)	4149(1)	25(1)
C(35)	12243(2)	2954(1)	4596(2)	33(1)
C(36)	8984(2)	4232(1)	8736(1)	26(1)
C(37)	10300(2)	4326(1)	9355(2)	36(1)
C(38)	8509(2)	4135(1)	9676(2)	38(1)
C(39)	8368(2)	4629(1)	8056(2)	42(1)
C(40)	3716(2)	4218(1)	4027(2)	32(1)
C(41)	4256(2)	4463(1)	5173(2)	47(1)
C(42)	4023(2)	4463(1)	3108(2)	44(1)
C(43)	2390(2)	4204(1)	3653(2)	58(1)
C(44)	4842(2)	2154(1)	4306(1)	26(1)
C(45)	5327(2)	1953(1)	5465(1)	26(1)
C(46)	5722(2)	1788(1)	6397(2)	33(1)
C(47)	3926(2)	3732(1)	-1400(1)	26(1)
C(48)	2780(2)	3911(1)	-1371(2)	40(1)
C(49)	4673(2)	4114(1)	-1486(2)	43(1)
C(50)	3617(2)	3455(1)	-2501(1)	42(1)

Table S3: Bond lengths [Å] and angles [°] for **8a**.

O(1)-C(6)	1.4069(18)	C(16)-H(16)	0.9500
O(1)-C(33)	1.4396(19)	C(17)-C(18)	1.401(2)
O(2)-C(13)	1.3710(18)	C(17)-C(40)	1.544(2)
O(2)-H(2O)	0.913(17)	C(18)-C(19)	1.394(2)
O(3)-C(20)	1.4113(18)	C(18)-H(18)	0.9500
O(3)-C(44)	1.4508(18)	C(19)-C(20)	1.398(2)
O(4)-C(27)	1.3770(19)	C(19)-C(21)	1.531(2)
O(4)-H(4O)	0.871(18)	C(21)-C(22)	1.521(2)
C(1)-C(6)	1.398(2)	C(21)-H(21A)	0.9900
C(1)-C(2)	1.399(2)	C(21)-H(21B)	0.9900
C(1)-C(28)	1.533(2)	C(22)-C(23)	1.397(2)
C(2)-C(3)	1.401(2)	C(22)-C(27)	1.412(2)
C(2)-H(2)	0.9500	C(23)-C(24)	1.393(2)
C(3)-C(4)	1.403(2)	C(23)-H(23)	0.9500
C(3)-C(29)	1.540(2)	C(24)-C(25)	1.410(2)
C(4)-C(5)	1.394(2)	C(24)-C(47)	1.534(2)
C(4)-H(4)	0.9500	C(25)-C(26)	1.399(2)
C(5)-C(6)	1.405(2)	C(25)-H(25)	0.9500
C(5)-C(7)	1.523(2)	C(26)-C(27)	1.396(2)
C(7)-C(8)	1.517(2)	C(26)-C(28)	1.510(2)
C(7)-H(7A)	0.9900	C(28)-H(28A)	0.9900
C(7)-H(7B)	0.9900	C(28)-H(28B)	0.9900
C(8)-C(9)	1.399(2)	C(29)-C(30)	1.531(3)
C(8)-C(13)	1.413(2)	C(29)-C(32)	1.533(3)
C(9)-C(10)	1.400(2)	C(29)-C(31)	1.546(2)
C(9)-H(9)	0.9500	C(30)-H(30A)	0.9800
C(10)-C(11)	1.407(2)	C(30)-H(30B)	0.9800
C(10)-C(36)	1.536(2)	C(30)-H(30C)	0.9800
C(11)-C(12)	1.398(2)	C(31)-H(31A)	0.9800
C(11)-H(11)	0.9500	C(31)-H(31B)	0.9800
C(12)-C(13)	1.408(2)	C(31)-H(31C)	0.9800
C(12)-C(14)	1.514(2)	C(32)-H(32A)	0.9800
C(14)-C(15)	1.532(2)	C(32)-H(32B)	0.9800
C(14)-H(14A)	0.9900	C(32)-H(32C)	0.9800
C(14)-H(14B)	0.9900	C(33)-C(34)	1.475(3)
C(15)-C(20)	1.397(2)	C(33)-H(33A)	0.9900
C(15)-C(16)	1.401(2)	C(33)-H(33B)	0.9900
C(16)-C(17)	1.395(2)	C(34)-C(35)	1.187(2)

C(35)-H(35)	0.9500	C(42)-H(42C)	0.9800
C(36)-C(38)	1.527(3)	C(43)-H(43A)	0.9800
C(36)-C(39)	1.532(2)	C(43)-H(43B)	0.9800
C(36)-C(37)	1.538(2)	C(43)-H(43C)	0.9800
C(37)-H(37A)	0.9800	C(44)-C(45)	1.474(2)
C(37)-H(37B)	0.9800	C(44)-H(44A)	0.9900
C(37)-H(37C)	0.9800	C(44)-H(44B)	0.9900
C(38)-H(38A)	0.9800	C(45)-C(46)	1.191(2)
C(38)-H(38B)	0.9800	C(46)-H(46)	0.9500
C(38)-H(38C)	0.9800	C(47)-C(48)	1.536(2)
C(39)-H(39A)	0.9800	C(47)-C(49)	1.538(2)
C(39)-H(39B)	0.9800	C(47)-C(50)	1.540(2)
C(39)-H(39C)	0.9800	C(48)-H(48A)	0.9800
C(40)-C(43)	1.522(3)	C(48)-H(48B)	0.9800
C(40)-C(41)	1.529(2)	C(48)-H(48C)	0.9800
C(40)-C(42)	1.540(3)	C(49)-H(49A)	0.9800
C(41)-H(41A)	0.9800	C(49)-H(49B)	0.9800
C(41)-H(41B)	0.9800	C(49)-H(49C)	0.9800
C(41)-H(41C)	0.9800	C(50)-H(50A)	0.9800
C(42)-H(42A)	0.9800	C(50)-H(50B)	0.9800
C(42)-H(42B)	0.9800	C(50)-H(50C)	0.9800

C(6)-O(1)-C(33)	116.60(12)	C(6)-C(5)-C(7)	122.24(14)
C(13)-O(2)-H(2O)	109.3(11)	C(1)-C(6)-C(5)	121.67(15)
C(20)-O(3)-C(44)	115.72(12)	C(1)-C(6)-O(1)	120.70(13)
C(27)-O(4)-H(4O)	112.6(12)	C(5)-C(6)-O(1)	117.14(13)
C(6)-C(1)-C(2)	117.72(14)	C(8)-C(7)-C(5)	110.38(13)
C(6)-C(1)-C(28)	122.67(14)	C(8)-C(7)-H(7A)	109.6
C(2)-C(1)-C(28)	119.35(13)	C(5)-C(7)-H(7A)	109.6
C(1)-C(2)-C(3)	122.90(14)	C(8)-C(7)-H(7B)	109.6
C(1)-C(2)-H(2)	118.5	C(5)-C(7)-H(7B)	109.6
C(3)-C(2)-H(2)	118.5	H(7A)-C(7)-H(7B)	108.1
C(2)-C(3)-C(4)	117.00(15)	C(9)-C(8)-C(13)	117.86(15)
C(2)-C(3)-C(29)	123.23(14)	C(9)-C(8)-C(7)	120.91(14)
C(4)-C(3)-C(29)	119.76(14)	C(13)-C(8)-C(7)	121.09(14)
C(5)-C(4)-C(3)	122.44(15)	C(8)-C(9)-C(10)	123.62(15)
C(5)-C(4)-H(4)	118.8	C(8)-C(9)-H(9)	118.2
C(3)-C(4)-H(4)	118.8	C(10)-C(9)-H(9)	118.2
C(4)-C(5)-C(6)	118.22(14)	C(9)-C(10)-C(11)	116.24(15)
C(4)-C(5)-C(7)	119.25(14)	C(9)-C(10)-C(36)	121.89(14)

C(11)-C(10)-C(36)	121.73(15)	C(23)-C(22)-C(27)	117.81(15)
C(12)-C(11)-C(10)	122.96(16)	C(23)-C(22)-C(21)	120.60(15)
C(12)-C(11)-H(11)	118.5	C(27)-C(22)-C(21)	121.43(14)
C(10)-C(11)-H(11)	118.5	C(24)-C(23)-C(22)	123.77(16)
C(11)-C(12)-C(13)	118.53(15)	C(24)-C(23)-H(23)	118.1
C(11)-C(12)-C(14)	121.72(15)	C(22)-C(23)-H(23)	118.1
C(13)-C(12)-C(14)	119.70(14)	C(23)-C(24)-C(25)	116.08(15)
O(2)-C(13)-C(12)	115.67(14)	C(23)-C(24)-C(47)	123.25(15)
O(2)-C(13)-C(8)	123.58(15)	C(25)-C(24)-C(47)	120.67(14)
C(12)-C(13)-C(8)	120.76(14)	C(26)-C(25)-C(24)	122.84(16)
C(12)-C(14)-C(15)	111.34(13)	C(26)-C(25)-H(25)	118.6
C(12)-C(14)-H(14A)	109.4	C(24)-C(25)-H(25)	118.6
C(15)-C(14)-H(14A)	109.4	C(27)-C(26)-C(25)	118.54(16)
C(12)-C(14)-H(14B)	109.4	C(27)-C(26)-C(28)	119.60(14)
C(15)-C(14)-H(14B)	109.4	C(25)-C(26)-C(28)	121.63(15)
H(14A)-C(14)-H(14B)	108.0	O(4)-C(27)-C(26)	115.99(15)
C(20)-C(15)-C(16)	117.84(14)	O(4)-C(27)-C(22)	123.07(15)
C(20)-C(15)-C(14)	122.45(14)	C(26)-C(27)-C(22)	120.95(15)
C(16)-C(15)-C(14)	119.60(14)	C(26)-C(28)-C(1)	110.55(13)
C(17)-C(16)-C(15)	122.77(15)	C(26)-C(28)-H(28A)	109.5
C(17)-C(16)-H(16)	118.6	C(1)-C(28)-H(28A)	109.5
C(15)-C(16)-H(16)	118.6	C(26)-C(28)-H(28B)	109.5
C(16)-C(17)-C(18)	116.94(15)	C(1)-C(28)-H(28B)	109.5
C(16)-C(17)-C(40)	123.13(15)	H(28A)-C(28)-H(28B)	108.1
C(18)-C(17)-C(40)	119.90(14)	C(30)-C(29)-C(32)	109.48(16)
C(19)-C(18)-C(17)	122.66(14)	C(30)-C(29)-C(3)	109.95(14)
C(19)-C(18)-H(18)	118.7	C(32)-C(29)-C(3)	109.01(15)
C(17)-C(18)-H(18)	118.7	C(30)-C(29)-C(31)	106.95(15)
C(18)-C(19)-C(20)	118.09(14)	C(32)-C(29)-C(31)	109.24(15)
C(18)-C(19)-C(21)	119.41(14)	C(3)-C(29)-C(31)	112.17(14)
C(20)-C(19)-C(21)	122.38(14)	C(29)-C(30)-H(30A)	109.5
C(15)-C(20)-C(19)	121.66(14)	C(29)-C(30)-H(30B)	109.5
C(15)-C(20)-O(3)	119.85(13)	H(30A)-C(30)-H(30B)	109.5
C(19)-C(20)-O(3)	118.20(13)	C(29)-C(30)-H(30C)	109.5
C(22)-C(21)-C(19)	110.97(13)	H(30A)-C(30)-H(30C)	109.5
C(22)-C(21)-H(21A)	109.4	H(30B)-C(30)-H(30C)	109.5
C(19)-C(21)-H(21A)	109.4	C(29)-C(31)-H(31A)	109.5
C(22)-C(21)-H(21B)	109.4	C(29)-C(31)-H(31B)	109.5
C(19)-C(21)-H(21B)	109.4	H(31A)-C(31)-H(31B)	109.5
H(21A)-C(21)-H(21B)	108.0	C(29)-C(31)-H(31C)	109.5

H(31A)-C(31)-H(31C)	109.5	C(43)-C(40)-C(41)	109.55(17)
H(31B)-C(31)-H(31C)	109.5	C(43)-C(40)-C(42)	109.19(16)
C(29)-C(32)-H(32A)	109.5	C(41)-C(40)-C(42)	107.49(15)
C(29)-C(32)-H(32B)	109.5	C(43)-C(40)-C(17)	109.47(15)
H(32A)-C(32)-H(32B)	109.5	C(41)-C(40)-C(17)	111.94(14)
C(29)-C(32)-H(32C)	109.5	C(42)-C(40)-C(17)	109.15(15)
H(32A)-C(32)-H(32C)	109.5	C(40)-C(41)-H(41A)	109.5
H(32B)-C(32)-H(32C)	109.5	C(40)-C(41)-H(41B)	109.5
O(1)-C(33)-C(34)	114.07(13)	H(41A)-C(41)-H(41B)	109.5
O(1)-C(33)-H(33A)	108.7	C(40)-C(41)-H(41C)	109.5
C(34)-C(33)-H(33A)	108.7	H(41A)-C(41)-H(41C)	109.5
O(1)-C(33)-H(33B)	108.7	H(41B)-C(41)-H(41C)	109.5
C(34)-C(33)-H(33B)	108.7	C(40)-C(42)-H(42A)	109.5
H(33A)-C(33)-H(33B)	107.6	C(40)-C(42)-H(42B)	109.5
C(35)-C(34)-C(33)	177.52(19)	H(42A)-C(42)-H(42B)	109.5
C(34)-C(35)-H(35)	180.0	C(40)-C(42)-H(42C)	109.5
C(38)-C(36)-C(39)	109.23(16)	H(42A)-C(42)-H(42C)	109.5
C(38)-C(36)-C(10)	111.92(14)	H(42B)-C(42)-H(42C)	109.5
C(39)-C(36)-C(10)	107.63(13)	C(40)-C(43)-H(43A)	109.5
C(38)-C(36)-C(37)	107.19(14)	C(40)-C(43)-H(43B)	109.5
C(39)-C(36)-C(37)	109.18(15)	H(43A)-C(43)-H(43B)	109.5
C(10)-C(36)-C(37)	111.66(14)	C(40)-C(43)-H(43C)	109.5
C(36)-C(37)-H(37A)	109.5	H(43A)-C(43)-H(43C)	109.5
C(36)-C(37)-H(37B)	109.5	H(43B)-C(43)-H(43C)	109.5
H(37A)-C(37)-H(37B)	109.5	O(3)-C(44)-C(45)	110.44(13)
C(36)-C(37)-H(37C)	109.5	O(3)-C(44)-H(44A)	109.6
H(37A)-C(37)-H(37C)	109.5	C(45)-C(44)-H(44A)	109.6
H(37B)-C(37)-H(37C)	109.5	O(3)-C(44)-H(44B)	109.6
C(36)-C(38)-H(38A)	109.5	C(45)-C(44)-H(44B)	109.6
C(36)-C(38)-H(38B)	109.5	H(44A)-C(44)-H(44B)	108.1
H(38A)-C(38)-H(38B)	109.5	C(46)-C(45)-C(44)	179.5(2)
C(36)-C(38)-H(38C)	109.5	C(45)-C(46)-H(46)	180.0
H(38A)-C(38)-H(38C)	109.5	C(24)-C(47)-C(48)	111.60(14)
H(38B)-C(38)-H(38C)	109.5	C(24)-C(47)-C(49)	111.65(14)
C(36)-C(39)-H(39A)	109.5	C(48)-C(47)-C(49)	107.40(15)
C(36)-C(39)-H(39B)	109.5	C(24)-C(47)-C(50)	109.04(14)
H(39A)-C(39)-H(39B)	109.5	C(48)-C(47)-C(50)	108.02(14)
C(36)-C(39)-H(39C)	109.5	C(49)-C(47)-C(50)	109.03(15)
H(39A)-C(39)-H(39C)	109.5	C(47)-C(48)-H(48A)	109.5
H(39B)-C(39)-H(39C)	109.5	C(47)-C(48)-H(48B)	109.5

H(48A)-C(48)-H(48B)	109.5	H(49A)-C(49)-H(49C)	109.5
C(47)-C(48)-H(48C)	109.5	H(49B)-C(49)-H(49C)	109.5
H(48A)-C(48)-H(48C)	109.5	C(47)-C(50)-H(50A)	109.5
H(48B)-C(48)-H(48C)	109.5	C(47)-C(50)-H(50B)	109.5
C(47)-C(49)-H(49A)	109.5	H(50A)-C(50)-H(50B)	109.5
C(47)-C(49)-H(49B)	109.5	C(47)-C(50)-H(50C)	109.5
H(49A)-C(49)-H(49B)	109.5	H(50A)-C(50)-H(50C)	109.5
C(47)-C(49)-H(49C)	109.5	H(50B)-C(50)-H(50C)	109.5

Table S4: Anisotropic displacement parameters ($Å^2$ x 10^3) for **8a**. The anisotropic displacement factor exponent takes the form: $-2\pi^2[h^2 a^{*2}U^{11} + ... + 2 h k a^* b^* U^{12}]$

Atom	U^{11}	U^{22}	U^{33}	U^{23}	U^{13}	U^{12}
O(1)	26(1)	23(1)	21(1)	3(1)	9(1)	2(1)
O(2)	25(1)	26(1)	18(1)	-4(1)	9(1)	-3(1)
O(3)	28(1)	17(1)	24(1)	-2(1)	10(1)	-2(1)
O(4)	27(1)	31(1)	19(1)	6(1)	8(1)	4(1)
C(1)	21(1)	23(1)	18(1)	0(1)	8(1)	0(1)
C(2)	23(1)	26(1)	17(1)	3(1)	7(1)	0(1)
C(3)	26(1)	25(1)	23(1)	1(1)	12(1)	0(1)
C(4)	26(1)	25(1)	20(1)	-5(1)	9(1)	-5(1)
C(5)	23(1)	25(1)	19(1)	-2(1)	10(1)	-1(1)
C(6)	22(1)	21(1)	18(1)	2(1)	10(1)	0(1)
C(7)	23(1)	27(1)	17(1)	-1(1)	5(1)	-4(1)
C(8)	27(1)	23(1)	14(1)	2(1)	7(1)	4(1)
C(9)	22(1)	23(1)	17(1)	2(1)	3(1)	-4(1)
C(10)	24(1)	21(1)	17(1)	1(1)	7(1)	-1(1)
C(11)	32(1)	23(1)	17(1)	3(1)	10(1)	5(1)
C(12)	21(1)	19(1)	16(1)	2(1)	5(1)	0(1)
C(13)	28(1)	20(1)	14(1)	2(1)	5(1)	1(1)
C(14)	26(1)	22(1)	21(1)	1(1)	11(1)	1(1)
C(15)	23(1)	24(1)	21(1)	1(1)	11(1)	-2(1)
C(16)	29(1)	24(1)	22(1)	-3(1)	12(1)	-2(1)
C(17)	29(1)	25(1)	27(1)	2(1)	15(1)	2(1)
C(18)	23(1)	26(1)	24(1)	3(1)	8(1)	0(1)
C(19)	21(1)	23(1)	21(1)	-1(1)	10(1)	-3(1)
C(20)	22(1)	19(1)	24(1)	1(1)	11(1)	-1(1)
C(21)	25(1)	28(1)	18(1)	-1(1)	7(1)	-1(1)
C(22)	25(1)	23(1)	18(1)	-4(1)	8(1)	-3(1)
C(23)	19(1)	26(1)	20(1)	-3(1)	5(1)	-1(1)
C(24)	23(1)	23(1)	17(1)	-3(1)	5(1)	-1(1)
C(25)	30(1)	24(1)	15(1)	-1(1)	10(1)	-1(1)
C(26)	25(1)	21(1)	16(1)	-4(1)	7(1)	-1(1)
C(27)	26(1)	21(1)	15(1)	-2(1)	4(1)	1(1)
C(28)	22(1)	25(1)	16(1)	0(1)	8(1)	0(1)
C(29)	35(1)	25(1)	28(1)	-2(1)	12(1)	2(1)
C(30)	50(1)	38(1)	35(1)	-4(1)	16(1)	9(1)
C(31)	57(2)	30(1)	32(1)	3(1)	11(1)	16(1)

C(32)	55(2)	29(1)	57(1)	5(1)	25(1)	-3(1)
C(33)	26(1)	23(1)	23(1)	0(1)	7(1)	7(1)
C(34)	29(1)	27(1)	19(1)	2(1)	9(1)	5(1)
C(35)	33(1)	39(1)	24(1)	0(1)	7(1)	0(1)
C(36)	30(1)	23(1)	24(1)	-5(1)	10(1)	0(1)
C(37)	35(1)	40(1)	33(1)	-17(1)	13(1)	-11(1)
C(38)	46(1)	39(1)	33(1)	-15(1)	20(1)	-10(1)
C(39)	54(1)	27(1)	38(1)	-5(1)	10(1)	2(1)
C(40)	34(1)	26(1)	35(1)	2(1)	11(1)	5(1)
C(41)	72(2)	24(1)	43(1)	-1(1)	21(1)	9(1)
C(42)	64(2)	28(1)	40(1)	4(1)	18(1)	4(1)
C(43)	42(2)	37(1)	95(2)	3(1)	27(1)	14(1)
C(44)	33(1)	20(1)	24(1)	-1(1)	9(1)	-7(1)
C(45)	27(1)	23(1)	28(1)	-1(1)	12(1)	-4(1)
C(46)	39(1)	32(1)	27(1)	0(1)	12(1)	1(1)
C(47)	24(1)	31(1)	22(1)	3(1)	7(1)	4(1)
C(48)	40(1)	48(1)	32(1)	14(1)	15(1)	12(1)
C(49)	40(1)	43(1)	40(1)	18(1)	10(1)	3(1)
C(50)	55(2)	45(1)	20(1)	6(1)	8(1)	14(1)

Table S5: Hydrogen coordinates (x 10^4) and isotropic displacement parameters (Å^2 x 10^3) for **8a**.

Atom	x	y	z	U(iso)
H(2O)	8519(15)	2860(5)	5050(15)	34
H(4O)	6119(15)	2617(6)	2989(15)	39
H(2)	7593	3937	1494	27
H(4)	9493	4196	4872	28
H(7A)	10300	3114	5756	28
H(7B)	10789	3589	6120	28
H(9)	10162	3961	7426	27
H(11)	7258	3637	7984	28
H(14A)	6070	3038	7014	26
H(14B)	6566	2714	6315	26
H(16)	5234	3757	5846	29
H(18)	3329	3605	2424	29
H(21A)	4170	2525	2214	29
H(21B)	3111	2849	1591	29
H(23)	3211	3306	117	27
H(25)	6198	3507	-401	28
H(28A)	8007	3152	793	25
H(28B)	7983	2796	1709	25
H(30A)	7033	4628	3828	62
H(30B)	7563	5091	3779	62
H(30C)	8352	4738	4665	62
H(31A)	7438	4625	1149	62
H(31B)	7010	5022	1696	62
H(31C)	6481	4556	1709	62
H(32A)	9900	4821	3813	69
H(32B)	9095	5182	2971	69
H(32C)	9529	4783	2437	69
H(33A)	9894	2644	2705	30
H(33B)	10128	2321	3766	30
H(35)	13012	3067	4954	40
H(37A)	10710	4064	9726	54
H(37B)	10416	4546	9947	54
H(37C)	10614	4427	8787	54
H(38A)	7656	4101	9318	57
H(38B)	8703	4372	10235	57
H(38C)	8864	3871	10080	57

H(39A)	8680	4695	7460	63
H(39B)	8506	4872	8590	63
H(39C)	7524	4575	7684	63
H(41A)	4091	4311	5781	70
H(41B)	3918	4750	5078	70
H(41C)	5106	4484	5393	70
H(42A)	4876	4471	3342	67
H(42B)	3721	4756	3043	67
H(42C)	3667	4319	2356	67
H(43A)	2038	4078	2875	87
H(43B)	2090	4495	3640	87
H(43C)	2189	4029	4204	87
H(44A)	4810	1941	3707	31
H(44B)	4034	2254	4144	31
H(46)	6037	1656	7140	39
H(48A)	2252	3675	-1390	60
H(48B)	2405	4097	-2048	60
H(48C)	2952	4078	-658	60
H(49A)	4956	4271	-749	64
H(49B)	4196	4304	-2114	64
H(49C)	5344	4010	-1649	64
H(50A)	4339	3356	-2572	63
H(50B)	3160	3625	-3185	63
H(50C)	3154	3207	-2445	63

Table S6: Torsion angles [°] for **8a**.

C(6)-C(1)-C(2)-C(3)	0.0(2)	C(11)-C(12)-C(14)-C(15)	102.76(17)
C(28)-C(1)-C(2)-C(3)	174.30(15)	C(13)-C(12)-C(14)-C(15)	-74.94(18)
C(1)-C(2)-C(3)-C(4)	0.5(2)	C(12)-C(14)-C(15)-C(20)	113.17(17)
C(1)-C(2)-C(3)-C(29)	-178.35(16)	C(12)-C(14)-C(15)-C(16)	-62.9(2)
C(2)-C(3)-C(4)-C(5)	-1.8(2)	C(20)-C(15)-C(16)-C(17)	-0.7(3)
C(29)-C(3)-C(4)-C(5)	177.05(16)	C(14)-C(15)-C(16)-C(17)	175.55(15)
C(3)-C(4)-C(5)-C(6)	2.6(2)	C(15)-C(16)-C(17)-C(18)	-0.4(3)
C(3)-C(4)-C(5)-C(7)	-171.37(15)	C(15)-C(16)-C(17)-C(40)	-178.44(16)
C(2)-C(1)-C(6)-C(5)	0.8(2)	C(16)-C(17)-C(18)-C(19)	0.2(2)
C(28)-C(1)-C(6)-C(5)	-173.27(15)	C(40)-C(17)-C(18)-C(19)	178.31(15)
C(2)-C(1)-C(6)-O(1)	172.56(14)	C(17)-C(18)-C(19)-C(20)	1.1(3)
C(28)-C(1)-C(6)-O(1)	-1.5(2)	C(17)-C(18)-C(19)-C(21)	-175.08(15)
C(4)-C(5)-C(6)-C(1)	-2.1(2)	C(16)-C(15)-C(20)-C(19)	2.0(2)
C(7)-C(5)-C(6)-C(1)	171.71(15)	C(14)-C(15)-C(20)-C(19)	-174.14(16)
C(4)-C(5)-C(6)-O(1)	-174.11(14)	C(16)-C(15)-C(20)-O(3)	175.72(15)
C(7)-C(5)-C(6)-O(1)	-0.3(2)	C(14)-C(15)-C(20)-O(3)	-0.4(2)
C(33)-O(1)-C(6)-C(1)	82.85(18)	C(18)-C(19)-C(20)-C(15)	-2.2(2)
C(33)-O(1)-C(6)-C(5)	-105.05(16)	C(21)-C(19)-C(20)-C(15)	173.85(15)
C(4)-C(5)-C(7)-C(8)	67.78(19)	C(18)-C(19)-C(20)-O(3)	-176.00(14)
C(6)-C(5)-C(7)-C(8)	-105.95(17)	C(21)-C(19)-C(20)-O(3)	0.0(2)
C(5)-C(7)-C(8)-C(9)	-108.77(16)	C(44)-O(3)-C(20)-C(15)	102.84(16)
C(5)-C(7)-C(8)-C(13)	66.81(18)	C(44)-O(3)-C(20)-C(19)	-83.22(18)
C(13)-C(8)-C(9)-C(10)	1.7(2)	C(18)-C(19)-C(21)-C(22)	72.7(2)
C(7)-C(8)-C(9)-C(10)	177.45(14)	C(20)-C(19)-C(21)-C(22)	-103.26(18)
C(8)-C(9)-C(10)-C(11)	-0.3(2)	C(19)-C(21)-C(22)-C(23)	-103.17(17)
C(8)-C(9)-C(10)-C(36)	-176.13(14)	C(19)-C(21)-C(22)-C(27)	72.16(19)
C(9)-C(10)-C(11)-C(12)	-1.0(2)	C(27)-C(22)-C(23)-C(24)	-0.2(2)
C(36)-C(10)-C(11)-C(12)	174.80(13)	C(21)-C(22)-C(23)-C(24)	175.26(14)
C(10)-C(11)-C(12)-C(13)	0.9(2)	C(22)-C(23)-C(24)-C(25)	-0.3(2)
C(10)-C(11)-C(12)-C(14)	-176.85(13)	C(22)-C(23)-C(24)-C(47)	178.95(14)
C(11)-C(12)-C(13)-O(2)	-179.20(13)	C(23)-C(24)-C(25)-C(26)	-0.1(2)
C(14)-C(12)-C(13)-O(2)	-1.4(2)	C(47)-C(24)-C(25)-C(26)	-179.41(14)
C(11)-C(12)-C(13)-C(8)	0.6(2)	C(24)-C(25)-C(26)-C(27)	1.1(2)
C(14)-C(12)-C(13)-C(8)	178.37(13)	C(24)-C(25)-C(26)-C(28)	-173.37(14)
C(9)-C(8)-C(13)-O(2)	177.96(13)	C(25)-C(26)-C(27)-O(4)	178.19(13)
C(7)-C(8)-C(13)-O(2)	2.2(2)	C(28)-C(26)-C(27)-O(4)	-7.2(2)
C(9)-C(8)-C(13)-C(12)	-1.8(2)	C(25)-C(26)-C(27)-C(22)	-1.7(2)
C(7)-C(8)-C(13)-C(12)	-177.54(14)	C(28)-C(26)-C(27)-C(22)	172.92(14)

C(23)-C(22)-C(27)-O(4)	-178.59(14)
C(21)-C(22)-C(27)-O(4)	6.0(2)
C(23)-C(22)-C(27)-C(26)	1.2(2)
C(21)-C(22)-C(27)-C(26)	-174.21(14)
C(27)-C(26)-C(28)-C(1)	-78.39(18)
C(25)-C(26)-C(28)-C(1)	96.01(17)
C(6)-C(1)-C(28)-C(26)	117.48(16)
C(2)-C(1)-C(28)-C(26)	-56.51(19)
C(2)-C(3)-C(29)-C(30)	130.18(17)
C(4)-C(3)-C(29)-C(30)	-48.6(2)
C(2)-C(3)-C(29)-C(32)	-109.81(19)
C(4)-C(3)-C(29)-C(32)	71.4(2)
C(2)-C(3)-C(29)-C(31)	11.3(2)
C(4)-C(3)-C(29)-C(31)	-167.47(16)
C(6)-O(1)-C(33)-C(34)	60.05(17)
C(9)-C(10)-C(36)-C(38)	-152.69(16)
C(11)-C(10)-C(36)-C(38)	31.8(2)
C(9)-C(10)-C(36)-C(39)	87.29(19)
C(11)-C(10)-C(36)-C(39)	-88.27(18)
C(9)-C(10)-C(36)-C(37)	-32.5(2)
C(11)-C(10)-C(36)-C(37)	151.93(15)
C(16)-C(17)-C(40)-C(43)	-119.0(2)
C(18)-C(17)-C(40)-C(43)	63.0(2)
C(16)-C(17)-C(40)-C(41)	2.7(3)
C(18)-C(17)-C(40)-C(41)	-175.32(15)
C(16)-C(17)-C(40)-C(42)	121.59(17)
C(18)-C(17)-C(40)-C(42)	-56.4(2)
C(20)-O(3)-C(44)-C(45)	-108.05(15)
C(23)-C(24)-C(47)-C(48)	22.6(2)
C(25)-C(24)-C(47)-C(48)	-158.20(15)
C(23)-C(24)-C(47)-C(49)	142.77(16)
C(25)-C(24)-C(47)-C(49)	-38.0(2)
C(23)-C(24)-C(47)-C(50)	-96.70(19)
C(25)-C(24)-C(47)-C(50)	82.55(19)

Table S7: Hydrogen bonds for **8a**[Å and °].

D-H...A	d(D-H)	d(H...A)	d(D...A)	<(DHA)
O(2)-H(2O)...O(1)	0.913(17)	2.001(18)	2.8434(17)	152.8(15)
O(4)-H(4O)...O(3)	0.871(18)	1.969(19)	2.8293(17)	169.2(17)
